# Mechanisms that clear mutations drive field cancerization in mammary tissue

**DOI:** 10.1038/s41586-024-07882-3

**Published:** 2024-09-04

**Authors:** Marta Ciwinska, Hendrik A. Messal, Hristina R. Hristova, Catrin Lutz, Laura Bornes, Theofilos Chalkiadakis, Rolf Harkes, Nathalia S. M. Langedijk, Stefan J. Hutten, Renée X. Menezes, Jos Jonkers, Stefan Prekovic, Grand Challenge PRECISION consortium, Jelle Wesseling, Alastair M. Thompson, Serena Nik-Zainal, Elinor J. Sawyer, Helen R. Davies, Andrew Futreal, Nicholas E. Navin, E. Shelley Hwang, Jos Jonkers, Jacco van Rheenen, Fariba Behbod, Esther H. Lips, Marjanka Schmidt, Lodewyk F. A. Wessels, Daniel Rea, Proteeti Bhattacharjee, Hilary Stobart, Deborah Collyar, Donna Pinto, Ellen Verschuur, Marja van Oirsouw, Benjamin D. Simons, Colinda L. G. J. Scheele, Jacco van Rheenen

**Affiliations:** 1 VIB-KULeuven Centre for Cancer Biology, Department of Oncology, Leuven, Belgium; 2 Division of Molecular Pathology, Oncode Institute, The Netherlands Cancer Institute, Amsterdam, the Netherlands; 3 Centre for Molecular Medicine, UMC Utrecht, Utrecht, the Netherlands; 4 Bioimaging Facility, The Netherlands Cancer Institute, Amsterdam, the Netherlands; 5 Biostatistics Centre and Department of Psychosocial Research and Epidemiology, The Netherlands Cancer Institute, Amsterdam, the Netherlands; 6 Gurdon Institute, University of Cambridge, Cambridge, UK; 7 Cambridge Stem Cell Institute, Jeffrey Cheah Biomedical Centre, University of Cambridge, Cambridge, UK; 8 Department of Applied Mathematics and Theoretical Physics, Centre for Mathematical Sciences, University of Cambridge, Cambridge, UK; 9 The Netherlands Cancer Institute, Amsterdam, the Netherlands; 10 Baylor College of Medicine, Houston, TX USA; 11 University of Cambridge, Cambridge, UK; 12 King’s College London, London, UK; 13 MD Anderson Cancer Center, Houston, TX USA; 14 Duke University School of Medicine, Durham, NC USA; 15 Kansas University Medical Center, Kansas City, KS USA; 16 University of Birmingham, Birmingham, UK; 17 Independent Cancer Patients’ Voice, London, UK; 18 Patient Advocates in Research, Danville, CA USA; 19 DCIS 411, San Diego, CA USA; 20 Borstkankervereniging Nederland, Utrecht, the Netherlands

**Keywords:** Cancer imaging, Self-renewal, Breast cancer

## Abstract

Oncogenic mutations are abundant in the tissues of healthy individuals, but rarely form tumours^1–3^. Yet, the underlying protection mechanisms are largely unknown. To resolve these mechanisms in mouse mammary tissue, we use lineage tracing to map the fate of wild-type and *Brca1*^*−/−*^*;Trp53*^*−/−*^ cells, and find that both follow a similar pattern of loss and spread within ducts. Clonal analysis reveals that ducts consist of small repetitive units of self-renewing cells that give rise to short-lived descendants. This offers a first layer of protection as any descendants, including oncogenic mutant cells, are constantly lost, thereby limiting the spread of mutations to a single stem cell-descendant unit. Local tissue remodelling during consecutive oestrous cycles leads to the cooperative and stochastic loss and replacement of self-renewing cells. This process provides a second layer of protection, leading to the elimination of most mutant clones while enabling the minority that by chance survive to expand beyond the stem cell-descendant unit. This leads to fields of mutant cells spanning large parts of the epithelial network, predisposing it for transformation. Eventually, clone expansion becomes restrained by the geometry of the ducts, providing a third layer of protection. Together, these mechanisms act to eliminate most cells that acquire somatic mutations at the expense of driving the accelerated expansion of a minority of cells, which can colonize large areas, leading to field cancerization.

## Abstract (web summary)

The authors use lineage tracing to map the fate of wild-type and *Brca1*^*−/−*^*;Trp53*^*−/−*^ cells in the adult mouse mammary gland, identifying three layers of protection that limit the spread of mutant cells at the expense of allowing a minority of mutant cells to expand, which leads to field cancerization.

## Main

The acquisition of genetic aberrations in oncogenes and tumour suppressor genes is considered fundamental to tumorigenesis. Seventy per cent of women with germline mutations in *BRCA1* or *BRCA2*, two well-studied tumour suppressor genes, develop breast cancer by the age of 80 (refs. ^4,5^). However, sequencing studies show that mutant cells with alterations in key driver genes, such as *P53*, are abundant in a wide variety of tissues in healthy individuals, including the breast^1–3,6^. This high abundance of mutant cells could be associated with a high frequency of independent mutagenic events, or could arise from a minority of mutant cells that spread over large fields of tissue. With the latter, such behaviour has the potential to create areas predisposed to transformation, a process referred to as field cancerization, and may play a crucial role in the initiation and recurrence of human breast cancer—the ‘sick lobe theory’^7,8^. Yet, despite its importance, the underlying cellular mechanisms that protect breast tissue from the accumulation or spread of mutant cells remain largely unknown.

The mammary epithelium is a branched network of tubes with an outer layer of basal cells and an inner layer of hormone receptor-positive (HR^+^) and -negative (HR^−^) luminal cells. During the 4–7 days of the oestrous cycle, the mouse variant of the menstrual cycle, hormones act on HR^+^ luminal cells, triggering the secretion of mitogenic paracrine signalling factors^9–11^. These factors initiate coordinated rounds of proliferation, also in basal and luminal cells negative for HR, thereby driving the growth of side branches, referred to as alveolar buds. In pregnancy, these side branches progress into lobuloalveolar structures, capable of milk production and secretion^12,13^. However, outside pregnancy, these side branches regress through coordinated cell death at the end of the cycle^14,15^. Thus, throughout life, each oestrous cycle drives coordinated rounds of localized proliferation and cell death^9,16–21^.

Long-term maintenance under conditions of continuous remodelling requires self-renewal activity, and is therefore thought to be driven by adult stem cells^22^. On the basis of transplantation assays and lineage tracing studies, the mouse mammary gland is known to consist of self-renewing unipotent mammary stem cells (MaSCs), also termed enduring progenitors^23^, and their short-lived descendants^14,24–29^. In line with the turnover of side branches, the self-renewing capacity of MaSCs is found to fluctuate during the oestrous cycle^9,19–21,30^. However, the size of MaSC-descendant units and their spatial distribution is as present unknown. As a result, it is unclear whether field cancerization in breast tissue involves the expansion of mutant clones within a single unit or across several units. Moreover, it remains unknown whether and how the normal cellular organization of the mammary gland epithelium protects against the retention of mutant cells and field cancerization. Yet, because the initiation and recurrence of breast cancer may depend on the spread of mutations over larger fields, such protection mechanisms are crucial to resolve. Here, to gain insight into the cellular mechanisms that inhibit the mutant clone expansion, and how they may be overcome by mutant cells to drive field cancerization, we map the fate of cells that acquire mutations in the mouse mammary epithelium.

## Extensive spread of mutant cells precedes tumorigenesis

**Fig. 1:**
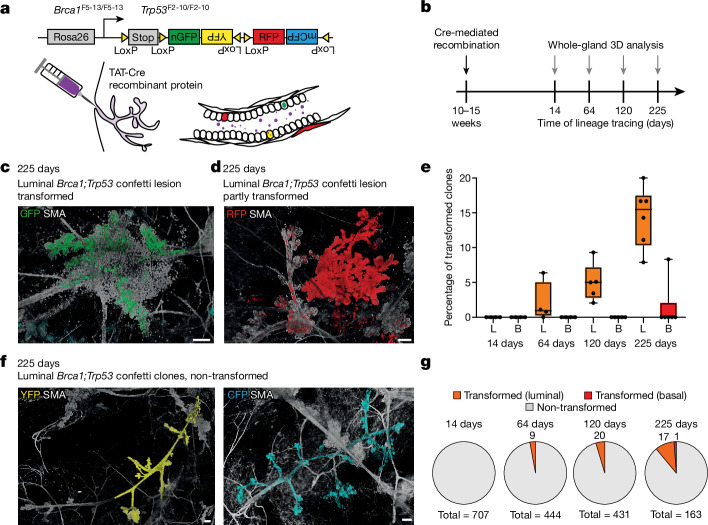
*Brca1*^*−/−*^*;Trp53*^*−/−*^ lesion formation is accompanied by fields of morphologically normal ducts carrying mutant cells. **a**, Schematic of the *Brca*^*fl/fl*^*;Trp53*^*fl/fl*^*;R26R-Confetti* mouse model used in this study. Recombination was induced by an intraductal injection method with TAT-Cre recombinant protein, leading to sporadic deletion of the *Brca1;Trp53* alleles and at the same time stochastic recombination of the *Confetti* construct resulting in the expression of one of the four fluorophores. **b**, Timeline of lineage tracing experiments performed in the adult mammary gland. **c**,**d**, *Brca1;Trp53* confetti lesions with a transformed ductal morphology (**c**) and partially transformed ductal morphology and local invasion (**d**). **e**, Transformed luminal (L, orange) and basal (B, red) clones as a percentage of the total number of luminal and basal clones, respectively. Each dot indicates an individual mouse; boxplots mark the 25th and 75th percentile, the line indicates the median and the whiskers mark the minimum and maximum values. **f**, Representative whole-mount confocal images of non-transformed *Brca1*^*−/−*^*;Trp53*^*−/−*^ confetti clones showing extensive field cancerization within the existing ductal structure. **c**,**d**,**f**, Images show 3D rendering of *Z*-stacks, with the confetti-labelled cells in their respective colours and the mammary ducts labelled with an antibody against SMA (white). Representative examples of *n* = 6 mice. **g**, Charts representing the fraction of non-transformed luminal and basal clones (grey), the transformed luminal clones (orange) and the transformed basal clones (red) at different time points after recombination for all analysed glands combined. The total number of quantified *Brca1*^*−/−*^*;Trp53*^*−/−*^ confetti clones is indicated below the charts and the number of transformed clones is indicated within the charts. See Supplementary Information 1 for sample sizes and descriptive statistics for **e** and **g**. Scale bars, 100 μm. Source Data

**Extended Data Fig. 1:**
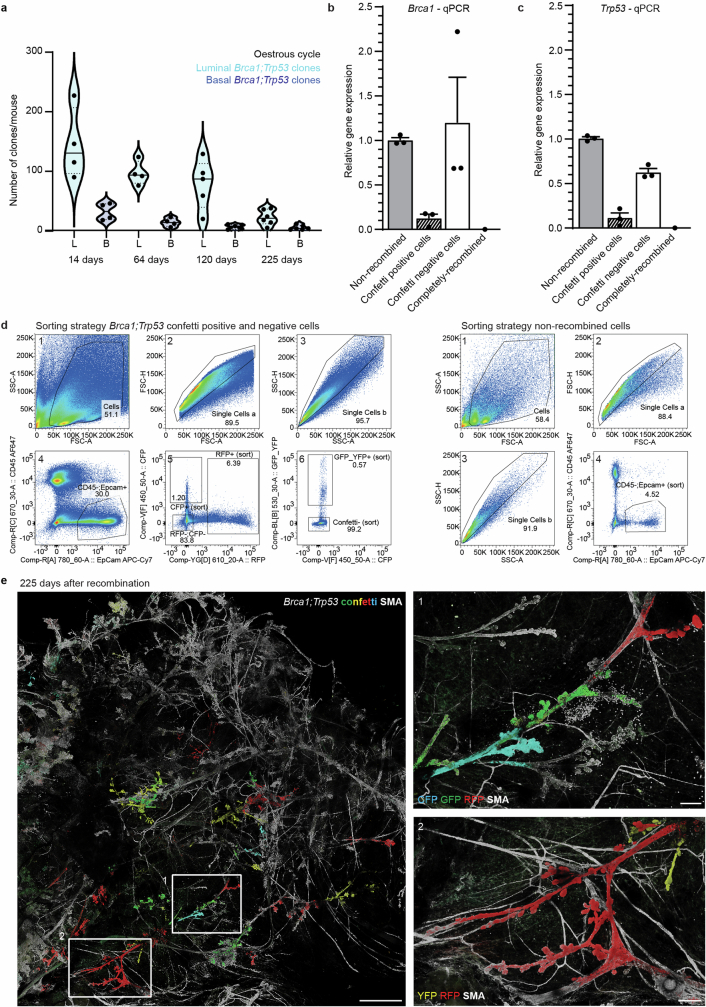
Stochastic recombination in the *Brca1*^*fl/fl*^*;Trp53*^*fl/fl*^*;R26R-Confetti* mouse model. **a**, Quantification of the total number of *Brca1;Trp53* confetti clones after TAT-Cre mediated recombination in at least 4 different 4th mammary glands derived from different mice. Number of clones was determined by using large tilescans (xyz) of the entire mammary glands (whole-mount) labelled with SMA (basal cells) or ECAD (luminal cells). Luminal clones are depicted in cyan, basal clones are depicted in blue. **b**, qPCR for the *Brca1* allele in sorted confetti-positive cells and sorted confetti-negative cells derived from TAT-Cre recombined *Brca1*^*fl/fl*^*;Trp53*^*fl/fl*^*;R26R-Confetti* mammary glands and a positive control (*Brca1* ^*Δ /Δ*^) normalized to a non-recombined control (*Brca1*^*F/F*^). The recombined samples demonstrate that the vast majority of confetti-positive cells have fully recombined *Brca1* alleles. *n* = 3 biological replicates. Each dot represents a biological replicate and error bars indicate s.e.m. **c**, qPCR for the *Trp53* allele in sorted confetti-positive cells and sorted confetti-negative cells derived from TAT-Cre recombined *Brca1*^*fl/fl*^*;Trp53*^*fl/fl*^*;R26R-Confetti* mammary glands and a positive control (*Trp53* ^*Δ /Δ*^) normalized to non-recombined control (*Trp53*^*F/F*^). The recombined samples demonstrate that the vast majority of confetti-positive cells have fully recombined *Trp53* alleles. Note that the confetti-negative cells show some loss of the *Trp53* allele as well. Each dot represents a biological replicate and error bars indicate s.e.m. *n* = 3 biological replicates. **d**, Representative plots depicting the gating strategy to sort the Confetti positive and negative mammary epithelial cells from TAT-Cre recombined *Brca1;Trp53*;*R26R-Confetti* mammary glands (left panels) and mammary epithelial cells from non-recombined mammary glands (right panels). Sorted cells were used to determine *Brca1* and *Trp53* gene levels by qPCR in Extended Data Fig. 1b and c. Numbers in panels indicate order of gating. The *Brca1;Trp53* mammary cells in the RFP-CFP- gate in panel 5 were selected to sort GFP_YFP+ and Confetti- cells in panel 6. **e**, 3D rendering of a Z-stack confocal image of a whole-mount *Brca1;Trp53* confetti mammary gland 225 days after recombination labelled with SMA, confetti clones are represented in their respective colours. *Brca1;Trp53* mutant confetti clones are distributed throughout the mammary ductal tree and span large areas of the ducts without changing the ductal morphology. Note the recruitment of SMA-positive stromal cells near the GFP clone in panel 1. Scale bar represents 1 mm (overview image) and 100 µm (panel 1 and 2). Representative image of *n* = 6 mice. Source Data

**Extended Data Fig. 2:**
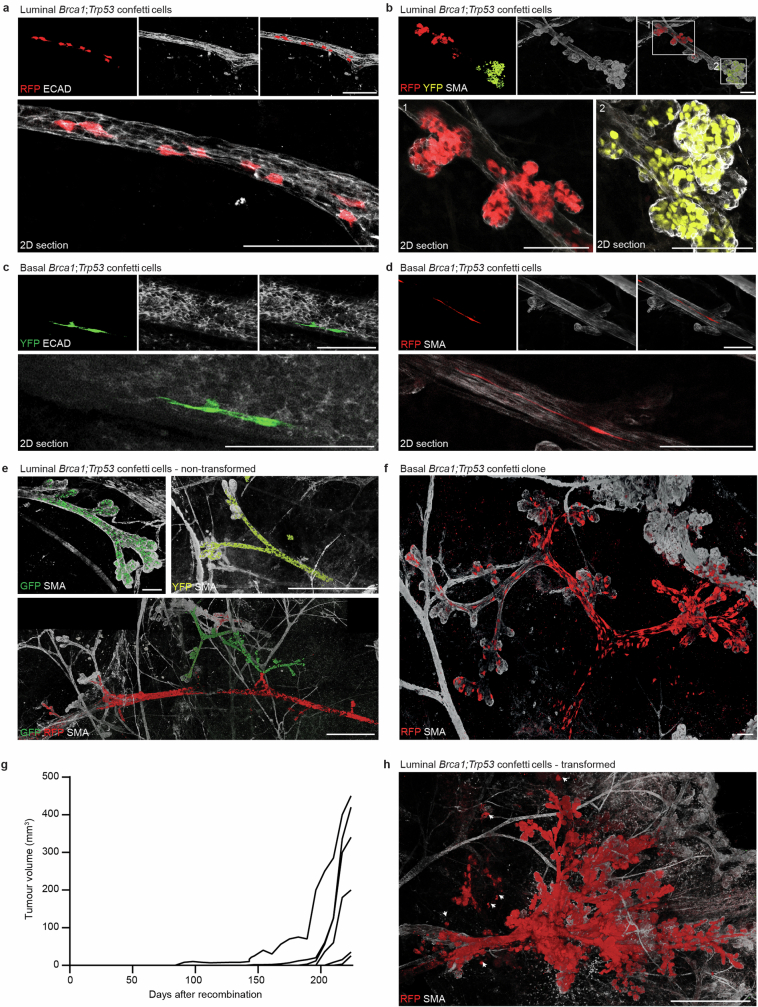
Characterization of *Brca1;Trp53* confetti clones using antibody labelling in intact mammary glands. **a**, **b**, Whole-mount confocal images (3D rendering of a Z-stack in top panels and a representative 2D section of the Z-stack in the bottom panel) of luminal *Brca1;Trp53* confetti cells. Luminal confetti cells show overlap with E-cadherin (ECAD) labelling (**a**) and no overlap with alpha-smooth muscle actin (SMA) expression (**b**) depicted in white. Scale bars represent 100 µm. **c,** **d**, Whole-mount confocal images (3D rendering of a Z-stack in top panels and a representative 2D section of the Z-stack in the bottom panel) of basal *Brca1;Trp53* confetti cells. Basal confetti cells show no overlap with E-cadherin (ECAD) labelling (**c**) and overlap with alpha-smooth muscle actin (SMA) expressing cells (**d**) depicted in white. Scale bars represent 100 µm. **e**, **f**, Representative whole-mount confocal images (3D rendering of a Z-stack) showing luminal (**e**) and basal (**f**) *Brca1;Trp53* confetti cells that expanded within the ducts leading to clonal fields of mutant cells without morphological transformation of the ducts. Ducts are labelled with SMA, depicted in white. Scale bars represent 100 µm (e, left top panel) or 1 mm (other panels). **g**, Tumour growth dynamics of palpable transformed lesions within the *Brca1;Trp53* confetti model after recombination. **h**, Representative whole-mount confocal image (3D rendering of a Z-stack) showing a *Brca1;Trp53* luminal confetti clone that expanded within the ducts leading to transformation of the ductal morphology (hyperbranching), including local invasion (white arrows denote *Brca1;Trp53* RFP cells within the stroma). Ducts are labelled with SMA, depicted in white. Scale bar represents 1 mm. All images represent *n* ≥ 4 biological repeats (mice).

To map the fate of mutant clones, we studied confetti mice carrying homozygous floxed alleles of the tumour suppressor genes *Brca1* and *Trp53* (Fig. 1a). At the beginning of adulthood (between 10 and 15 weeks)^31^, luminal and basal cells were recombined at a low induction frequency by intraductal TAT-Cre injection (Fig. 1a,b), with a bias towards luminal cells (Extended Data Fig. 1a). By quantitative PCR (qPCR) we confirmed that, in more than 87% of confetti-labelled cells, the *Brca1* or *Trp53* gene was recombined (Extended Data Fig. 1b–d). By contrast, most confetti-negative cells were wild-type (WT), although a fraction of these cells also harboured recombined *Brca1* or *Trp53* genes (Extended Data Fig. 1b–d), indicating that, in some cases, mutant confetti clones neighbour an unlabelled mutant clone. By imaging the whole mammary glands at cellular resolution, we observed outgrowth of clones throughout the ductal tree, without preferential localization (Extended Data Fig. 1e). No stromal recombination was detected (Extended Data Fig. 1e). While multipotent clones have been reported in the adult mammary gland^27,29^, especially under mutant conditions or following widespread damage^32–35^, we found only lineage-restricted clones based on E-cadherin (ECAD) staining for luminal cells and α-smooth muscle actin (SMA) staining for basal cells (Extended Data Fig. 2a–f). However, a potential minority of the multipotent population may be missed by the low induction frequency. Moreover, as *Brca1;Trp53* confetti clones are genomically unstable, we cannot exclude the possibility that some of the luminal clones originate from basal cells that acquired multipotency as a result of accumulating genetic alterations.

As reported previously^31^, within 200–250 days of induction, mice developed palpable mammary tumours (Extended Data Fig. 2g). At the microscopic level, transformation was determined on the basis of ductal deformation (Fig. 1c), aberrant branch formation (Fig. 1d) and (local) invasion (Fig. 1d and Extended Data Fig. 2h). Most transformed clones were of luminal origin (Fig. 1e), in line with previous reports^36–39^. We also observed many large non-transformed *Brca1;Trp53* confetti clones (Fig. 1f,g and Extended Data Figs. 1e and 2e,f). Quantification showed that only a minority of mutant clones transitioned to a transformed phenotype at 225 days post-induction (Fig. 1g). In contrast to transformed *Brca1;Trp53* confetti clones, non-transformed clones did not alter the ductal morphology, but extended over large areas of normal-looking ducts, as previously suggested by the sick lobe theory^7,8^.

**Extended Data Fig. 3:**
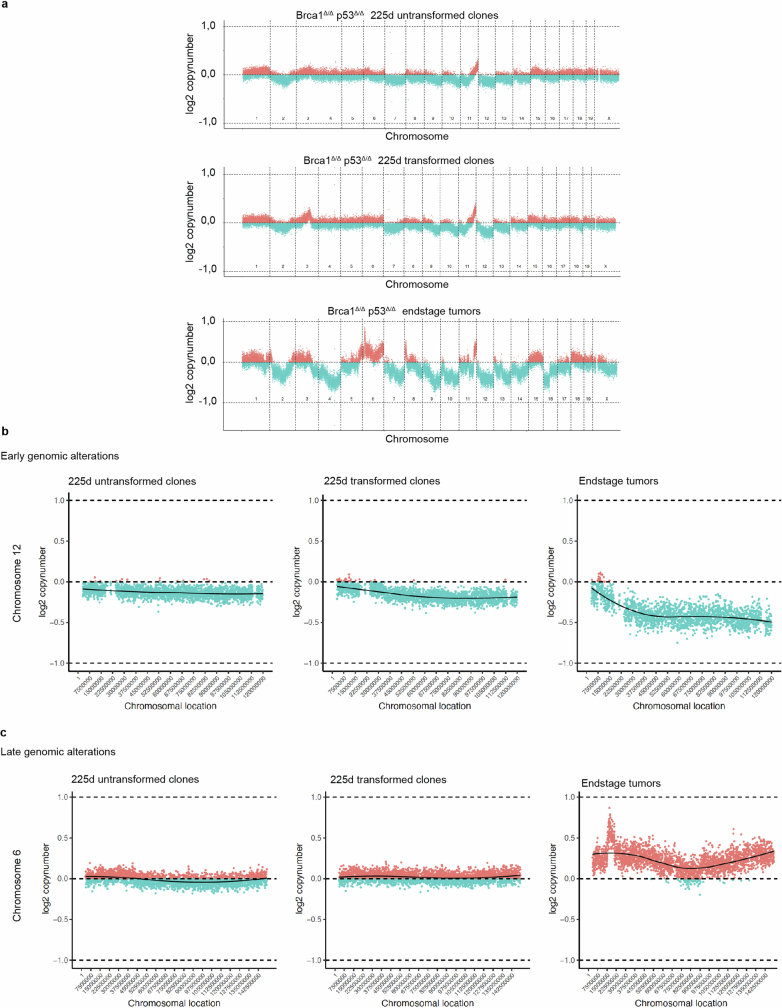
Genomic alterations in *Brca1;Trp53* confetti clones and end stage tumours. **a**, DNA copy number profiles in untransformed (top) and transformed (middle) *Brca1;Trp53* confetti clones 225 days after Cre-recombination, and in *Brca1;Trp53* end-stage tumours (bottom). **b**, Example of genomic events that occur early in *Brca1;Trp53* tumorigenesis. DNA copy number profiles of chromosome 12 showing genomic losses that are found in early transformed and untransformed *Brca1;Trp53* clones, as well as in end-stage tumours. **c**, Example of genomic events occurring late in *Brca1;Trp53* tumorigenesis. DNA copy number plots of chromosome 6 showing copy number changes that are unique to end-stage *Brca1;Trp53* tumours, but not found in early clones. All plots show averages of 3 mice (225d timepoint: 13 transformed clones and 13 untransformed clones) and 10 mice (end-stage tumours).

Next, we isolated both normal-looking and transformed *Brca1;Trp53* clones and performed low-coverage DNA sequencing. As anticipated for genetically unstable *Brca1;Trp53* clones, we identified a heterogeneous but substantial number of chromosomal aberrations in both normal-looking and transformed clones (Supplementary Information 3, copy number aberrations (CNA)). When examining the average CNA of all clones, common patterns of chromosomal loss emerged (Extended Data Fig. 3a). To assess whether these patterns were also present in fully developed late-stage tumours, we compared our sequencing data with published CNA data from late-stage *Brca1;Trp53* tumours^40^. Many genomic regions that are lost in the late-stage tumours were already lost in normal-looking and transformed clones (Extended Data Fig. 3a,b). At the same time, late-stage tumours also featured chromosomal gains, which were not yet present in our early clones (Extended Data Fig. 3c). This suggests that the loss of genomic regions is an early event in *Brca1;Trp53*-driven tumorigenesis, occurring even before transformation, whereas genomic amplifications represent late-stage events in this tumour model. Moreover, these data illustrate that we are studying the earliest phases of tumorigenesis, in which clones have already accumulated large genomic alterations, yet still present a normal phenotype.

## Spread of mutant cells is intrinsic to ductal turnover

**Fig. 2:**
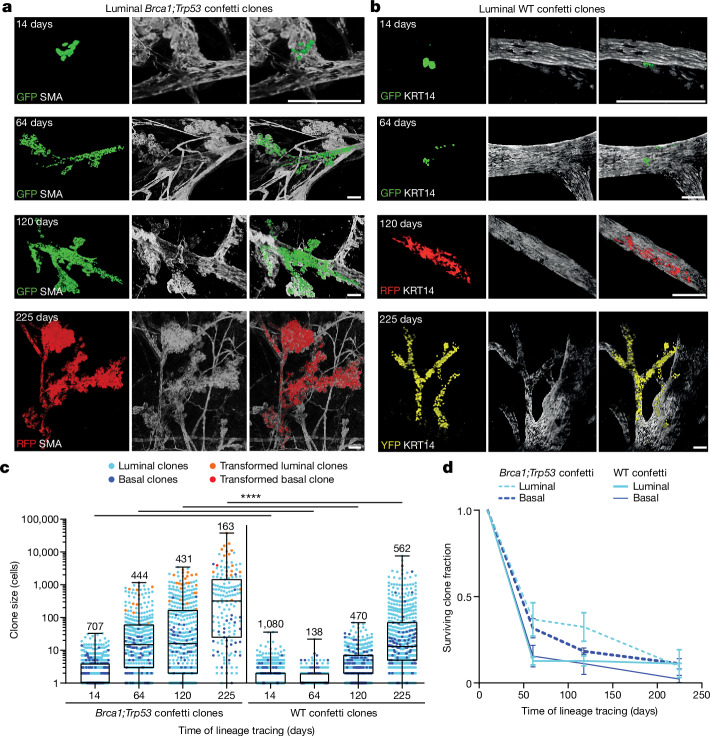
Long-term unbiased lineage tracing in adult mammary gland under mutant and homeostatic conditions. **a**,**b**, Representative confocal whole-mount images showing clonal expansion of luminal *Brca1;Trp53* confetti clones (**a**) and luminal WT confetti clones (**b**) in the adult mammary gland over a lineage tracing time period of 225 days. Persisting clones form cohesive clusters of cells spanning many ducts and branch points. Images show 3D rendering of *Z*-stacks, with the confetti-labelled cells in their respective colours and the mammary ducts labelled with SMA or Keratin 14 (KRT14), both depicted in white. **c**, Clone size quantification of luminal (cyan dots) and basal (blue dots) *Brca1;Trp53* confetti clones (left) and WT confetti clones represented on a logarithmic scale. For each time point at least *n* = 6 glands from three mice were analysed. Morphologically transformed clones are indicated in orange (luminal) and red (basal). The analysed numbers of clones for each time point are indicated. Boxplots mark the 25th and 75th percentiles, the line indicates the median and the whiskers mark the minimum and maximum values. Significance was tested using a two-sided Mann–Whitney test, ****P < 0.0001. **d**, Average surviving basal and luminal clone fraction as a function of time normalized to the average number of confetti^+^ cells 14 days after recombination. Each data point shows the average of at least *n* = 3 mice per time point. Error bars represent ±s.e.m. From a longitudinal data analysis between the *Brca1;Trp53* and WT clones there is no significant difference between the groups (Supplementary Information 2). See Supplementary Information 1 for sample sizes, *P* values and statistics for **c** and **d**. Scale bars, 50 µm. Source Data

**Extended Data Fig. 4:**
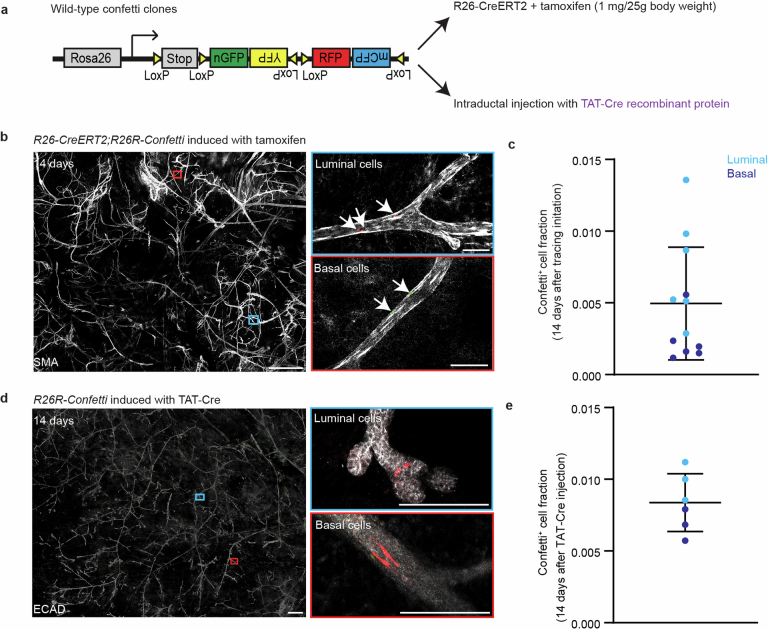
Recombination of wild-type confetti clones with TAT-Cre or tamoxifen results in similar labelling efficiencies. **a**, Schematic representation of the *R26R-Confetti* construct (left), which was recombined sporadically through an intraperitoneal injection with a low dose of tamoxifen in the presence of *R26-CreERT2 (R26R-Confetti*^*het*^*;R26-CreERT2*^*het*^ mouse model), which is the gold standard method, or through intraductal injection of TAT-Cre recombinant protein. **b**, Confocal overview image of a whole-mount 4th mammary gland derived from a *R26R-Confetti*^*het*^*;R26-CreERT2*^*het*^ adult female mouse 14 days after tracing initiation by tamoxifen-mediated recombination. Zooms show ducts containing single confetti-labelled cells of both basal and luminal origin, representative of the initial labelling density after tracing initiation. Ductal tree is stained with alpha-smooth muscle actin (SMA) depicted in white, which marks the basal cell layer. Scale bar left image represents 1 mm, scale bar right images represents 100 µm. **c**, Quantification of the confetti-positive cell fraction 14 days after tamoxifen-mediated recombination. Each dot represents the fraction of recombined cells in a randomly selected area within each mammary gland of approximately 1 ×1 mm, *n* = 6 glands derived from 6 different mice. Basal cells are normalized to the total number of basal cells in the selected area (blue dots) and luminal cells are normalized to the total number of luminal cells in the selected area (cyan dots). Error bar represents mean ± s.d. **d**, Confocal overview image of a whole-mount 4th mammary gland derived from a *R26R-Confetti*^*het*^ adult female mouse, 14 days after tracing initiation recombined by the TAT-Cre intraductal injection method. Zooms show ducts containing single confetti-labelled cells of both basal and luminal origin, representative of the initial labelling density after tracing initiation. Ductal tree is stained with E-cadherin (ECAD) depicted in white, which marks the luminal cell layer. Scale bar left image represents 1 mm, scale bar right images represents 100 µm. **e**, Quantification of the confetti-positive cell fraction 14 days after TAT-Cre-mediated recombination for basal (blue dots) and luminal (cyan dots) cells. Each dot represents the fraction of recombined cells in a randomly selected area within each mammary gland of approximately 1 ×1 mm, *n* = 3 glands derived from 3 different mice. Error bar represents mean ± s.d. Note that recombination efficiencies of luminal and basal cell populations are similar between the tamoxifen- and TAT-Cre-induced recombination techniques. Both induction methods result in the recombination of approximately one labelled cell for every 100–200 cells. As the Confetti construct comprises four distinct colours, there is, on average, one cell labeled with a confetti colour per 400–800 cells. Considering that a MaSC-progeny unit consists of approximately 5 to 10 cells, a single confetti-labeled cell is induced in 1 out of 40–80 units. Importantly, over time, many clones become extinct (Fig. 2d), leading to a dilution in the number of clones and making collisions even less likely. Source Data

**Extended Data Fig. 5:**
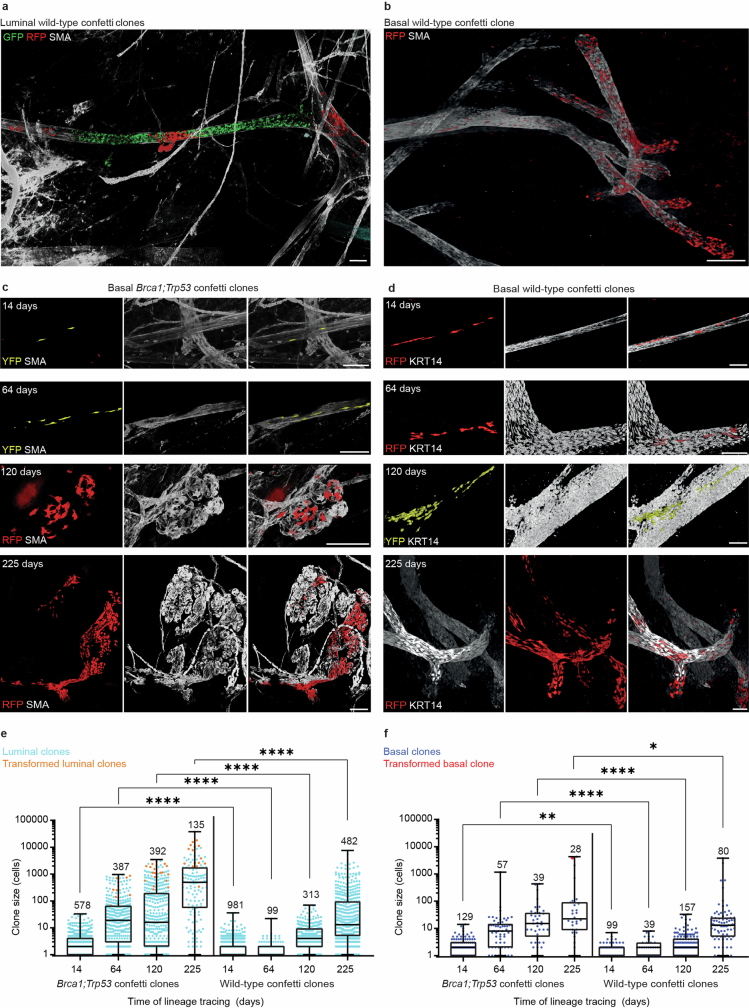
Wild-type and *Brca1;Trp53* basal and luminal confetti cells form large cohesive clones spanning multiple ducts and branch points. **a**, **b**, Representative whole-mount confocal images of wild-type luminal confetti clones (**a**) and wild-type basal confetti clones (**b**) showing extensive field clonalization within the existing ductal structure. Ducts are labelled with alpha-smooth muscle actin (SMA), confetti fluorophores are represented in their respective colours. Scale bars represent 100 µm. Images in a and b represent *n* ≥ 3 biological repeats (mice). **c**, Representative whole-mount confocal images (3D rendering of Z-stacks) of basal *Brca1;Trp53* confetti clones at different timepoints after recombination showing clonal expansion within the ductal tree over a period of 225 days. Ducts are labelled with SMA. **d**, Representative whole-mount confocal images of wild-type basal confetti clones showing extensive field clonalization within the existing ductal structure over a period of 225 days. Ducts are labelled with Keratin 14 (KRT14). **c**, **d**, Persisting clones form cohesive clusters of cells spanning multiple ducts and branch points. Scale bars represent 100 µm. **e**, **f**, Quantification of clone sizes in the *Brca1;Trp53* and wild-type confetti conditions at different timepoints after tracing initiation for the luminal (**e**) and basal (**f**) clones separately. The number of quantified clones is indicated within the graph, transformed clones are shown in orange (luminal) and red (basal). Boxplots mark the 25th and 75th percentile, line indicates the median, and whiskers mark the minimum and maximum values. Significance was tested using a two-sided Mann-Whitney test, * *P* < 0,05, ** *P* < 0.01, **** *P* < 0.0001. Same data as depicted in Fig. 2c. See Supplementary Information 1 for more sample sizes, *P* values and statistics for **e** and **f**. Source Data

To understand what drives clonal expansion, we compared the spread of mutant confetti clones with WT confetti clones (that is, recombined cells in *R26R-Confetti* mice). Sporadic recombination of WT cells was induced throughout the ductal tree by either intraductal injection of TAT-Cre recombinant protein or activation of *CreERT2* with a low dose of tamoxifen (Extended Data Fig. 4a–e). Similar to the *Brca1;Trp53* confetti clones, we found that WT clones also showed the capacity to spread extensively, occupying substantial areas of the ductal network (Fig. 2a,b and Extended Data Fig. 5a–d). Hereafter, we refer to this process as ‘field clonalization’. To study their spread, we quantified the size of 2,250 WT confetti clones in 75 glands of 17 mice using whole-gland three-dimensional (3D) imaging (Fig. 2b,c and Extended Data Fig. 5b,d–f). Despite differences in the relative labelling efficiency between the luminal and basal lineage (Extended Data Figs. 1a and 4c,e), WT and mutant clones showed a similar size distribution, suggesting a common mechanism of expansion. Moreover, the sparsity of labelling and the cohesive nature of clones provided confidence in the integrity of clonal assignments (Extended Data Fig. 4c,e). Together, these data suggest that, similar to *Brca1;Trp53* confetti-labelled cells, WT clones can spread over large areas of the ductal network, leading to field clonalization.

## Ductal turnover in small MaSC-descendant units

**Extended Data Fig. 6:**
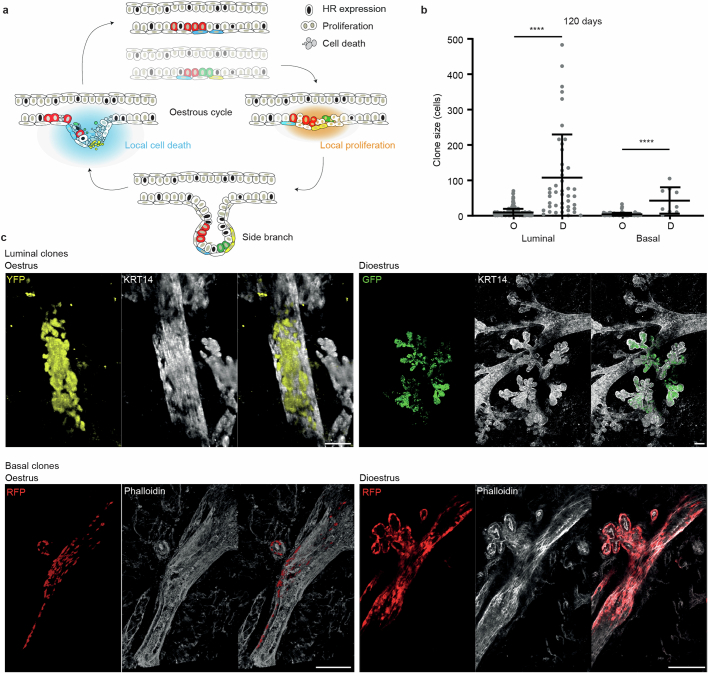
Wild-type clone sizes transiently increase during each oestrous cycle. **a**, Schematic depicting the cellular basis of the cell-based model of mammary epithelial turnover (see also Extended Data Fig. 9b). Note that, during one round of oestrous cycle, some clones are collectively lost (e.g., yellow and green clone), while others expand (e.g., blue and red clones). **b**, Quantification of wild-type confetti^+^ clone sizes during oestrus (O) and dioestrus (D) stage 120 days after lineage tracing initiation, demonstrating a temporary increase of clone sizes during dioestrus stage. *n* = 3 mice for oestrus stage (300 luminal clones, 101 basal clones) and *n* = 3 mice for dioestrus stage (43 luminal clones, 9 basal clones). Error bars represent mean ± s.d. Significance was tested using a two-sided Mann-Whitney test, **** *P* < 0.0001. **c**, Representative whole-mount confocal images of wild-type confetti^+^ clones 120 days after lineage tracing initiation during oestrus (left panels) and dioestrus stage (right panels). Both luminal (top panels) and basal clones (bottom panels) show an increase in clone size during dioestrus stage. Confetti-labelled cells are depicted in their respective colour, and the mammary ducts are labelled with Keratin 14 (KRT14) or Phalloidin in white. Scale bars represent 100 µm. Source Data

**Extended Data Fig. 7:**
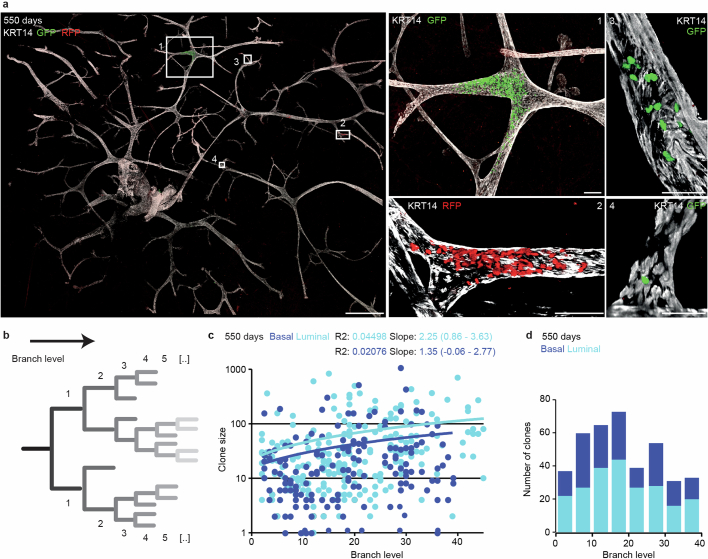
Spatial and size distribution of wild-type confetti clones in the mammary gland. **a**, Representative confocal overview image of a whole mount 5th mammary gland after 550 days of lineage tracing, illustrating the distribution of wild-type confetti clones within the ductal tree. Images depict 3D-rendering of Z-stacks, with the confetti labelled cells in their respective colour and the mammary ducts labelled with Keratin 14 (KRT14) shown in white. Scale bars represent 1 mm (left panel), 100 µm (panel 1 and 2), and 50 µm (panel 3 and 4). Representative image of *n* = 8 glands from 4 biological repeats (mice). **b**, Branch levels are defined as the number of branch points starting from the main duct close to the nipple. **c**, Quantification of the wild-type confetti clone size by branch level. Each dot represents a clone, cyan dots for luminal clones and blue dots for basal clones. Line indicates linear regression of the luminal and basal clone sizes with R^2^, slope and 95% confidence interval of the slope indicated in the graph, *n* = 6 glands from 3 mice. **d**, Number of wild-type confetti clones represented by bars for each branch level after 550 days of tracing in *n* = 6 glands from 3 mice. Source Data

Next, we questioned the factors that drive field clonalization. The mammary epithelium contains a hierarchy of MaSCs^23^ and their short-lived descendants^19,21,24,25^. It is therefore expected that confetti-labelled WT descendants should be lost over time, whereas MaSC-derived clones should spread to their descendants, potentially leading to field clonalization. To test this hypothesis, we quantified the size and position of WT confetti clones over time. Because clones were found to change transiently during the oestrous cycle as a result of growth and regression of side branches^9,19,21^ (Extended Data Fig. 6a–c), quantifications were made at the oestrus stage to avoid potential inconsistencies. Consistent with a MaSC-descendant hierarchy, we found that the number of surviving WT confetti clones showed a steep decrease in the first few months following induction (Fig. 2d). Between 10 and 20% of stochastically recombined cells seemed to show long-term renewal capacity, whereas 80–90% were relatively short-lived, consistent with a small MaSC-descendant hierarchy of five to ten cells. With ductal homeostasis supported by repeated small MaSC-descendant units, we reasoned that MaSCs must be distributed widely throughout the ductal network. To test this, we reconstructed the topology of the ductal network (Extended Data Fig. 7a) and identified for each WT clone its relative position within the tree based on its ‘branch level’, defined as the number of branch points that separate the clone from the main duct close to the nipple (Extended Data Fig. 7b). This analysis indeed showed that WT clones were scattered uniformly throughout the ductal network, even after 550 days of tracing (Extended Data Fig. 7c,d).

Altogether, our data showed that the behaviour of WT clones is in line with a model in which the homeostatic renewal of the mammary epithelium is organized in repetitive MaSC-descendant units that are distributed evenly throughout the ductal network. This tissue hierarchy supports a model in which field clonalization is preceded by the loss of most mutations that are acquired in short-lived descendants, followed by the spread of mutations from the randomly distributed MaSCs to their adjacent short-lived progenies.

It remains to be determined how the MaSC populations labelled by our unbiased lineage tracing approach relate to the populations identified through promoter-specific tracing strategies, including those based on *Bcl11b*, *Tspan8* or *ProcR* expression^29,41,42^. For example, long-lived, quiescent stem cells have been identified in label-retaining assays, and might have a specialized function under perturbed conditions, such as pregnancy or repair^35,41,42^.

## Clonal spread follows a simple statistical rule

**Extended Data Fig. 8:**
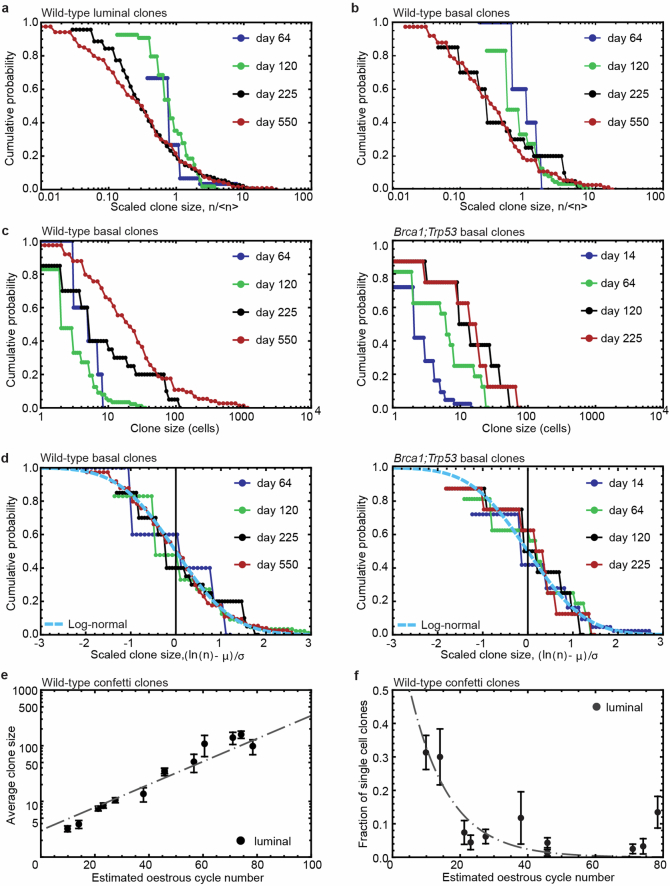
Wild-type and *Brca1;Trp53* confetti clones follow a log-normal distribution. **a**, **b**, Cumulative distribution of luminal (**a**) and basal (**b**) wild-type confetti clone size as a function of the scaled clone size *n*/⟨*n*⟩, where ⟨*n*⟩ denotes the average clone size. To account for the impact of large-scale mouse-to-mouse variability in clone size, curves are shown for a representative set of individual mice (shown in Fig. 3a, b) with corresponding distributions shown for all mice in Supplementary Information 4. Note that the data does not show evidence for collapse towards a statistical scaling behavior, as would be predicted for clonal dynamics based on local stochastic stem cell loss and replacement (see main text and Supplementary Information 4). *n* ≥ 3 mice per time point. **c**, Cumulative distribution of wild-type (left) and *Brca1;Trp53* (right) basal confetti clone size showing the probability of finding a clone larger than the given size (log scale) across time points. To account for the impact of large-scale mouse-to-mouse variability in clone size, the curves are shown for a representative set of individual mice with corresponding distributions shown for all mice in Supplementary Information 4. *n* ≥ 3 mice per time point. **d**, Rescaled cumulative distribution of the logarithm of wild-type (left) and *Brca1;Trp53* (right) basal confetti clone size, ln *n*, showing the probability of finding a clone with a size larger than $({\rm{l}}{\rm{n}}\,n-\mu )/\sigma$, where $\mu =\langle {\rm{l}}{\rm{n}}\,n\rangle$ denotes the average of the logarithm of clone size and ${\sigma }^{2}=\langle ({\rm{l}}{\rm{n}}\,n-\langle {\rm{l}}{\rm{n}}\,n\rangle {)}^{2}\rangle$ represents the variance. Points show data from panel (**d**). Once rescaled, data from different time points collapse onto a single curve that fits well with the scaling function $(1/2){\rm{erfc}}(x/\sqrt{2})$ (dashed line), consistent with a log-normal size dependence (see main text). For details of statistical significance tests, see Supplementary Information 4. **e**, Average luminal clone size as a function of the inferred oestrous cycle number for wild-type confetti clones. Points show data from individual mice and line shows the theoretical prediction of the model. (Note that the average of the logarithm of clone size is not the same as the logarithm of the average.) **f**, Fraction of single-cell luminal wild-type confetti clones as a function of inferred oestrous cycle number. Points show data and line (dashed) shows theoretical prediction of the model based on the fits in Fig. 3c, d. Bars in **e** and **f** denote mean values +/− SEM. For details of the model, the model fits, and the inference of oestrous cycle number, see main text and Supplementary Information 4. Source Data

In an MaSC-descendant unit of five to ten cells, a WT clone originating from an MaSC could never exceed around ten cells. However, we observed clones that become hundreds of times larger (Fig. 2c). In other hierarchically organized epithelial tissues, clones can continually expand through a ‘neutral’ process of stochastic stem cell loss and replacement^43^. In homeostasis, such behaviour finds a signature in the statistical scaling behaviour of clone sizes^44^. However, when analysing the distribution of WT clones, we could not find evidence for such statistical scaling behaviour in individual animals across different time points (Extended Data Fig. 8a,b and Supplementary Information 4).

**Fig. 3:**
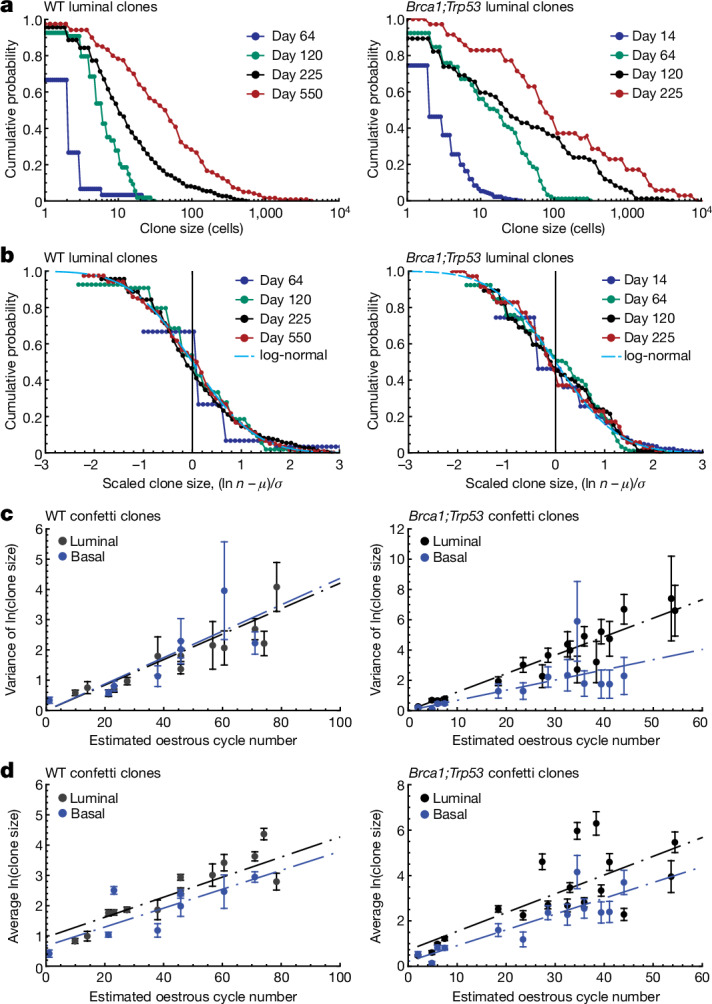
Clone sizes follow a log-normal distribution. **a**, Cumulative distribution of WT (left) and *Brca1;Trp53* (right) luminal confetti clone sizes showing the probability of finding a clone larger than the given size (log scale). To account for the impact of large-scale mouse-to-mouse variability on clone size, the curves are shown for a representative set of individual mice (distributions are shown for all mice in Supplementary Information 4). *n* ≥ 3 mice per time point. **b**, Rescaled cumulative distribution of the logarithm of WT (left) and *Brca1;Trp53* (right) luminal confetti clone size, ln *n*, showing the probability of finding a clone with a size larger than $({\rm{l}}{\rm{n}}\,n-\mu )/\sigma$, where $\mu =\langle {\rm{l}}{\rm{n}}\,n\rangle$ denotes the average of the logarithm of clone size and ${\sigma }^{2}=\langle ({\rm{l}}{\rm{n}}\,n-\langle {\rm{l}}{\rm{n}}\,n\rangle {)}^{2}\rangle$ represents the variance. Points show data from **a**. Once rescaled, data from different time points collapse onto a single curve that fits well with the scaling function $(1/2){\rm{erfc}}(x/\sqrt{2})$ (cyan dashed line), consistent with a log-normal size dependence. For details of statistical significance tests, see Supplementary Information 4. **c**, Variance of the logarithm clone size, ${\sigma }^{2}(t)$, as a function of the inferred oestrous cycle number for luminal (black) and basal (blue) WT (left) and *Brca1;Trp53* (right) confetti clones. Points show data from individual mice and lines (dashed) show a fit to a linear growth characteristic, as predicted by a minimal model of clonal fate based on stochastic growth and regression (main text and Supplementary Information 4). **d**, Average of the logarithm clone size, *μ*(*t*), as a function of the inferred oestrous cycle number for luminal (black) and basal (blue) WT (left) and *Brca1;Trp53* (right) confetti clones. Points show data collected from individual mice and lines (dashed) show a fit to a linear growth characteristic, as predicted by a minimal model. See Supplementary Information 1 for sample sizes and statistics for **a**–**d**. Source Data

We therefore questioned whether other statistical features of the clone size distribution could provide insight into the underlying dynamics. On the basis of the huge variability of WT clone sizes (Figs. 2c and 3a and Extended Data Fig. 8c), we questioned whether the distribution of the logarithm of clone size, ln *n*, might show evidence of scaling. Notably, defining $C(w,t)$ as the probability of finding a clone with a size larger than *w* = ln *n*, we found that, when plotted as a function of $\left(w-\mu \left(t\right)\right)/\sigma (t)$, where $\mu (t)=\langle {\rm{l}}{\rm{n}}\,n\rangle$ denotes the average of the logarithm of clone size and ${\sigma }^{2}(t)=\langle ({\rm{l}}{\rm{n}}\,n-\langle {\rm{l}}{\rm{n}}\,n\rangle {)}^{2}\rangle$ represents its variance, the clone size data collapsed onto a single scaling curve (Fig. 3b, Extended Data Fig. 8d and Supplementary Information 4); that is, $C(w,t)=C((w-\mu (t))/\sigma (t))$, where the scaling function $C(x)$ is time independent (Supplementary Information 4). Further, inspection of the distribution showed that the scaling function fits well with a log-normal size dependence, with $C(x)=(1/2){\rm{e}}{\rm{r}}{\rm{f}}{\rm{c}}(x/\sqrt{2})$, where erfc denotes the complementary error function. These findings provide evidence of a statistical scaling behaviour of WT clone sizes distinct from that encountered in systems supported by local stochastic stem cell loss and replacement^44^, pointing to a different pattern of homeostatic turnover. Yet, the emergence of a simple and conserved pattern of WT clone size expansion, dependent only on the average and variance, $\mu (t)$ and ${{\sigma }}^{2}(t)$, suggested that the variability of WT clone sizes derived from cells conforming to a common statistical rule (Fig. 3c,d and Extended Data Fig. 8e,f).

## Oestrous cycle drives loss and replacement of MaSCs

**Fig. 4:**
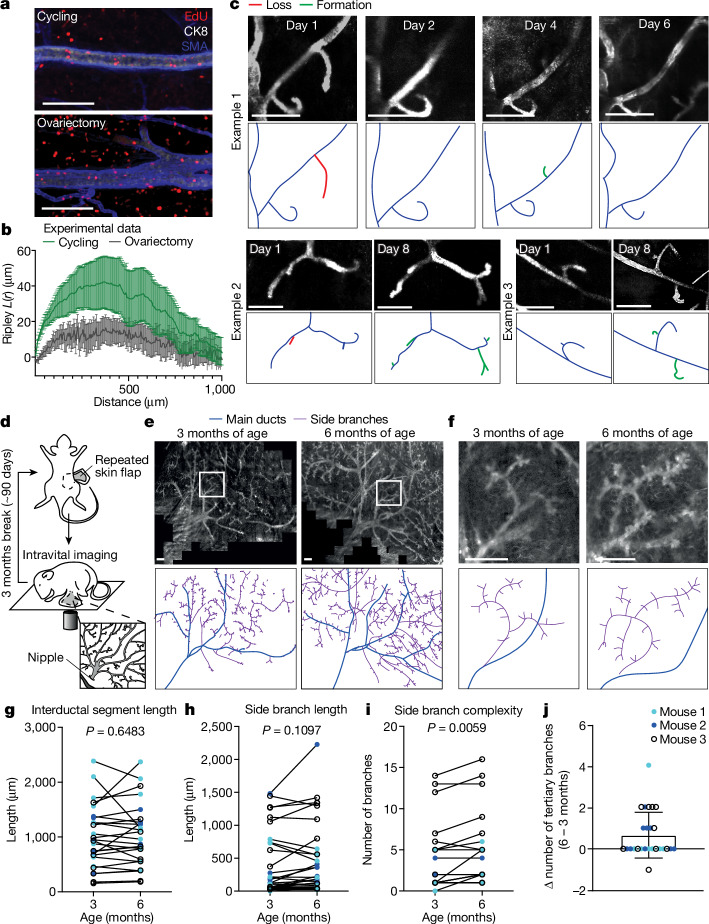
Clonal expansion beyond MaSC-descendant units by local remodelling. **a**, 3D views of mammary ducts showing EdU incorporation over 1 week in oestrous-cycling mice (top, three mice) and after ovariectomy (bottom, five mice), stained for CK8 and SMA. **b**, Ripley cluster analysis of EdU^+^ cell clusters along mammary ducts in cycling (green) and ovariectomized (black) mice. Data are mean ± s.e.m., five regions per mouse, three cycling mice, five ovariectomized mice. **c**, Branching dynamics in *KikGR* mice imaged through a mammary imaging window over 1 week. Representative examples of side branch expansion and regression are shown as indicated, *n* = 5 mice. **d**, Schematic of the repeated skin-flap procedure to visualize the mammary tree using intravital microscopy. **e**, Top panels show in vivo overviews of the fourth mammary gland of a *R26-mTmG* mouse at 3 (left) and 6 months of age (right) during oestrus. Bottom panels show outlines of the ductal tree with the main ducts in blue and side branches in red. Representative of four animals. **f**, Top panels show in vivo confocal images of the ductal area (red box in **b**) at 3 (left) and 6 months of age (right). Bottom panels show outlines with the main ducts in blue and side branches in red. **g**–**j**, Quantification of segment length of ducts (**g**), tertiary branch length (**h**) and tertiary branch complexity (**i**) at 3 and 6 months of age. **j**, Difference (Δ) in the number of tertiary branches between 6 and 3 months of age. Data derived from **i**. Colours indicate different mice, lines connect measurements of the same structures. Significance tested using a paired *t*-test, two-sided. See Supplementary Information 1 for more sample sizes, *P* values and statistics. Scale bars, 100 μm (**a**), 500 μm (**c**,**e**,**f**). Source Data

**Extended Data Fig. 9:**
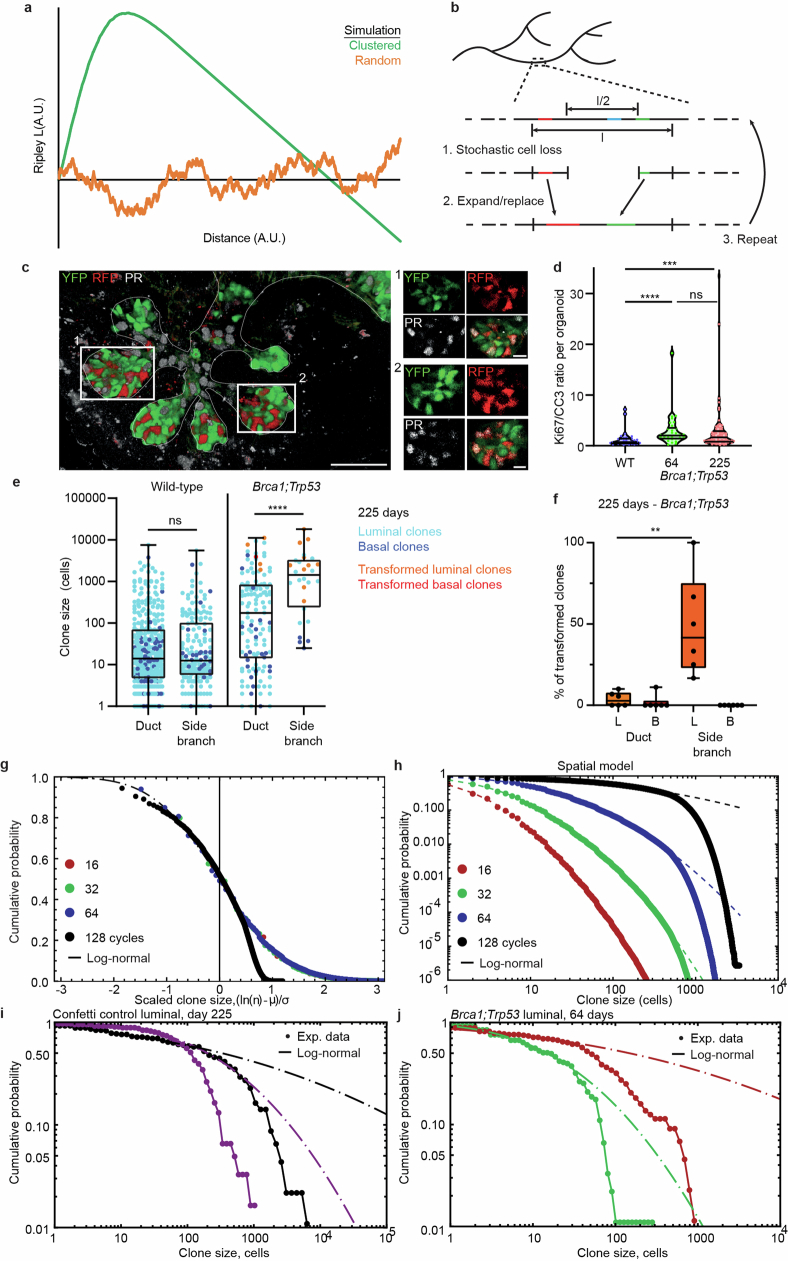
Fits and predictions of the phenomenological theory of the turnover of the mammary gland epithelium. **a**, Simulation of unclustered and clustered data analyzed with the Ripley’s K analysis leading to the Ripley’s L function. Two datasets were used for the analysis; 50 points from a uniform random distribution and 50 points from a normal distribution were generated for the clustered simulation, 100 random points from a uniform random distribution for the unclustered simulation. Details on the code and data can be found at https://github.com/BioImaging-NKI/qupath_ripley. **b**, Schematic depicting spatial model of ductal turnover (for details, see Supplementary Information 4). The mammary ductal epithelium is represented as a one-dimensional lattice. During the oestrous cycle, random non-overlapping domains of size *l* cells are turned over so that the central domain of *l*/2 cells are lost and replaced by the stochastic expansion of the 2 × *l*/4 neighboring sites. Through iterations of this process, clones are continuously lost, while others expand. Once clones extend beyond the size of the activated domain, *l*, their further expansion proceeds as a process of stochastic expansion and contraction on the clone boundary. **c**, 3D-rendering of confocal Z-stacks (overview image) and single Z-plane (zoom images) showing full labelling of two luminal lineages in the same part of the mammary gland after 550 days of lineage tracing; a PR^+^ clone labelling all PR^+^ luminal cells in this region (confetti RFP), and a PR^−^ clone labelling all PR^−^ in this part of the mammary gland (confetti YFP). PR^+^ luminal cells are shown in white, confetti YFP cells are shown in green. Scale bars represent 50 µm (overview image) and 10 µm (zoom images). Representative example of *n* = 3 mice. **d**, Quantification of ratio between Ki-67^+^ (proliferative) and cleaved caspase3^+^ (CC3) cells within organoids derived from wild-type (WT) or *Brca1;Trp53* confetti+ mammary epithelial cells, 64 and 225 days post-recombination. Each dot represents an organoid (*n* = 33 organoids for WT condition, *n* = 30 organoids for the *Brca1;Trp53* 64 days condition, and *n* = 74 organoids for the *Brca1;Trp53* 225 days condition. Violin plots depict distribution of data points, horizontal lines denote median, 1st and 3rd quartile. Significance was tested using a two-sided Mann Whitney Test, *** *P* < 0.0005, **** *P* < 0.0001, ns *P* = 0.1572. **e**, Quantification of luminal (cyan dots) and basal (blue dots) wild-type confetti clones (left) and *Brca1;Trp53* confetti clones (right) in the ducts and side branches, represented on a logarithmic scale. For each timepoint at least *n* = 6 glands from 3 mice were analyzed. Morphologically transformed clones are indicated in orange (luminal clones) and red (basal clones). Boxplots mark the 25th and 75th percentile, line indicates the median, and whiskers mark the minimum and maximum values. Significance was tested using a two-sided Mann-Whitney test, **** *P* < 0.0001. **f**, Transformed luminal (L, orange) and basal (B, red) clones in the ducts and side branches as percentage of the total number of luminal or basal clones respectively. Each dot indicates an individual mouse and boxplots mark the 25th and 75th percentile, line indicates the median, and whiskers mark the minimum and maximum values. Significance was tested using a two-sided Mann-Whitney test, ** *P* < 0.01. **g**, Cumulative distribution of the logarithm of clone size, ln *n*, obtained from the spatial cell-based model in (**b**) showing the probability of finding a clone with a size larger than $({\rm{l}}{\rm{n}}\,n-\mu )/\sigma$, where *μ* and ${\sigma }^{2}$ are obtained from a least-square fit of the data for *n* < *l*/2 to the log-normal size dependence, $(1/2){\rm{erfc}}(x/\sqrt{2})$ (dashed line) (cf. Fig. 3b and Extended Data Fig. 8d). Here, each lattice site is associated with a renewing MaSC with a total domain size of *l*=1000 lattice sites. The points show the results of stochastic simulation of the spatial model (averaged over an ensemble of 1000 realizations of the model on a periodic lattice of 10^6^ sites) for different numbers of oestrous cycles. In line with the quantitative analysis of the experimental data, the activation rate of domains is taken as 0.1 per oestrous cycle, with a loss probability set by the model of 0.5. For further details of the spatial model, see Supplementary Information 4. The code can be obtained from https://github.com/BenSimonsLab/Ciwinska_Nature_2024. Note that, at large time scales, the data departs from a log-normal size dependence. **h**, When plot on a log scale, the cumulative distribution of clone size shows the suppression at size scales in excess of the domain size *l*/2, a manifestation of the constraints imposed by the one-dimensional geometry of the ductal network. Points show the results of stochastic simulation and lines show the corresponding fits to the log-normal size dependence obtained from the fits in panel g. **i**, **j**, Cumulative distribution of luminal clone size for wild-type (**i**) and *Brca1;Trp53* (**j**) confetti clones for mice showing the largest effective oestrous cycle number from the 225 and 64 day time points, respectively (see Supplementary Information 4). Points show data and lines show the least-squares fits to a log-normal size dependence at small clone sizes. The respective colours are matched to the data shown in Supplementary Information 4. Note that, when plot on a log scale, the data reveals a departure from a log-normal size dependence, with a suppression at the largest clone sizes, mirroring the behavior of the spatial model (**h**). See Supplementary Information 1 for more sample sizes, *P* values and statistics for **e** and **f**. Source Data

Log-normal clonal distributions typically emerge from statistical processes in which the fate of proximate cells—amplification or loss—is positively correlated^45^. This could arise artefactually as the result of clone fragmentation or merger events^46^. However, the contiguity of WT clonal patches and sparsity of clonal labelling ruled against this possibility (Extended Data Fig. 4c,e). We therefore considered whether previous observations of cyclic expansion of the pool of MaSCs^19,21^ and regionally localized bouts of proliferation and cell death following the turnover of side branches during the oestrous cycle (Extended Data Fig. 6a–c)^9,19,21^ could locally correlate MaSC fate. To test this, we used an 5-ethynyl-2′-deoxyuridine (EdU) incorporation assay throughout at least one oestrous cycle. Even though EdU labelling showed heterogeneity along the ductal network (Fig. 4a), Ripley’s *L* function-based cluster analysis of the position of EdU^+^ cells provided evidence for a local correlation in proliferative activity (Fig. 4b and Extended Data Fig. 9a). This clustered proliferation decreased when we halted the oestrous cycle by ovariectomy (Fig. 4b).

To find further evidence for local tissue remodelling during the oestrous cycle, we visualized ductal remodelling by repeated rounds of intravital microscopy^47,48^. Indeed, over the course of a single cycle, we observed both the local formation and loss of side branches (Fig. 4c). Imaged over the course of 3 months (more than 12 cycles) using a repeated skin-flap method (Fig. 4d), the spatial organization of main ducts remained largely unaltered (depicted in blue, Fig. 4e–h). By contrast, the number of side branches increased over time, as well as their size and morphology, suggesting a small bias towards ‘bursts’ of localized growth over loss (as depicted in magenta, Fig. 4e,f,i,j). Together, these findings are in line with previous studies^9,19,21^ and support the presence of regional bursts of proliferation linked to oestrous cycle-mediated turnover of side branches.

## Modelling local MaSC fate reproduces clonal spread

To test quantitatively whether the observed bouts of local proliferation lead to the observed variation in sizes and temporal growth characteristics of WT clones, we derived a minimal model of MaSC fate in which individual MaSCs become active with some probability on side branch turnover during each oestrous cycle, after which they collectively expand or become altogether lost, with a relative probability that ensures long-term homeostasis (Extended Data Fig. 9b and Supplementary Information 4). As well as recapitulating the observed log-normal size dependence of WT clones, this minimal model predicted the dynamics of clone growth, including the emergence of a linear-like increase in the variance and average of the logarithm of clone size as a function of time (Fig. 3c,d), as well as the average clone size and the decay in the fraction of single-cell clones (Extended Data Fig. 8e,f). Note that, here, to account for large-scale mouse-to-mouse variation in the clonal dynamics, we used the coscaling of the average and variance of the logarithm of clone size to regress out a timescale, measured in terms of an effective oestrous cycle number, benchmarked against measurements obtained from previous studies (see Supplementary Information 4 for further details and fit parameters).

From a quantitative fit to the clone size data, we estimated that each MaSC supports just a few short-lived descendants on average, consistent with the pattern of clonal loss observed soon after induction (Fig. 2d). From a fit to the average growth characteristics, we estimated that each MaSC becomes active, contributing to the formation of a side branch, roughly once per ten oestrous cycles. Indeed, proliferation and apoptosis vary spatially during the oestrous cycle^9,19,21^ and the same cells are not in a proliferative state each cycle^14,15,49^. Owing to MaSC turnover, the model predicted that over a single cycle, 50% of ‘activated clones’ are lost while the surviving clones expand proportionately (that is, field clonalization), a result consistent with the observed formation and loss of side branches (Fig. 4c). With such a localized and cooperative pattern of turnover, it is plausible that MaSC fate would be highly correlated spatially (schematic in Extended Data Fig. 6a). Although neighbouring MaSCs labelled with different confetti colours were extremely rare owing to the sparse labelling, when such events did occur we found that their expansion was highly correlated, even when clones belonged to independent HR^+^ and HR^−^ sublineages (Extended Data Fig. 9c).

## Spread of mutant and WT clones follows similar rules

Having traced the origin of WT clone expansion and field clonalization during the oestrous cycle, we turned to consider the dynamics of mutant confetti clones, quantifying 1,745 *Brca1;Trp53* clones in 64 glands from 19 mice at time points from 14 to 225 days after labelling (Fig. 2c and Extended Data Fig. 5e,f). To focus on non-transformed or early transformed lesions, we excluded the palpable lesions (if present) from our analyses. This analysis showed that the spread of *Brca1;Trp53* confetti clones mirrored that of WT clones, showing the same conserved pattern of expansion with a log-normal size dependence signature (Fig. 3a,b, Extended Data Fig. 8c,d and Supplementary Information 4), and a corresponding linear-like increase in the average and variance of the logarithm of clone size (Fig. 3c,d and Supplementary Information 4). Moreover, the total number of surviving *Brca1;Trp53* confetti clones showed a similarly steep decrease in the first few months following induction (Fig. 2d). A fit to the linear growth characteristics showed that luminal *Brca1;Trp53* confetti clones experience a net spreading advantage over WT confetti clones, with a marginal increase in the degree of amplification during the oestrous cycle, suggesting a resistance to loss during regression (Supplementary Information 4), a result that we could confirm in vitro (Extended Data Fig. 9d).

We then investigated whether the spreading advantage of *Brca1;Trp53* confetti clones had a regional dependence, distinguishing between clones localized in the static main ducts and those in the more dynamic side branches. Focusing on the 225-day time point, for WT confetti clones, we found no regional dependence (Extended Data Fig. 9e). By contrast, although clone numbers were small, the distribution of *Brca1;Trp53* confetti clones in side branches showed a bias towards larger clone sizes characterized by a much narrower size distribution when compared to the main ducts (Extended Data Fig. 9e), which coincided with higher chance of transformation (Extended Data Fig. 9f). As *Brca1;Trp53* cells seem to be more resistant to loss (Extended Data Fig. 9d), mutant side branches generated during the oestrous cycle may be more durable than those formed by WT cells, enabling clones to spread more readily.

## Clonal spread is restrained by the ductal geometry

Within the framework of the minimal (non-spatial) model of clone growth, surviving WT and *Brca1;Trp53* clones are predicted to expand in size indefinitely at an exponential rate. Yet, such behaviour must become untenable in a tissue context. Therefore, to investigate how the tissue geometry might influence clone expansion, we developed a spatial model, representing the ductal epithelium as a one-dimensional ‘lattice-like’ ribbon of cells. By modelling the observed localized and cooperative loss and replacement of MaSCs during the turnover of side branches each oestrous cycle (Extended Data Figs. 6a and 9b), we could reproduce quantitatively the log-normal size distribution (Extended Data Fig. 9g and Supplementary Information 4). However, at long times, the lattice model predicted that the expansion of clones should become suppressed at the length scale of the remodelled regions, and the clone size distribution cross over from log-normal to a narrower Gaussian-like dependence, consistent with the dynamics expected for neutral cell competition between neighbouring cells in one dimension (Extended Data Fig. 9h). Consistently, applied to the experimental data, departures from the log-normal dependence could be observed for both WT and *Brca1;Trp53* confetti clones when clone sizes spanned hundreds of cells or more (Extended Data Fig. 9i,j). Together, these results indicate that the one-dimensional ductal organization provides a geometrical constraint that ultimately limits the range expansion of clones, limiting field clonalization and cancerization.

## Pregnancy does not increase spread of mutant clones

**Extended Data Fig. 10:**
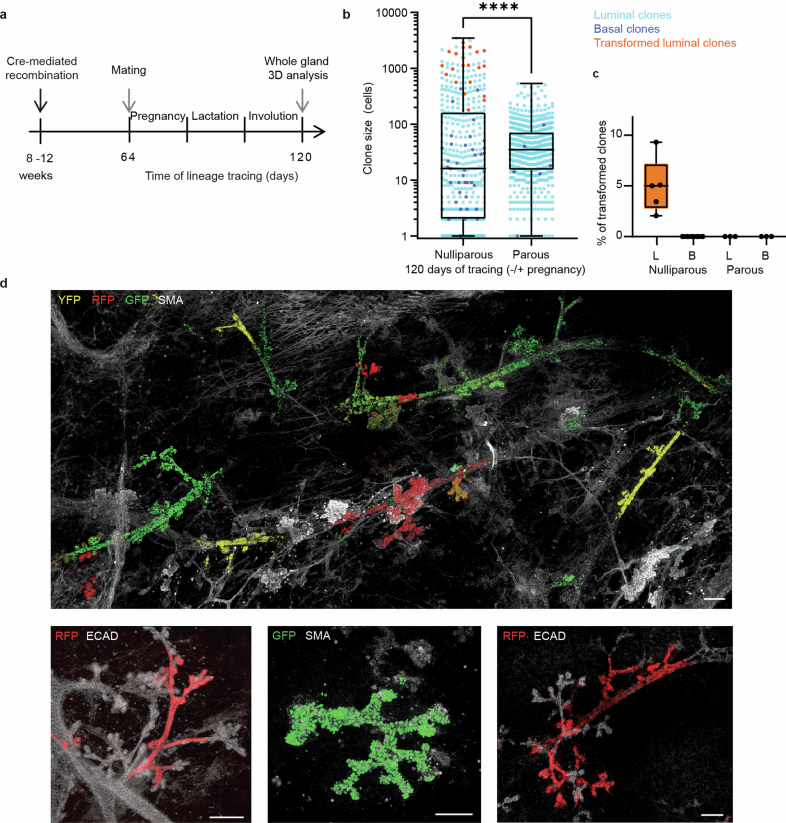
Pregnancy and lactation do not increase the spread of *Brca1;Trp53* mutant clones. **a**, Schematic depicting the experimental timeline of the pregnancy and lactation experiments in induced *Brca1;Trp53* confetti glands. **b**, Quantification of *Brca1;Trp53* confetti clone sizes in nulliparous (left) and parous (right) glands, 120 days after recombination and lineage tracing initiation represented on a logarithmic scale. For each timepoint, at least *n* = 3 glands from 3 different mice were analyzed. Boxplots mark the 25th and 75th percentile, line indicates the median, and whiskers mark the minimum and maximum values. Significance was tested using a two-sided Mann-Whitney test, **** *P* < 0.0001. **c**, Transformed luminal (L, orange) and basal (B, red) clones as a percentage of the total number of luminal or basal clones respectively. Each dot indicates an individual mouse and boxplots mark the 25th and 75th percentile, line indicates the median, and whiskers mark the minimum and maximum values. *n* = 5 mice (nulliparous) and *n* = 3 mice (parous). **d**, Representative whole-mount confocal images of *Brca1;Trp53* confetti clones in parous glands (one pregnancy-involution cycle), 120 days after recombination. Luminal cells are labelled with E-cadherin (ECAD), basal cells are labelled with alpha-smooth muscle actin (SMA). Images depict 3D-rendering of Z-stacks. Scale bars represent 100 µm. Representative image of *n* = 3 biological repeats (mice). See Supplementary Information 1 for more sample sizes, *P* values and statistics for **b, c**. Source Data

Next, we questioned how the fate of mutant clones is perturbed during pregnancy, which is associated with massive tissue remodelling. Following induction of *Brca1;Trp53* mutant clones in 8–12-week-old mice, at day 64 post-induction, mice went through a round of pregnancy, lactation and involution (Extended Data Fig. 10a). Parous *Brca1;Trp53* mice were analysed at 120 days post-induction and compared to their nulliparous counterparts. Although pregnancy-driven reshaping of the ductal network leads to massive expansion and involution of localized areas^12^, this did not lead to significant changes in clone sizes in the parous versus nulliparous mice (Extended Data Fig. 10b). Further inspection of mutant clone sizes in parous and nulliparous mice revealed a striking shift in the clone size distribution, with the former having a relatively low number of large clones, potentially the result of skipping several oestrous cycles (Extended Data Fig. 10b). In contrast to the nulliparous mice, no transformed clones were observed at 120 days post-induction in the mammary glands of parous *Brca1;Trp53* mice (Extended Data Fig. 10b–d), consistent with the conclusion that clonal expansions make ducts more susceptible to transformation. It also echoes the results of previous studies demonstrating the protective role of early parity in breast cancer^50^. In line with this reasoning, in nulliparous glands mutant clones localized at the dynamic side branches showed a bias towards large sizes and have a proportionately higher propensity to transform (Extended Data Fig. 9e,f). Such behaviour mirrors that found in humans, in which transformation is also thought to occur predominantly in side branches—the terminal ductal lobular units^51,52^.

## Abolishing mutant spread reduces transformation

**Fig. 5:**
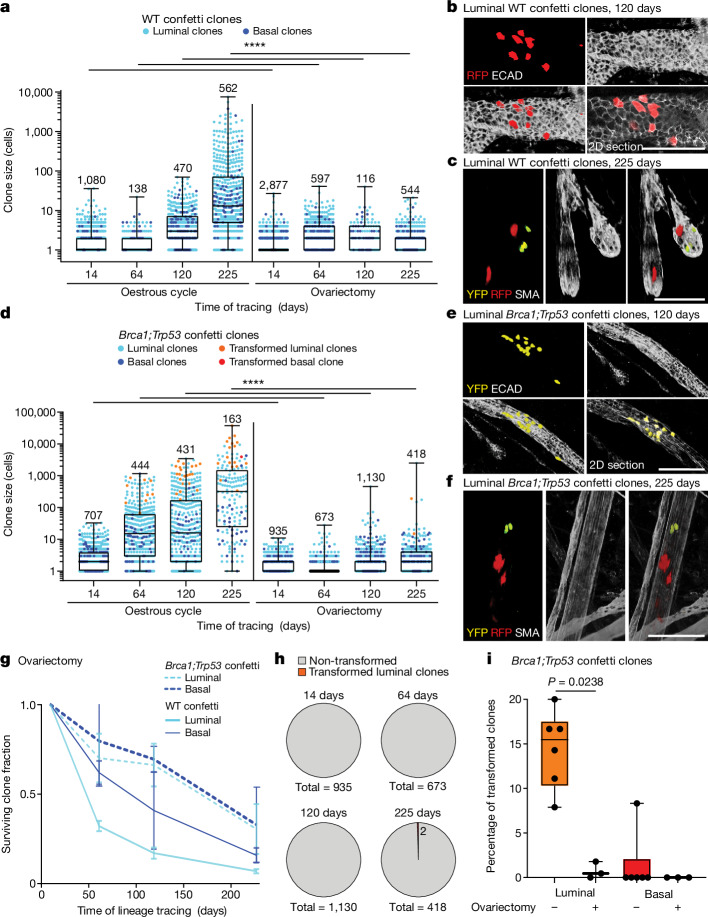
Field clonalization and cancerization are abolished in the absence of the oestrous cycle. **a**, Luminal (cyan) and basal (blue) WT confetti clone sizes in the homeostatic gland (left, same as Fig. 2c), and after ovariectomy (right). **b**,**c**, Representative whole-mount confocal images of luminal WT confetti clones 120 days (**b**) and 225 days (**c**) after recombination in ovariectomized mice (*n* ≥ 3 mice per condition). Luminal cells are labelled with ECAD (**b**), basal cells are labelled with SMA (**c**). Images depict 3D rendering of *Z*-stacks, unless otherwise indicated. **d**, Luminal (cyan) and basal (blue) *Brca1;Trp53* confetti clone sizes in the oestrous-cycling condition (left, same as Fig. 2c), and after ovariectomy (right). **e**,**f**, Representative whole-mount confocal images of luminal *Brca1;Trp53* confetti clones 120 (**e**) and 225 days (**f**) after recombination in ovariectomized mice (*n* ≥ 3 mice per condition). Luminal cells labelled with ECAD (**e**), basal cells labelled with SMA (**f**). Images depict 3D rendering of *Z*-stacks, unless otherwise indicated. **g**, Surviving clone fraction in ovariectomized mice as function of time normalized to the average number of confetti^+^ cells at 14 days. Error bars represent mean ± s.e.m. **h**, Non-transformed luminal or basal clones (grey) and transformed luminal clones (orange) in the ovariectomized *Brca1;Trp53* confetti mouse model. **i**, Transformed luminal (L, orange) and basal (B, red) clones as percentages of the total number of luminal or basal clones, respectively, in the cycling and ovariectomized conditions. Each dot indicates an individual mouse. **a**,**d**,**h**, Clone numbers are indicated. **a**,**d**,**i**, Boxplots mark 25th and 75th percentile, the line indicates median and the whiskers mark minimum and maximum values. Significance was tested using a two-sided Mann–Whitney test, ****P < 0.0001. See Supplementary Information 1 for more sample sizes, *P* values and statistics. Scale bars, 100 µm. Source Data

**Extended Data Fig. 11:**
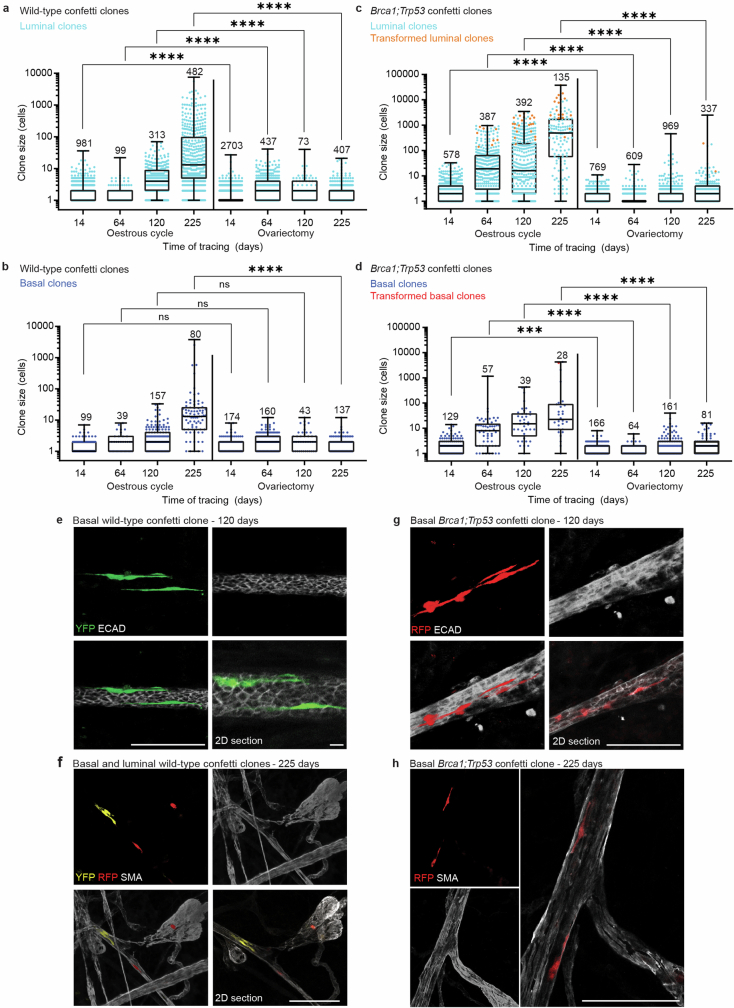
Ovariectomy abolishes field clonalization and cancerization of basal and luminal cell clones. **a**, **b**, Clone size quantification of luminal (**a**) and basal (**b**) wild-type confetti clones in the homeostatic gland (left), and after ovariectomy (right) represented on a logarithmic scale. Ovariectomy abolishes clonal expansion and field clonalization. Same data as Fig. 5a, but now with basal and luminal clones presented in separate graphs. For each timepoint at least *n* = 6 glands from 3 different mice were analyzed. Analyzed number of clones for each timepoint are indicated in the graphs. Boxplots mark the 25th and 75th percentile, line indicates the median, and whiskers mark the minimum and maximum values. Significance was tested using a two-sided Mann-Whitney test, *** *P* < 0.001, **** *P* < 0.0001. **c**, **d**, Clone size quantification of luminal (**c**) and basal (**d**) *Brca1;Trp53* confetti clones in the presence of oestrous cycling (left) and after ovariectomy (right) represented on a logarithmic scale. Ovariectomy abolishes clonal expansion and field cancerization. Same data as Fig. 5d, but now with basal and luminal clones presented in separate graphs. For each timepoint at least *n* = 6 glands from 3 different mice were analyzed. Analyzed number of clones for each timepoint are indicated in the graphs. Boxplots mark the 25th and 75th percentile, line indicates the median, and whiskers mark the minimum and maximum values. Significance was tested using a two-sided Mann-Whitney test, *** *P* < 0.001, **** *P* < 0.0001. **e**, **f**, Representative whole-mount confocal images of basal wild-type confetti clones 120 days (**e**) and basal and luminal wild-type confetti clones 225 days (**f**) after recombination in ovariectomized condition. Luminal cells are labelled with E-cadherin (ECAD) (**e**), basal cells are labelled with alpha-smooth muscle actin (SMA) (**f**). Images depict 3D-rendering of Z-stacks, unless otherwise indicated. Scale bars represent 100 µm, except for the scale bar in 2D section (**e**) which represents 10 µm. Representative images of *n* = 3 biological repeats (mice). **g**, **h**, Representative whole-mount confocal images of basal *Brca1;Trp53* confetti clones 120 days (**g**) and 225 days (**h**) after recombination in ovariectomized condition. Luminal cells are labelled with E-cadherin (ECAD) (**g**), basal cells are labelled with alpha-smooth muscle actin (SMA) (**h**). Images depict 3D-rendering of Z-stacks, unless otherwise indicated. Scale bars represent 100 µm. Representative images of *n* = 3 biological repeats (mice). See Supplementary Information 1 for more sample sizes, *P* values and statistics for **a-d**. Source Data

**Extended Data Fig. 12:**
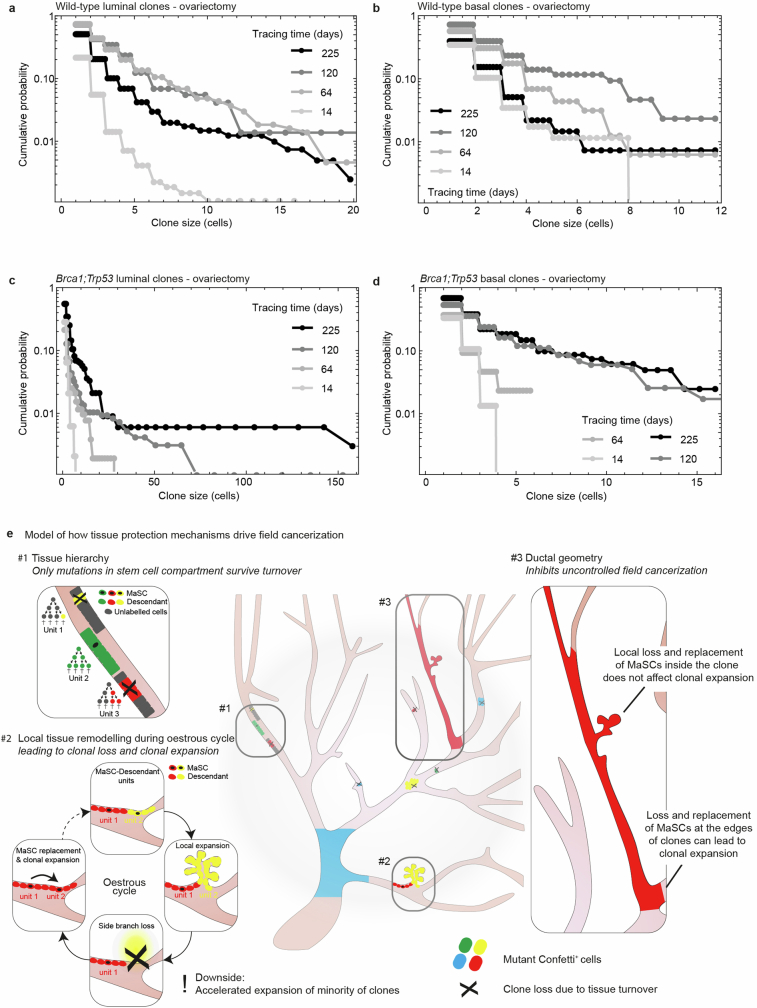
Tissue protection mechanisms against field cancerization in the mammary gland. **a**, **b**, Cumulative distribution of luminal (**a**) and basal (**b**) clone size of ovariectomized wild-type confetti mice showing the probability (log scale) of finding a clone larger than the given size across a range of time points. **c**, **d**, Cumulative distribution of luminal (**c**) and basal (**d**) clone size of ovariectomized *Brca1;Trp53* confetti mice showing the probability (log scale) of finding a clone larger than the given size across a range of time point. **e**, Model depicting how tissue protection mechanisms drive field cancerization in the mammary gland. The mammary ductal epithelium confers several layers of protection against field cancerization by mutant cells. Protection mechanism #1: The ductal epithelial network is supported by a short, lineage-restricted MaSC-descendant cell hierarchy. As a result, the majority of mutant cells will be lost through homeostatic tissue turnover, and only a few mutations rooted in the stem cell compartment can survive in the medium term. Protection mechanism #2: Local stem cell loss and replacement driven by the oestrous cycle leads to large-scale elimination of the majority of mutant stem cell clones over time. This large-scale clonal loss occurs at the expense of an accelerated (exponential-like) expansion of the minority of clones that survive, allowing them to colonize large areas of the epithelium. Protection mechanism #3: Once clones extend beyond the size of regions activated during the oestrous cycle, their expansion becomes limited by the one-dimensional geometry of the ducts, a phenomenon that is particularly effective in restricting mutant clone expansion. Source Data

To further test the relation between clone size and transformation susceptibility, we blocked experimentally the expansion of *Brca1;Trp53* confetti clones by performing an ovariectomy. If rapid amplification of clone sizes is associated with oestrous cycle-driven side branch turnover, we reasoned that, in its absence, the susceptibility for transformation should be largely abolished^53^. Indeed, at later time points (120 and 225 days), when large-scale expansion of clones becomes evident in non-ovariectomized glands, no extensive spread of WT and mutant clones was observed in ovariectomized glands (Fig. 5a–f and Extended Data Figs. 11 and 12a–d). By contrast, the surviving clone fraction still decreased (Fig. 5g), consistent with the turnover of cells within the MaSC-descendant units; although for *Brca1;Trp53* clones there was a slower loss rate as compared to non-ovariectomized mice (*P* < 0.05, Supplementary Information 2). Notably, there was a trend towards a faster loss of WT over *Brca1;Trp53* confetti clones, consistent with a survival advantage of the former over WT neighbours (Fig. 5g and Supplementary Information 2). The limited spread of the *Brca1;Trp53* confetti clones was accompanied by a near complete loss of susceptibility for transformation (Fig. 5h,i).

## Discussion

The abundance of mutant cells in human breast tissue from healthy individuals, and its potential relevance for the initiation and recurrence of breast cancer, have long been recognized^1,7,8^. Here, by tracing the dynamics of WT and *Brca1;Trp53* confetti clones in mouse tissue, we have addressed the cellular basis of field cancerization and the sick lobe theory. In contrast to prevailing models of the mouse mammary gland^29,42,54,55^ and human breast tissue^56,57^, our results indicate that MaSCs are distributed uniformly along the ductal tree, a finding resonant with a previous study of human breast tissue^34^. Following the induction of WT or mutant clones, only those clones that are ‘rooted’ in the MaSC compartment survive over the short term, with their spread limited to their descendant units. In the absence of the oestrous cycle, the spread of mutant clones beyond these units remains limited, even over the long term. However, during stages of the oestrous cycle, ovarian hormones act on HR^+^ luminal cells, triggering remodelling of side branches leading to localized proliferation and apoptosis of HR^+^, HR^−^ luminal and basal cells^14,15,20,30^. These bouts of growth and regression drive the local and coordinated expansion and loss of MaSCs (regardless of their HR expression), leading to the elimination of most mutant clones, including those bearing oncogenic driver mutations, whereas those that by chance survive expand exponentially (Extended Data Fig. 12e). Therefore, any clone, regardless of its size, HR status or proliferation capacity, can by chance either increase or decrease in size at any given oestrous cycle. This explains why the spread of clones negative for the HR (for example, basal or *Brca1;Trp53* confetti clones) still depends on the oestrous cycle. This process of field clonalization enables entire ductal subtrees to become predisposed to the development of aberrant ductal lesions and transformation, a behaviour resonant with the abundance of signature genomic aberrations observed in both large transformed and non-transformed *Brca1;Trp53* confetti clones. Moreover, consistent with these findings, clinical observations show that the risk of tumorigenesis increases with the number of menstrual cycles, the human variant of the oestrous cycle. In particular, the risk of breast cancer correlates with the age of entry into menopause, with increasing age indicating an enhanced number of cycles^58–60^. Yet, it is essential to emphasize that the dependence of clonal expansion on the oestrous cycle is different at later stages of tumour development. At the stage when *Brca1;Trp53* tumours grow invasively, clonal expansion is no longer affected by the tissue remodelling that accompanies the oestrous cycle or limited by the one-dimensional architecture of the ductal network, and thus tumour growth is unaffected by ovariectomy^53^.

## Methods

### Mice

All mice used for experiments were adult females from a mixed background, housed under standard laboratory conditions and receiving food and water ad libitum. All experiments were performed in accordance with the guidelines of the Animal Welfare Committees of the Netherlands Cancer Institute and KU Leuven. Sample size was determined using a resource equation approach, mice were randomly assigned to experimental groups and blinding was performed during data analysis. *R26R-Confetti*^*het*^ (JAX stock no. 013731)^61,62^*; R26-CreERT2*^*het*^ (JAX stock no. 008463)^63^ mice were injected intraperitoneally with tamoxifen (Sigma-Aldrich), diluted in sunflower oil, to activate Cre recombinase. To achieve clonal density labelling (fewer than one MaSC per duct on average), *R26R-Confetti*^*het*^*;R26-CreERT2*^*het*^ mice were injected with 1 mg of tamoxifen per 25 g of body weight between 10 and 15 weeks of age. Ovariectomies were performed between 10 and 15 weeks of age, at least 7 days before lineage tracing initiation. The third, fourth and fifth mammary glands of *R26R-Confetti;Brca1*^*fl/fl*^*;Trp53*^*fl/fl*^ (refs. ^31,64^) or *R26R-Confetti* mice were intraductally injected with recombinant TAT-Cre protein (20 units per gland diluted in 20 µl of PBS, Sigma-Aldrich) between 10 and 15 weeks of age. This TAT-Cre injection resulted in roughly one labelled cell for every 100–200 cells (Extended Data Fig. 4e). As the confetti construct comprises four distinct colours, there is, on average, one cell labelled with a confetti colour per 400 cells. Considering that a MaSC-progeny unit consists of roughly five to ten cells, a single confetti-labelled cell is induced in one out of 40–80 units. Over time, many clones become extinct, leading to a dilution in the number of clones and making collisions even less likely. For each of the experiments, mice were analysed at different time points after lineage tracing initiation as indicated in Fig. 1b. The injected mammary glands of *R26R-Confetti;Brca1*^*fl/fl*^*;Trp53*^*fl/fl*^ mice at the latest time point (225 days) were analysed when one of the injected glands developed a palpable tumour of at least 5 × 5 mm, which was between 200 and 250 days after recombination. Tumour sizes did not exceed 1,500 mm^3^ in accordance with the guidelines of the Animal Welfare Committee of the Royal Netherlands Academy of Arts and Sciences, the Netherlands Cancer Institute and KU Leuven. Samples were randomly allocated to the experimental groups, sample size was not determined a priori and investigators were not blinded to experimental conditions, except where indicated. For clonal analysis of the *R26R-Confetti;Brca1*^*fl/fl*^*;Trp53*^*fl/fl*^ model, we analysed *n* = 4 mice (14 days), *n* = 4 mice (64 days), *n* = 5 mice (120 days) and *n* = 6 mice (225 days). For clonal analysis of the *R26R-Confetti* model, we analysed *n* = 3 mice (14 days), *n* = 3 mice (64 days), *n* = 5 mice (120 days) and *n* = 6 mice (225 days). Clonal analysis of the ovariectomized *R26R-Confetti;Brca1*^*fl/fl*^*;Trp53*^*fl/fl*^ and *R26R-Confetti*^*het*^ models was performed on at least *n* = 3 mice per time point. Adult *CAG;;KikGR* female mice^65^ (RIKEN no. CLSTCDB0201T-117830853340) were used to visualize the short-term dynamics of the mammary gland by repeated imaging through a mammary imaging window as described below. *R26-mTmG* female mice (JAX no. 007676)^66^ were used to visualize the long-term stability of the mammary gland through a repeated skin-flap procedure as described below.

### Mammary imaging window implantation, repeated skin-flap procedure and intravital imaging

Mice were anaesthetized using isoflurane (Isovet) inhalation (1.5/2% isoflurane/air mixture). The fourth mammary gland of adult *CAG;;KikGR* female mice was imaged repeatedly through a mammary imaging window as previously described^47,48^. The fourth mammary gland of adult *R26-mTmG* mice was imaged repeatedly with a skin flap as previously described^47^. To visualize the mammary gland, mice were placed in a facemask within a custom designed imaging box. Isoflurane was introduced through the facemask and ventilated by an outlet on the other side of the box. The imaging box and microscope were kept at 34 °C by a climate chamber surrounding the entire stage of the microscope including the objectives. Imaging was performed on an inverted Leica SP8 Dive system (Leica Microsystems) equipped with four tuneable hybrid detectors, a MaiTai eHP DeepSee laser (Spectra-Physics) and an InSight X3 laser (Spectra-Physics) using the Leica Application Suite X (LAS X) software. All images were collected at 8 bit and acquired with a ×25 water immersion objective with a free working distance of 2.40 mm (HC FLUOTAR L ×25/0.95W VISIR 0.17). For the *CAG;;KikGR* model, Kikume Green was excited at 960 nm and detected at 490–550 nm. Each imaging session, all visible ducts through the imaging window were imaged using a tiled *z* scan with ×1–2 zoom and a *z*-step size of 5–10 μm. For the skin-flap imaging, TdTomato was excited at 1,040 nm and detected at 540–730 nm. All visible ducts were imaged together in one tile *z* scan with a ×0.75 zoom, a *z*-step size of 10–20 μm. These parameters allowed to scan large regions of up to 2 cm^2^ in less than 3 h. At the end of the first skin-flap imaging session, the skin was closed with a continuous, non-resorbable suture. After 3 months, the skin flap was re-opened for the second imaging session, and the same imaging fields were retraced using the nipple and collagen I structures of the first imaging session as landmarks.

### Staging of the mice

To determine the oestrous cycle stage of the mice, a vaginal swab was collected as described^67^. In short, the vagina was flushed using a plastic pipette filled with 50 µl PBS, and the liquid was transferred to a dry glass slide. After air drying, the slide was stained with Crystal Violet and the cell cytology was examined using a light microscope.

### Clone isolation and CNA sequencing

Here, 225 days after Cre mediated recombination, fourth mammary glands were extracted, fixed overnight in 1% paraformaldehyde (PFA), incubated in sucrose overnight and stored in optimal cutting temperature (OCT) at −80 °C. For microdissection of individual clones, OCT blocks were thawed at room temperature in the dark for 30 min and mammary glands removed from OCT and washed in 50 ml of PBS on ice. Mammary glands were dissected under a benchtop fluorescent macroscope (Zeiss) using Dumont forceps and fine scissors, using the clone morphology to distinguish between transformed and untransformed clones. Each dissected clone was washed in 1 ml of PBS on ice for 2–5 h (until the end of the dissection procedure). Per mammary gland, one piece of non-fluorescent tissue from the inguinal lymph node was dissected as internal sequencing control. After washing, pieces were lysed in 70 µl of Arcturus lysis buffer following the instructions of the ThermoFisher Scientific Arcturus PicoPure kit KIT0103. Lysis was carried out in a PCR cycler for 18 h at 65 °C followed by 30 min at 75 °C and holding at 4 °C. Samples were then purified using the Roche FFPE DNA extraction kit 06650767001 50-588-384 following the manufacturer’s instructions with elution in 25 µl of PCR grade water. For DNA sequencing, library preparation was carried out with a KAPA Hyper kit (Roche; KK8504) according to the manufacturer protocol with four PCR cycles, before the samples were sequenced by low-coverage whole genome sequencing. The copy number alteration (CNA) analysis was conducted in R, using QDNAseq with 50 kb bins and the mm10 mouse reference genome. This methodology yielded copy number values from both normal and transformed clones, along with an internal control sample. Normalization was achieved by first converting copy number values to log_2_, then subtracting the internal control sample’s values from those of the normal and transformed tumours. We averaged these adjusted copy number values for each replicate across both clone types (13 early normal clones and 13 early transformed clones). Data visualization was executed using the ggplot2 package in R, with specific emphasis on certain chromosomes. Regarding the late-stage tumours published in ref. ^40^, copy number profiling data corresponding to ten *Wap-Cre;Brca1*^*fl*/*fl*^*;Trp53*^*fl/fl*^ (WB1P) female mice harbouring a WB1P late-stage mammary tumour, along with internal control samples (spleen) was used. The CNA sequence analysis included the use of cutadapt for adaptor sequence removal and BWA for sequence alignment (using bwa aln, bwa mem) to the mm10 mouse genome. This procedure mirrored the earlier steps up to plotting with ggplot2, repeated for ten WB1P replicates.

### Quantification of the distribution of proliferation

Oestrous-cycling mice and mice that had undergone ovariectomy with a 2-week recovery period (all above 8 weeks of age) received 0.5 mg ml^−1^ EdU in drinking water (refreshed every second day) for 1 week. 3D imaging was performed on three cycling mice and five ovariectomized mice. Per mouse, one-quarter of the mammary gland was taken for subsequent analysis. Samples were fixed in 4% PFA overnight and stained using the FLASH protocol with FLASH Reagent 2 (ref. ^68^). Before adding the primary antibodies, samples were stained for EdU as follows. Tissues were incubated in 5 ml of 3% bovine serum albumin for 1 h, followed by three washes in PBS for 20 min each. EdU was detected with an Alexa-647 azide. The reaction cocktail for EdU fluorescent labelling was prepared according to the manufacturer’s guidelines using the Click-It EdU imaging kit (ThermoFisher Scientific). Per gland, 0.5 ml of reaction cocktail was added for incubation for 4 h at room temperature with gentle agitation on a nutator. The cocktail was removed, samples washed once in 3% bovine serum albumin in PBS for 20 min, followed by three washes in FLASH blocking buffer for 20 min each. Subsequently, samples were stained with primary and secondary antibodies overnight each. Primary antibodies used were KRT8 (rat, Troma-I, Merck Millipore, 1:800) and αSMA (mouse IgG2a, clone 1A4, ThermoFisher Scientific, 1:600). Secondary antibodies used were donkey antirat Alexa-488 and donkey antimouse Alexa546 (ThermoFisher Scientific, catalogue nos. A21208 and A10036, respectively, 1:400), combined with Hoechst 33342 for nucleus detection. Samples were imaged on an Andor Dragonfly spinning disc system, installed on an inverted Leica DMI8 microscope with an Andor Zyla 4+sCMOS camera using a ×10, 0.45 NA Fluo objective (Leica). Imaging was carried out with a 40 μm disc using 405 nm excitation, a 561 nm optically pumped semiconductor laser and 637 nm diode lasers. Images were visualized with Imaris Viewer using gamma correction, ortho slicers and cutting planes to depict deeper tissue layers. For each mammary gland, distribution of proliferation was quantified in five regions. Ripley analysis using QuPath^69^ was performed with a custom-made script (available at https://github.com/BioImaging-NKI/qupath_ripley). The image was opened in QuPath and a freehand line was drawn by hand to outline the duct for analysis. A multipoint annotation was drawn by hand to mark the positions of proliferating cells along the duct. The script calculated Ripleys K function and normalized it to an unclustered distribution resulting in Ripley’s L function. Data were plotted in GraphPad Prism v.10. For simulations, we have generated clustered and unclustered data in Python.

### Whole-mount immunofluorescence staining of mammary glands

The third, fourth and fifth mammary glands were dissected and incubated in a mixture of collagenase I (1 mg ml^−1^, Roche Diagnostics) and hyaluronidase (50 μg ml^−1^, Sigma-Aldrich) at 37 °C for optical clearance, fixed in periodate–lysine–PFA buffer (1% PFA; Electron Microscopy Science), 0.01 M sodium periodate, 0.075 M l-lysine and 0.0375 M P-buffer (0.081 M Na_2_HPO_4_ and 0.019 M NaH_2_PO_4_; pH 7.4) for 2 h at room temperature, and incubated for at least 3 h in blocking buffer containing 1% bovine serum albumin (Roche Diagnostics), 5% normal goat serum (Monosan) and 0.8% Triton X-100 (Sigma-Aldrich) in PBS. Primary antibodies were diluted in blocking buffer and incubated overnight at room temperature. Secondary antibodies diluted in blocking buffer were incubated for at least 6 h. Nuclei were stained with 4,6-diamidino-2-phenylindole (DAPI) (0.1 μg ml^−1^; Sigma-Aldrich) in PBS. Glands were washed with PBS and mounted on a microscopy slide with Vectashield hard set (H-1400, Vector Laboratories). Primary antibodies used were: anti-KRT14 (rabbit, Covance, PRB155P, 1:700), anti-ECAD (rat, eBioscience, 14-3249-82, 1:700), anti-oestrogen receptor (rabbit, no. 13258, Cell Signaling, 1:100), anti-progesterone receptor (rabbit, Clone SP2, MA5-14505, ThermoFisher Scientific, 1:200) and anti-SMA (mouse IgG2a, clone 1A4, Sigma-Aldrich, 1:600). Alexa Fluor 647 and Alexa Fluor 488 Phalloidin were used 1:500 (A-22287 and A-12379, ThermoFisher Scientific) and incubated together with the secondary antibodies. Secondary antibodies used were: goat antirabbit, goat antirat or goat antimouse IgG2a, all conjugated to Alexa-647 (ThermoFisher Scientific, catalogue nos. A21245, A21247 and A21241, respectively, 1:400).

### Whole-mount imaging of mammary glands

Imaging of whole-mount mammary glands was performed using an inverted Leica TCS SP8 confocal microscope, equipped with a 405 nm laser, an argon laser, a diode-pumped solid-state laser 561 nm laser and a HeNe 633 nm laser. Different fluorophores were excited as follows: DAPI at 405 nm, cyan fluorescent protein (CFP) at 458 nm, green fluorescent protein (GFP) at 488 nm, yellow fluorescent protein (YFP) at 514 nm, red fluorescent protein (RFP) at 561 nm and Alexa-647 at 633 nm. DAPI was collected at 440–470 nm, CFP at 470–485 nm, GFP at 495–510 nm, YFP at 540–570 nm, RFP at 610–640 nm and Alexa-647 at 650–700 nm. All images were acquired with a ×20 (HCX IRAPO N.A. 0.70 WD 0.5 mm) dry objective using a *Z*-step size of 1–5 μm (total *Z*-stack around 200 μm). 3D overview tile scans of the mammary glands were acquired by scanning large tile-scan areas (*xyz*). Next, detailed images were obtained of the individual clones. All images were stitched and processed in the true 3D real-time Rendering LAS X 3D Visualization module (Leica Microsystems) and further processed using ImageJ software (https://imagej.nih.gov/ij/).

### Clonal analysis on whole-mount glands

Three-dimensional tile-scan images of whole-mount and fully intact mammary glands were used to manually reconstruct the ductal network by outlining the ducts based on the labelling by ECAD, SMA or KRT14 (between 400 and 600 tiles, ×10 objective, *Z*-step size of 5–10 µm). After localization of the confetti clones in these 3D overview scans, each clone was imaged in detail with a ×25 water objective using confocal imaging by taking a *Z*-stack with step size between 1 and 3 µm. On the basis of the overlap with the luminal- or basal-cell-specific labelling and cellular morphology (that is, a cuboidal shape for luminal cells and an elongated shape for basal cell), the labelled confetti cells were identified and annotated in the schematic outline of the mammary tree, including information on their confetti colour (GFP, green; YFP, yellow; RFP, red and CFP, cyan) and their identity: that is, luminal or basal. Regions in which, for technical reasons, the gland could not be visualized well were omitted from analysis (in three out of 160 glands). Clone sizes, referring to the number of cells within each clone, were determined through manual visual inspection of tissue samples, with the quantification performed by eye using detailed *Z*-stack images and 3D rendering of each individual clone. Using custom-made.NET software (available on request from J.v.R.), the coordinates of the branch points, and the position of the labelled cells in ducts and in ductal ends were scored. To calculate the surviving clone fraction, the total number of clones was determined for each of the indicated lineage tracing time points by analysing the entire mammary gland in three dimensions using our whole-gland imaging approach (*n* = 3 glands per time point of two individual mice). Next, the average numbers of clones identified at 64, 120 and 225 days after lineage tracing initiation were divided by the average number of clones identified 14 days after lineage tracing initiation resulting in the surviving clone fraction as depicted in Figs. 2d and 5g.

### Mammary epithelial cell sorting and real-time qPCR

The third, fourth and fifth mammary glands of *R26R-Confetti;Brca1*^*fl/fl*^*;Trp53*^*fl/fl*^ mice were intraductally injected with recombinant TAT-Cre protein (20 units per gland diluted in 20 µl PBS, produced in-house) between 10 and 13 weeks of age. Then 120 to 180 days after injection, mammary glands were harvested, minced and digested at 37 °C for 30 min in a mixture of collagenase A (2 mg ml^−1^, Roche Diagnostics), hyaluronidase (300 μg ml^−1^, Sigma-Aldrich) and DNase (1 mg ml^−1^) in DMEM/F12 (Gibco). After 10 min incubation with TripLE (Gibco) at 37 °C cells were strained through a 100 μm cell strainer (Fisher Scientific) to obtain single cells. Cells were spun down for 10 min at 550 RCF (relative centrifugal force) at 4 °C followed by blocking for 15 min on ice in 5 mM EDTA/PBS with 2% sterile filtered normal goat serum (Gibco). CD45-Alexa-647 (clone 30-F11, 03123, Biolegend, 1:200) and EpCAM-APC/Cy7 (clone G8.8, 118218, Biolegend, 1:200) were diluted in 5 mM EDTA/PBS with 2% normal goat serum and incubated for 30–45 min on ice to label the immune population (CD45) and the epithelial population (epithelial cell adhesion molecule). Cells were centrifuged for 5 min at 800 RCF at 4 °C and pushed through a 35 μm cell strainer. The FACS Aria III Special Ordered Research Product (BD Biosciences) was used to sort confetti^+^ and confetti cells, by applying a broad FSC/SSC gate, followed by gates excluding doublets (for the gating strategy, see Extended Data Fig. 1d). Afterwards, non-immune (AF647^–^; 670/30) confetti-positive (RFP^+^ (YG610/20), GFP^+^/YFP^+^ (BL530/30), CFP^+^ (V450/50)) and, separately, confetti-negative ((RFP^–^ (YG610/20), GFP^–^/YFP^–^ (BL530/30), CFP^–^ (V450/50)) epithelial cells (APC/Cy7^+^; 780/60) were collected. Similarly, non-immune (AF647^–^; 670/30) epithelial cells (APC/Cy7^+^; 780/60) were collected from three *R26R-Confetti;Brca1*^*fl/fl*^*;Trp53*^*fl/fl*^ mice that had not received TAT-Cre intraductally as a negative control. Data were analysed in FlowJo v.10 for the gating strategy (Extended Data Fig. 1d). Cells were spun down for 10 min at 800 RCF at 4 °C and DNA was isolated using the PicoPure DNA extraction kit (Applied Biosciences; KIT0103) according to the manufacturer’s instructions. The same method was applied to isolate genomic DNA (gDNA) from *K14-Cre;Brca1*^*fl/fl*^*;Trp53*^*fl/fl*^ mammary tumour organoids, representing fully recombined samples as a positive control. gDNA concentration was measured using the DeNovix DS-11 spectrophotometer. DNA was diluted to 50–75 ng ml^−1^ and used for real-time qPCR using the SYBR Green Master Mix (ThermoFisher Scientific, catalogue no. 4309155) in a QuantStudio 6 Flex Real-Time PCR system using the primers listed in the table below. Reactions contained roughly 75 ng of template gDNA and 1 µM of both forward and reverse primers in 20 µl reaction volume. Expression values were calculated by transforming delta–delta Ct values (2^-ΔΔCt^). Ribosomal Protein L38 (Rpl38) was used as a housekeeping gene. To confirm correct qPCR product amplification, 25 μl of qPCR product of the control samples (*R26R-Confetti;Brca1*^*fl/fl*^*;Trp53*^*fl/fl*^ mice that had not received TAT-Cre intraductally) was loaded on an 2% agarose gel with loading buffer (Bioxline, catalogue no. BIO-37045) and a DNA ladder (Meridian Bioscience, catalogue no. BIO-33056) and run at 80 V for 1.5 h, after which the qPCR product was cut out of the gel and purified using the NucleoSpin Gel and PCR Clean-up kit (Macherey-Nagel, catalogue no. 740609.50) according to the manufacturer’s instructions, and was confirmed by sequencing using the qPCR primers.

**Table:** 

Primer	Sequence (5′ → 3′)
BRCA1-ex10-FW	TGTAACGACAGGCAGGTTCC
BRCA1-ex10-RV	ACAGAGTTTGCGGGTGAGTC
P53-ex5-FW	AAGACGTGCCCTGTGCAGTT
P53-ex5-RV	TCCGTCATGTGCTGTGACTTC
RPL38_FW	AGGATGCCAAGTCTGTCAAGA
RPL38_RV	TCCTTGTCTGTGATAACCAGGG

### Generation of *Brca1;Trp53* mutant and WT organoids followed by Ki-67/CC3 staining

The fourth and fifth mammary glands of *R26R-Confetti;Brca1*^*fl/fl*^*;Trp53*^*fl/fl*^ mice were intraductally injected with recombinant TAT-Cre protein (20 units per gland diluted in 20 µl PBS, Sigma-Aldrich) between 10 and 15 weeks of age. Then, 64 or 225 days post-induction, mammary glands were harvested and prepared for fluorescence-activated cell sorting (FACS) as described before. Both confetti-positive *Brca1*^*−/−*^*;Trp53*^*−/−*^ and non-recombined control cells were seeded in a 24-well plate, 10,000 cells per drop of Cultrex Basement Membrane Extract (Type 2, 3532-010-02, R&D Systems) and cultured in the DMEM/F12 (Gibco) supplemented with iInsulin-transferrin-selenium (100×, catalogue no. 41400045, Gibco), B-27 Supplement (50×, catalogue no. 17504044, Gibco), NAC 1.25 mM (*N*-acetyl-l-cysteine, 0.125 M in PBS, catalogue no. 6169116, Biogems), mFGF2 2.5 nM (Fibroblast Growth Factor 2, catalogue no. 100-18B, PeproTech) and mEGF 2 nM (Epidermal Growth Factor, catalogue no. 3165-09, PeproTech). After 2 weeks of culture, organoids were fixed with 4% PFA (catalogue no. 47347, AlfaAesar) for 10–15 min at room temperature inside the Basement Membrane Extract droplet on an orbital shaker at 25 rpm. Afterwards, organoids were washed three times for 10 min with PBS, followed by incubation in permeabilization buffer (5% normal goat serum (catalogue no. 16210072, Gibco) and 0.5% Triton X-100 ((Sigma-Aldrich) in PBS) for 3 h. To stain for cell proliferation and cell death, primary antibodies Ki-67 (rat, clone SolA15, 14-5698-82, eBioscience, 1:100) and Cleaved Caspase-3 (rabbit, Asp175, no. 9661, Cell Signaling Technology, 1:400), respectively, were added in the blocking buffer (5% normal goat serum (Gibco) in PBS), and incubated overnight at 4 °C. Organoids were washed three times for 15 min with PBS and secondary antibodies goat antirabbit IgG Antibody, Alexa Fluor 647 (catalogue no. A21244, Thermo Scientific, 1:400), goat antirat IgG Antibody, Alexa Fluor 647 (catalogue no. A21247, Thermo Scientific, 1:400) were added and incubated for more than 5 h at room temperature covered in aluminium foil on an orbital shaker at 25 rpm. Organoids were washed three times for 15 min with PBS and stained organoids were mounted by adding 200 µl of Vectashield mounting medium (VECTASHIELD HardSet Antifade Mounting Medium, H-1400, Vector Laboratories). Organoid imaging was performed on an inverted Leica SP8 Dive system (Leica Microsystems), in which Alexa-647 secondary antibodies were excited at 635 nm and detected between 660 and 700 nm, and organoids were imaged using brightfield. The Ki-67/CC3 ratio was derived by first calculating the organoid area and Ki-67^+^ or CC3^+^ areas using ImageJ software (https://imagej.nih.gov/ij/), then calculating the percentage of Ki-67 or CC3 expressing cells per organoid, followed by calculation of the Ki-67/CC3 ratio for every organoid.

### Statistics

*P* values and statistical tests performed are included in the figure legends or Supplementary Information 1. The longitudinal data of the clone fractions (Figs. 2d and 5g) was analysed using a regression model with a time effect, for which the interaction between time and group was tested. For full details, see Supplementary Information 2. Details on statistics concerning the mathematical modelling can be found in the Supplementary Information 4.

### Reporting summary

Further information on research design is available in the Nature Portfolio Reporting Summary linked to this article.

## Online content

Any methods, additional references, Nature Portfolio reporting summaries, source data, extended data, supplementary information, acknowledgements, peer review information; details of author contributions and competing interests; and statements of data and code availability are available at 10.1038/s41586-024-07882-3.

## Supplementary information

Supplementary Information 1 Overview of sample sizes, *P* values and statistical tests.

Reporting Summary

Supplementary Information 2 Longitudinal data statistics. Statistical analysis code used to compare the different groups in Figs. 2d and 5g.

Supplementary Information 3 Genomic alterations in *Brca1;Trp53* confetti clones. Individual DNA copy number profiles of each 225d *Brca1;Trp53* clone and chromosome. The profiles are sorted by clone transformation status as indicated.

Supplementary Information 4 This file contains a Supplementary Note and Sections 1–5 including Figs. 1–10 and Tables.

## Source data

Source Data Fig. 1

Source Data Fig. 2

Source Data Fig. 3

Source Data Fig. 4

Source Data Fig. 5

Source Data Extended Data Fig. 1

Source Data Extended Data Fig. 4

Source Data Extended Data Fig. 5

Source Data Extended Data Fig. 6

Source Data Extended Data Fig. 7

Source Data Extended Data Fig. 8

Source Data Extended Data Fig. 9

Source Data Extended Data Fig. 10

Source Data Extended Data Fig. 11

Source Data Extended Data Fig. 12

## Data Availability

The raw clonal data are all provided in the source data. DNA sequencing data are available at the European Genome-Phenome Archive (ENA, https://www.ebi.ac.uk/ena/browser/home) under accession number PRJEB71510, secondary accession ERP156311 and PRJEB30443 (Sample accession numbers SAMEA5202116 – 5202120, 5202122 – 5202126). Source data are provided with this paper.

## Code availability

The procedures used to fit the parameters of the phenomenological theory to the experimental data are defined in Supplementary Information 4. The basis of the cell-based model is also defined in Supplementary Information 4. Stochastic simulations of the cell-based model were made using a dedicated Fortran code and the Mathematica software package. The code for the computational and statistical analyses is deposited on the GitHub repository (https://github.com/BenSimonsLab/Ciwinska_Nature_2024). Data supporting the findings of Fig. 4b and Extended Data Fig. 9a, including computer code for stochastic simulations, are available at https://github.com/BioImaging-NKI/qupath_ripley. NET code for branching analysis used in Extended Data Fig. 7 is available from J.v.R on reasonable request. Code to determine longitudinal data statistics is provided in the Supplementary Information 2.
