# Supplementary material for: Mechanisms that clear mutations drive field cancerization in mammary tissue
Source: Nature. 2024 Sep 4;633(8028):198–206. doi: 10.1038/s41586-024-07882-3 (PMC11374684; doi:10.1038/s41586-024-07882-3)
Supplement: Supplementary file 1 — Overview of sample sizes, P values and statistical tests. [file 41586_2024_7882_MOESM1_ESM.pdf]

nature portfolio

https://doi.org/10.1038/s41586-024-07882-3

Supplementary information

Mechanisms that clear mutations drive field cancerization in mammary tissue

In the format provided by the
authors and unedited

Supplementary File 1, Overview of sample sizes, p-values and statistical tests.

Figure	Group	Number of mice used / number of clones	Exact p value and statistical test
1e	14 days Luminal<br>14 days Basal<br>64 days Luminal<br>64 days Basal<br>120 days Luminal<br>120 days Basal<br>225 days Luminal<br>225 days Basal	4 / 578<br>4 / 129<br>4 / 387<br>4 / 57<br>5 / 392<br>5 / 39<br>6 / 135<br>6 / 28	Min: 0 Max: 0 Median: 0<br>Min: 0 Max: 0 Median: 0<br>Min: 0 Max: 0.0737 Median: 0.0095<br>Min: 0 Max: 0 Median: 0<br>Min: 0.0206 Max: 0.0930 Median: 0.0500<br>Min: 0 Max: 0 Median: 0<br>Min: 0.0790 Max: 0.2000 Median: 0.1548<br>Min: 0 Max: 0.0833 Median: 0
1g	14 days<br>64 days<br>120 days<br>225 days	4 / 707<br>4 / 444<br>5 / 431<br>6 / 163	NA
2c	*Brca1;Trp53 Confetti*<br>14 days Luminal<br>14 days Basal<br>64 days Luminal<br>64 days Basal<br>120 days Luminal<br>120 days Basal<br>225 days Luminal<br>225 days Basal<br><br>Wild-type *Confetti*<br>14 days Luminal<br>14 days Basal<br>64 days Luminal<br>64 days Basal<br>120 days Luminal<br>120 days Basal<br>225 days Luminal<br>225 days Basal	 4 / 578<br>4 / 129<br>4 / 387<br>4 / 57<br>5 / 392<br>5 / 39<br>6 / 135<br>6 / 28<br><br><br>3 / 981<br>3 / 99<br>3 / 99<br>3 / 39<br>5 / 313<br>5 / 157<br>6 / 482<br>6 / 80	Two-sided Mann-Whitney U Test<br><br>14 days *Brca1;Trp53 Confetti* vs<br>14 days Wild-type *Confetti*<br>P value < 0.0001<br><br>64 days *Brca1;Trp53 Confetti* vs<br>64 days Wild-type *Confetti*<br>P value < 0.0001<br><br>120 days *Brca1;Trp53 Confetti* vs<br>120 days Wild-type *Confetti*<br>P value < 0.0001<br><br>225 days *Brca1;Trp53 Confetti* vs<br>225 days Wild-type *Confetti*<br>P value < 0.0001
2d	*Brca1;Trp53 Confetti*<br>14 days Luminal<br>14 days Basal<br>64 days Luminal<br>64 days Basal<br>120 days Luminal<br>120 days Basal<br>225 days Luminal<br>225 days Basal<br><br>Wild-type *Confetti*<br>14 days Luminal<br>14 days Basal<br>64 days Luminal<br>64 days Basal<br>120 days Luminal	Number of mice<br>3<br>3<br>4<br>4<br>4<br>4<br>4<br>4<br><br><br>3<br>3<br>3<br>3<br>4	Fit of regression model followed by test of the coefficient of interaction between time and group.<br><br>For details see Supplementary information, part 2, Longitudinal data analysis.

	120 days Basal<br>225 days Luminal<br>225 days Basal	4<br>4<br>4	
3c, d	Wild-type *Confetti*<br>Luminal clones<br>Mouse 1 – 120 days<br>Mouse 2 – 120 days<br>Mouse 3 – 120 days<br>Mouse 4 – 120 days<br>Mouse 5 – 120 days<br><br>Mouse 1 – 225 days<br>Mouse 2 – 225 days<br>Mouse 3 – 225 days<br>Mouse 4 – 225 days<br>Mouse 5 – 225 days<br><br>Mouse 1 – 550 days<br>Mouse 2 – 550 days<br>Mouse 3 – 550 days<br>Mouse 4 – 550 days<br><br>Basal clones<br>Mouse 1 – 64 days<br><br>Mouse 1 – 120 days<br>Mouse 2 – 120 days<br><br>Mouse 1 – 225 days<br>Mouse 2 – 225 days<br><br>Mouse 1 – 550 days<br>Mouse 2 – 550 days<br>Mouse 3 – 550 days<br><br>*Brca1;Trp53* confetti<br>Luminal clones<br>Mouse 1 – 14 days<br>Mouse 2 – 14 days<br>Mouse 3 – 14 days<br>Mouse 4 – 14 days<br><br>Mouse 1 – 64 days<br>Mouse 2 – 64 days<br>Mouse 3 – 64 days<br>Mouse 4 – 64 days<br><br>Mouse 1 – 120 days<br>Mouse 2 – 120 days<br>Mouse 3 – 120 days<br>Mouse 4 – 120 days<br>Mouse 5 – 120 days<br><br>Mouse 1 – 225 days<br>Mouse 2 – 225 days	Number of clones<br><br>129 clones<br>17 clones<br>54 clones<br>30 clones<br>83 clones<br><br>185 clones<br>90 clones<br>52 clones<br>92 clones<br>61 clones<br><br>150 clones<br>120 clones<br>16 clones<br>28 clones<br><br><br>23 clones<br><br>23 clones<br>88 clones<br><br>20 clones<br>51 clones<br><br>105 clones<br>74 clones<br>13 clones<br><br><br><br>115 clones<br>227 clones<br>90 clones<br>146 clones<br><br>76 clones<br>123 clones<br>91 clones<br>88 clones<br><br>84 clones<br>19 clones<br>117 clones<br>56 clones<br>95 clones<br><br>35 clones<br>15 clones	Bars in c show variance +/- SE<br><br>Bars in d show mean values +/- SEM.

	Mouse 3 – 225 days<br>Mouse 4 – 225 days<br>Mouse 5 – 225 days<br><br>Basal clones<br>Mouse 1 – 14 days<br>Mouse 2 – 14 days<br>Mouse 3 – 14 days<br>Mouse 4 – 14 days<br><br>Mouse 1 – 64 days<br>Mouse 2 – 64 days<br>Mouse 3 – 64 days<br>Mouse 4 – 64 days<br><br>Mouse 1 – 120 days<br>Mouse 2 – 120 days<br>Mouse 3 – 120 days<br>Mouse 4 – 120 days<br>Mouse 5 – 120 days<br><br>Mouse 1 – 225 days<br>Mouse 2 – 225 days<br>Mouse 3 – 225 days<br>Mouse 4 – 225 days	32 clones<br>20 clones<br>12 clones<br><br><br>46 clones<br>43 clones<br>22 clones<br>18 clones<br><br>12 clones<br>23 clones<br>16 clones<br>6 clones<br><br>8 clones<br>2 clones<br>10 clones<br>11 clones<br>8 clones<br><br>8 clones<br>4 clones<br>4 clones<br>11 clones	
4g	 Mouse 1<br>Mouse 2<br>Mouse 3	Number of regions<br>9<br>7<br>10	Two-sided paired t-test, p = 0.6483
4h	 Mouse 1<br>Mouse 2<br>Mouse 3	Number of regions<br>15<br>10<br>10	Two-sided paired t-test, p = 0.1097
4i, j	 Mouse 1<br>Mouse 2<br>Mouse 3	Number of regions<br>7<br>9<br>10	i, Two-sided paired t-test, p = 0.0059<br><br>j, Box defines mean, bars indicate +/- SD
5a	Wild-type *confetti*<br>Oestrous cycle<br>14 days Luminal<br>14 days Basal<br>64 days Luminal<br>64 days Basal<br>120 days Luminal<br>120 days Basal<br>225 days Luminal<br>225 days Basal<br><br>Ovariectomy<br>14 days Luminal<br>14 days Basal<br>64 days Luminal<br>64 days Basal	Number of mice/clones<br><br>3 / 981<br>3 / 99<br>3 / 99<br>3 / 39<br>5 / 313<br>5 / 157<br>6 / 482<br>6 / 80<br><br><br>4 / 2703<br>4 / 174<br>3 / 437<br>3 / 160	Two-sided Mann-Whitney U Test<br><br>14 days Oestrous cycle vs<br>14 days Ovariectomy<br>P value < 0.0001<br><br>64 days Oestrous cycle vs<br>64 days Ovariectomy<br>P value < 0.0001<br><br>120 days Oestrous cycle vs<br>120 days Ovariectomy<br>P value < 0.0001<br><br>225 days Oestrous cycle vs<br>225 days Ovariectomy

	120 days Luminal<br>120 days Basal<br>225 days Luminal<br>225 days Basal	3 / 73<br>3 / 43<br>3 / 407<br>3 / 137	P value < 0.0001
5d	*Brca1;Trp53 Confetti*<br>Oestrous cycle<br>14 days Luminal<br>14 days Basal<br>64 days Luminal<br>64 days Basal<br>120 days Luminal<br>120 days Basal<br>225 days Luminal<br>225 days Basal<br><br>Ovariectomy<br>14 days Luminal<br>14 days Basal<br>64 days Luminal<br>64 days Basal<br>120 days Luminal<br>120 days Basal<br>225 days Luminal<br>225 days Basal	Number of<br>mice/clones<br>4 / 578<br>4 / 129<br>4 / 387<br>4 / 57<br>5 / 392<br>5 / 39<br>6 / 135<br>6 / 28<br><br><br>3 / 769<br>3 / 166<br>3 / 609<br>3 / 64<br>3 / 969<br>3 / 161<br>3 / 337<br>3 / 81	Two-sided Mann-Whitney U Test<br><br>14 days Oestrous cycle vs<br>14 days Ovariectomy<br>P value < 0.0001<br><br>64 days Oestrous cycle vs<br>64 days Ovariectomy<br>P value < 0.0001<br><br>120 days Oestrous cycle vs<br>120 days Ovariectomy<br>P value < 0.0001<br><br>225 days Oestrous cycle vs<br>225 days Ovariectomy<br>P value < 0.0001
5g	*Brca1;Trp53 Confetti*<br>Ovariectomy<br>14 days Luminal<br>14 days Basal<br>64 days Luminal<br>64 days Basal<br>120 days Luminal<br>120 days Basal<br>225 days Luminal<br>225 days Basal<br><br>Wild-type *Confetti*<br>Ovariectomy<br>14 days Luminal<br>14 days Basal<br>64 days Luminal<br>64 days Basal<br>120 days Luminal<br>120 days Basal<br>225 days Luminal<br>225 days Basal	Number of mice<br><br>3<br>3<br>2<br>2<br>4<br>4<br>3<br>3<br><br><br><br>3<br>3<br>3<br>3<br>4<br>4<br>4<br>4	From a longitudinal data analysis, between the *Brca1;Trp53* and wild-type clones there is a marginally significant difference in the basal group (p=0.06), and a statistically significant difference in the luminal group p<0.01.<br><br>For details see Supplementary information, part 2, Longitudinal data analysis.
5h	 <br>14 days<br>64 days<br>120 days<br>225 days	Number of<br>mice/clones<br>3 / 735<br>3 / 673<br>3 / 1130<br>3 / 418	NA
5i	 <br>Oestrous cycle	Number of<br>mice/clones	Significance was tested using a two-sided Mann-Whitney U test, P = 0.0238

	Luminal<br>Basal<br><br>Ovariectomy<br>Luminal<br>Basal	6 / 135<br>6 / 28<br><br><br>3 / 337<br>3 / 81	Min: 0.0790 Max: 0.2000 Median: 0.1548<br>Min: 0 Max: 0.0833 Median: 0<br><br><br>Min: 0 Max: 0.0179 Median: 0.004<br>Min: 0 Max: 0 Median: 0
ED5E	*Brca1;Trp53 Confetti*<br>14 days Luminal<br>64 days Luminal<br>120 days Luminal<br>225 days Luminal<br><br>14 days Luminal<br>64 days Luminal<br>120 days Luminal<br>225 days Luminal	 4 / 578<br>4 / 387<br>5 / 392<br>6 / 135<br><br>3 / 981<br>3 / 99<br>5 / 313<br>6 / 482	Two-sided Mann-Whitney U Test<br><br>14 days *Brca1;Trp53 Confetti* vs<br>14 days Wild-type *Confetti*<br>P value < 0.0001<br><br>64 days *Brca1;Trp53 Confetti* vs<br>64 days Wild-type *Confetti*<br>P value < 0.0001<br><br>120 days *Brca1;Trp53 Confetti* vs<br>120 days Wild-type *Confetti*<br>P value < 0.0001<br><br>225 days *Brca1;Trp53 Confetti* vs<br>225 days Wild-type *Confetti*<br>P value < 0.0001
ED 5F	 <br>*Brca1;Trp53 Confetti*<br>14 days Basal<br>64 days Basal<br>120 days Basal<br>225 days Basal<br><br>Wild-type *Confetti*<br>14 days Basal<br>64 days Basal<br>120 days Basal<br>225 days Basal	Number of mice/clones<br><br><br>4 / 129<br>4 / 57<br>5 / 39<br>6 / 28<br><br><br>3 / 99<br>3 / 39<br>5 / 157<br>6 / 80	Two-sided Mann-Whitney U Test<br><br>14 days *Brca1;Trp53 Confetti* vs<br>14 days Wild-type *Confetti*<br>P value = 0.0013<br><br>64 days *Brca1;Trp53 Confetti* vs<br>64 days Wild-type *Confetti*<br>P value < 0.0001<br><br>120 days *Brca1;Trp53 Confetti* vs<br>120 days Wild-type *Confetti*<br>P value < 0.0001<br><br>225 days *Brca1;Trp53 Confetti* vs<br>225 days Wild-type *Confetti*<br>P value = 0.0211
ED8e	Wild-type *Confetti*<br>Luminal clones<br>Mouse 1 – 120 days<br>Mouse 2 – 120 days<br>Mouse 3 – 120 days<br>Mouse 4 – 120 days<br>Mouse 5 – 120 days<br><br>Mouse 1 – 225 days<br>Mouse 2 – 225 days<br>Mouse 3 – 225 days<br>Mouse 4 – 225 days	Number of clones<br><br>129 clones<br>17 clones<br>54 clones<br>30 clones<br>83 clones<br><br>185 clones<br>90 clones<br>52 clones<br>92 clones	Bars in e show mean values +/- SEM.

	Mouse 5 – 225 days<br><br>Mouse 1 – 550 days<br>Mouse 2 – 550 days<br>Mouse 3 – 550 days<br>Mouse 4 – 550 days	61 clones<br><br>150 clones<br>120 clones<br>16 clones<br>28 clones	
ED8f	Wild-type *Confetti*<br>Luminal clones<br>Mouse 1 – 120 days<br>Mouse 2 – 120 days<br>Mouse 3 – 120 days<br>Mouse 4 – 120 days<br>Mouse 5 – 120 days<br><br>Mouse 1 – 225 days<br>Mouse 2 – 225 days<br>Mouse 3 – 225 days<br>Mouse 4 – 225 days<br>Mouse 5 – 225 days<br><br>Mouse 1 – 550 days<br>Mouse 2 – 550 days	Number of clones<br><br>129 clones<br>17 clones<br>54 clones<br>30 clones<br>83 clones<br><br>185 clones<br>90 clones<br>52 clones<br>92 clones<br>61 clones<br><br>150 clones<br>120 clones	Bars in f show mean values +/- SEM.
ED9e	Wild-type *Confetti*<br>Duct<br>225 days luminal<br>225 days basal<br><br>Side branch<br>225 days luminal<br>225 days basal<br><br>*Brca1;Trp53 Confetti*<br>Duct<br>225 days luminal<br>225 days basal<br><br>Side branch<br>225 days luminal<br>225 days basal	Number of mice /<br>clones<br>6 / 325<br>6 / 44<br><br><br>6 / 156<br>6 / 26<br><br><br><br>6 / 110<br>6 / 24<br><br><br>6 / 24<br>6 / 4	Two-sided Mann-Whitney U Test<br><br>Wild-type duct vs. side branch P = 0.9236<br><br>*Brca1;Trp53* duct vs. side branch P < 0.0001
ED9f	*Brca1;Trp53 Confetti*<br><br><br>Duct<br>225 days luminal<br>225 days basal<br><br>Side branch<br>225 days luminal<br>225 days basal	Number of mice<br><br><br><br>6<br>6<br><br><br>6<br>6	Two-sided Mann Whitney U test<br>Luminal duct vs. Luminal Side branch<br>P = 0.0022<br><br>Min: 0 Max: 0.100 Median: 0.0278<br>Min: 0 Max: 0.1111 Median: 0.000<br><br><br>Min: 0.1667 Max: 1.000 Median: 0.4167<br>Min: 0 Max: 0 Median : 0
ED10b	 *Brca1;Trp53 Confetti*<br>Nulliparous<br>120 days luminal<br>120 days basal	Number of mice /<br>clones<br><br>5 / 392<br>5 / 39	Two-sided Mann-Whitney U Test<br><br>P < 0.0001

	Brca1;Trp53 Confetti<br>Parous<br>120 days luminal<br>120 days basal	 <br>3 / 565<br>3 / 6	
ED10c	Nulliparous<br>Luminal<br>Basal<br><br>Parous<br>Luminal<br>Basal	Number of mice<br>5<br>5<br><br><br>3<br>3	 Min: 0.0206 Max: 0.0930 Median: 0.0500<br>Min: 0 Max: 0 Median: 0<br><br><br>Min: 0 Max: 0 Median: 0<br>Min: 0 Max: 0 Median: 0
ED11a	Wild-type *Confetti*<br>Oestrous cycle<br>14 days Luminal<br>64 days Luminal<br>120 days Luminal<br>225 days Luminal<br><br>Ovariectomy<br>14 days Luminal<br>64 days Luminal<br>120 days Luminal<br>225 days Luminal	Number of<br>mice/clones<br>3 / 981<br>3 / 99<br>5 / 313<br>6 / 482<br><br><br>4 / 2703<br>3 / 437<br>3 / 73<br>3 / 407	Two-sided Mann-Whitney U Test<br><br>14 days Luminal oestrous cycle vs<br>14 days Luminal ovariectomy<br>P value < 0.0001<br><br>64 days Luminal oestrous cycle vs<br>64 days Luminal ovariectomy<br>P value < 0.0001<br><br>120 days Luminal oestrous cycle vs<br>120 days Luminal ovariectomy<br>P value < 0.0001<br><br>225 days Luminal oestrous cycle vs<br>225 days Luminal ovariectomy<br>P value < 0.0001
ED11b	Wild-type *Confetti*<br>Oestrous cycle<br>14 days Basal<br>64 days Basal<br>120 days Basal<br>225 days Basal<br><br>Ovariectomy<br>14 days Basal<br>64 days Basal<br>120 days Basal<br>225 days Basal	Number of<br>mice/clones<br>3 / 99<br>3 / 39<br>5 / 157<br>6 / 80<br><br><br>4 / 174<br>3 / 160<br>3 / 43<br>3 / 137	Two-sided Mann-Whitney U Test<br><br>14 days Basal oestrous cycle vs<br>14 days Basal ovariectomy<br>P = 0.0852<br><br>64 days Basal oestrous cycle vs<br>64 days Basal ovariectomy<br>P = 0.7089<br><br>120 days Basal oestrous cycle vs<br>120 days Basal ovariectomy<br>P = 0.6935<br><br>225 days Basal Oestrous cycle vs<br>225 days Basal ovariectomy<br>P value < 0.0001
ED11c	*Brca1;Trp53 Confetti*<br>Oestrous cycle<br>14 days Luminal<br>64 days Luminal<br>120 days Luminal<br>225 days Luminal	Number of<br>mice/clones<br>4 / 578<br>4 / 387<br>5 / 392<br>6 / 135	Two-sided Mann-Whitney U Test<br><br>14 days Luminal oestrous cycle vs<br>14 days Luminal ovariectomy<br>P value < 0.0001

	Ovariectomy<br>14 days Luminal<br>64 days Luminal<br>120 days Luminal<br>225 days Luminal	 3 / 769<br>3 / 609<br>3 / 969<br>3 / 337	64 days Luminal oestrous cycle vs<br>64 days Luminal ovariectomy<br>P value < 0.0001<br><br>120 days Luminal oestrous cycle vs<br>120 days Luminal ovariectomy<br>P value < 0.0001<br><br>225 days Luminal oestrous cycle vs<br>225 days Luminal ovariectomy<br>P value < 0.0001
ED11d	*Brca1;Trp53 Confetti*<br>Oestrous cycle<br>14 days Basal<br>64 days Basal<br>120 days Basal<br>225 days Basal<br><br>Ovariectomy<br>14 days Basal<br>64 days Basal<br>120 days Basal<br>225 days Basal	 <br>4 / 129<br>4 / 57<br>5 / 39<br>6 / 28<br><br><br>3 / 166<br>3 / 64<br>3 / 161<br>3 / 81	Two-sided Mann-Whitney U Test<br><br>14 days Basal oestrous cycle vs<br>14 days Basal ovariectomy<br>P = 0.0001<br><br>64 days Basal oestrous cycle vs<br>64 days Basal ovariectomy<br>P value < 0.0001<br><br>120 days Basal oestrous cycle vs<br>120 days Basal ovariectomy<br>P value < 0.0001<br><br>225 days Basal oestrous cycle vs<br>225 days Basal ovariectomy<br>P value < 0.0001
