# Supplementary material for: Mechanisms that clear mutations drive field cancerization in mammary tissue
Source: Nature. 2024 Sep 4;633(8028):198–206. doi: 10.1038/s41586-024-07882-3 (PMC11374684; doi:10.1038/s41586-024-07882-3)
Supplement: Supplementary file 2 — Reporting Summary [file 41586_2024_7882_MOESM2_ESM.pdf]

nature portfolio

Corresponding author(s): Jacco van Rheenen, Benjamin Simons, Colinda Scheele

Last updated by author(s): 2024_06_23

Reporting Summary

Nature Portfolio wishes to improve the reproducibility of the work that we publish. This form provides structure for consistency and transparency in reporting. For further information on Nature Portfolio policies, see our Editorial Policies and the Editorial Policy Checklist.

Statistics

For all statistical analyses, confirm that the following items are present in the figure legend, table legend, main text, or Methods section.

n/a	Confirmed	
☐	☒	The exact sample size (n) for each experimental group/condition, given as a discrete number and unit of measurement
☐	☒	A statement on whether measurements were taken from distinct samples or whether the same sample was measured repeatedly
☐	☒	The statistical test(s) used AND whether they are one- or two-sided<br>*Only common tests should be described solely by name; describe more complex techniques in the Methods section.*
☒	☐	A description of all covariates tested
☒	☐	A description of any assumptions or corrections, such as tests of normality and adjustment for multiple comparisons
☐	☒	A full description of the statistical parameters including central tendency (e.g. means) or other basic estimates (e.g. regression coefficient) AND variation (e.g. standard deviation) or associated estimates of uncertainty (e.g. confidence intervals)
☐	☒	For null hypothesis testing, the test statistic (e.g. F, t, r) with confidence intervals, effect sizes, degrees of freedom and P value noted<br>*Give P values as exact values whenever suitable.*
☒	☐	For Bayesian analysis, information on the choice of priors and Markov chain Monte Carlo settings
☒	☐	For hierarchical and complex designs, identification of the appropriate level for tests and full reporting of outcomes
☒	☐	Estimates of effect sizes (e.g. Cohen's d, Pearson's r), indicating how they were calculated

Our web collection on statistics for biologists contains articles on many of the points above.

Software and code

Policy information about availability of computer code

Data collection	Leica Application Suite X (Leica Microsystems, version 3.7.6.25997), BD FacsDIVA software (version 9.0)
Data analysis	QuPath version 0.4.1, Graphpad Prism version 10, custom made script: https://github.com/BioImaging-NKI/qupath_ripley, ImageJ version 1.54f, Imaris Viewer version 10.1, LasX 3D visualization module, version 3.7, .NET software version 4.5, FlowJo version 10, Python version 3.12.0, ggplot2 version 3.5.1<br><br>The procedures used to fit the parameters of the phenomenological theory to the experimental data are defined in the Supplementary Theory. The basis of the cell-based model is also defined in the Supplementary Theory. Stochastic simulations of the cell-based model were made using a dedicated Fortran code and the Mathematica software package. The code for the computational and statistical analyses is deposited on the GitHub repository (https://github.com/BenSimonsLab/Ciwinska_Nature_2024). Data supporting the findings of Figure 4b and Extended Data Figure 9a, including computer code for stochastic simulations, are available at https://github.com/BioImaging-NKI/qupath_ripley. .NET code for branching analysis used in Extended Data Figure 7 is available from J.v.R upon reasonable request. Code to determine longitudinal data statistics is provided in the Supplementary File 1, File 2.

For manuscripts utilizing custom algorithms or software that are central to the research but not yet described in published literature, software must be made available to editors and reviewers. We strongly encourage code deposition in a community repository (e.g. GitHub). See the Nature Portfolio guidelines for submitting code & software for further information.

Data

Policy information about availability of data

All manuscripts must include a data availability statement. This statement should provide the following information, where applicable:

- Accession codes, unique identifiers, or web links for publicly available datasets
- A description of any restrictions on data availability
- For clinical datasets or third party data, please ensure that the statement adheres to our policy

Source data for Figures 1e, g, 2c, d, 3a-d, 4b, g-j, 5a,d,g-i, Extended Data Figures 1a, 4c,e, 5e, f, 6b, 7c,d, 8a-f, 9d-j, 10b,c, 11a-d, and 12a-d are provided in the Source Data Files. The raw clonal data are all provided in the source data. DNA sequencing data are available at the European Genome-Phenome Archive (ENA, https://www.ebi.ac.uk/ena/browser/home) under accession number PRJEB71510, secondary accession ERP156311 and PRJEB30443 (Sample accession SAMEA5202116 – 5202120, 5202122 – 5202126).

Research involving human participants, their data, or biological material

Policy information about studies with human participants or human data. See also policy information about sex, gender (identity/presentation), and sexual orientation and race, ethnicity and racism.

Reporting on sex and gender	NA
Reporting on race, ethnicity, or other socially relevant groupings	NA
Population characteristics	NA
Recruitment	NA
Ethics oversight	NA

Note that full information on the approval of the study protocol must also be provided in the manuscript.

Field-specific reporting

Please select the one below that is the best fit for your research. If you are not sure, read the appropriate sections before making your selection.

☒ Life sciences ☐ Behavioural & social sciences ☐ Ecological, evolutionary & environmental sciences

For a reference copy of the document with all sections, see nature.com/documents/nr-reporting-summary-flat.pdf

Life sciences study design

All studies must disclose on these points even when the disclosure is negative.

Sample size	Sample size was not determined a priori. With clone size quantifications it is difficult to predict effect size and standard deviation a priori. therefore, we used a resource equation approach to get an estimation of the required numbers of mice, using clone sizes as quantitative values. This approach predicted the requirement of at least 3 animals per group. However, due to the nature of our clonal lineage tracing approach (only few recombined cells per gland), we observed large variability in terms of clone number between glands, even within the same animal. In some cases, we included more replicates to achieve a sufficient clone number for downstream analysis. To account for variability between mice, at least two different mammary glands were analysed per mouse. All groups have at least n = 3 mice per condition.
Data exclusions	We have excluded the following data from analysis:<br>- For the experimental fits (page 14, supplementary theory) we have excluded mutant clones that have already undergone transformation, which were infrequent in the luminal population and negligible in the basal population.<br>- For the clonal analysis (Figure 2C and related figure panels) we excluded the palpable lesions for analysis (lines 144-145 of the manuscript).
Replication	All experiments were repeated at least 3 times, all attempts at replication were successful.
Randomization	Samples were randomly allocated to the experimental groups.
Blinding	Researchers were not blinded to experimental conditions. For the Brca1;Trp53 conditions, mice needed to be checked for tumor formation, hence researchers could not be blinded to control or mutant conditions. However, during data quantification, researchers were blinded to the conditions they were analysing.

Reporting for specific materials, systems and methods

We require information from authors about some types of materials, experimental systems and methods used in many studies. Here, indicate whether each material, system or method listed is relevant to your study. If you are not sure if a list item applies to your research, read the appropriate section before selecting a response.

Materials & experimental systems

n/a	Involved in the study
☐	☒ Antibodies
☒	☐ Eukaryotic cell lines
☒	☐ Palaeontology and archaeology
☐	☒ Animals and other organisms
☒	☐ Clinical data
☒	☐ Dual use research of concern
☒	☐ Plants

Methods

n/a	Involved in the study
☒	☐ ChIP-seq
☐	☒ Flow cytometry
☒	☐ MRI-based neuroimaging

Antibodies

Antibodies used

Primary antibodies: anti-KRT8 (rat, Troma-I, Merck Millipore, 1:800) anti-KRT14 (rabbit, Covance, PRB155P, 1:700), anti-E-cadherin (rat, eBioscience, 14-3249-82, 1:700), anti-ER (rabbit, #13258, Cell Signaling, 1:100), anti-PR (rabbit, Clone SP2, MA5-14505, ThermoFisher Scientific, 1:200), anti-Smooth muscle actin (mouse IgG2a, clone 1A4, Sigma-Aldrich, 1:600), anti-Ki-76 (rat, SolA15, eBioscience, 1:100) and anti-Cleaved Caspase-3 (rabbit, Asp175, Cell Signaling Technology, 1:400).

Alexa Fluor 647 and Alexa Fluor 488 Phalloidin were used 1:500 (A-22287 and A-12379, ThermoFisher Scientific) and incubated together with the secondary antibodies.

Secondary antibodies: goat anti-rabbit, goat anti-rat, or goat anti-mouse IgG2a, all conjugated to Alexa-647 (ThermoFisher Scientific, A21244, A21247, and A21241 respectively, 1:400), donkey anti-rat Alexa-488, donkey anti-mouse Alexa546 (ThermoFisher Scientific, A21208 and A10036 respectively, 1:400).

FACS antibodies: CD45-Alexa647 (Biolegend; clone 30-F11; cat. No: 103123; 1:200), EpCAM-APC/Cy7 (Biolegend; clone G8.8; cat. No: 118218; 1:200),

Validation

All antibodies used were tested for use in immunofluorescence in murine samples according to the manufacturers information.

Animals and other research organisms

Policy information about studies involving animals; ARRIVE guidelines recommended for reporting animal research, and Sex and Gender in Research

Laboratory animals

All mice used for experiments were adult females (injected between 10-15 weeks of age) from a mixed background, housed under standard laboratory conditions, and receiving food and water ad libitum.

Strains:
R26R-Confetti (JAX stock #013731), R26-CreERT2 (JAX stock # 008463), Brca1fl/fl;Trp53fl/fl (PMID: 11694875, PMID: 17626182), CAG;;KikGR (RIKEN #CLSTCDB0201T-117830853340), R26-mTmG (JAX #007676).

Wild animals

No wild animals were used in the study.

Reporting on sex

All mice used in this study were adult female mice.

Field-collected samples

No field collected samples were used in the study.

Ethics oversight

All experiments were performed in accordance with the guidelines of the Animal Welfare Committee of the Royal Netherlands Academy of Arts and Sciences, the Netherlands Cancer Institute, and KU Leuven.

Note that full information on the approval of the study protocol must also be provided in the manuscript.

Plants

Seed stocks

NA

Novel plant genotypes

NA

Authentication

NA

Flow Cytometry

Plots

Confirm that:

☒ The axis labels state the marker and fluorochrome used (e.g. CD4-FITC).

☒ The axis scales are clearly visible. Include numbers along axes only for bottom left plot of group (a 'group' is an analysis of identical markers).

☒ All plots are contour plots with outliers or pseudocolor plots.

☒ A numerical value for number of cells or percentage (with statistics) is provided.

Methodology

Sample preparation

The 3rd, 4th and 5th mammary glands of R26R-Confetti;Brca1fl/fl;Trp53fl/fl mice were intraductally injected with recombinant TAT-Cre protein (20 units per gland diluted in 20 µl PBS, produced in-house) between 10-13 weeks of age. 120 to 180 days after injection mammary glands were harvested, minced and digested at 37°C for 30 min in a mixture of collagenase A (2 mg/ml, Roche Diagnostics), hyaluronidase (300 µg/ml, Sigma Aldrich) and DNAse (1mg/ml) in DMEM/F12 (Gibco). After 10 min incubation with TripLE (Gibco) at 37°C cells were strained through a 100µm cell strainer (Fisher scientific) to obtain single cells. Cells were spun down for 10 minutes at 550 rcf at 4°C followed by blocking for 15 min on ice in 5 mM EDTA/PBS with 2% sterile filtered normal goat serum (Gibco). CD45-Alexa647 (Biolegend; clone 30-F11; cat. No: 103123; 1:200) and EpCAM-APC/Cy7 (Biolegend; clone G8.8; cat. No: 118218; 1:200) were diluted in 5 mM EDTA/PBS with 2% normal goat serum and incubated for 30-45 min on ice to label the immune population (CD45) and the epithelial population (EpCAM). Cells were centrifuged for 5 min at 800 RCF at 4°C and pushed through a 35 µm cell strainer. FACS Aria III Special Ordered Research Product (BD Biosciences) was used to sort confetti+ and confetti- cells, by applying a broad FSC/SSC gate, followed by gates excluding doublets (for gating strategy, see Extended Data Figure 1d). Afterwards, non-immune (AF647-; 670/30) confetti positive (RFP+ (YG610/20), GFP+/YFP+ (BL530/30), CFP+ (V450/50)) and, separately, confetti negative ((RFP- (YG610/20), GFP-/YFP- (BL530/30), CFP- (V450/50)) epithelial cells (APC/Cy7+; 780/60) were collected. Similarly, non-immune (AF647-; 670/30) epithelial cells (APC/Cy7+; 780/60) were collected from three R26R-Confetti;Brca1fl/fl;Trp53fl/fl mice that had not received TAT-Cre intraductally as a negative control.

Instrument

FACS Aria III Special Ordered Research Product (BD Biosciences)

Software

FlowJo

Cell population abundance

The abundance of Brca1;Trp53;Confetti+ cells in our combined fraction consisting of GFP_YFP, RFP and CFP gated populations was >95%. The purity of Confetti- cells was >99%. These fractions were determined by fluorescent imaging of the sorted cells. Epithelial nature of the sorted cells was determined by staining of the cells post-sorting for Epcam+, which was >99%.

Gating strategy

Extended Data Figure 1D

☒ Tick this box to confirm that a figure exemplifying the gating strategy is provided in the Supplementary Information.
