# Supplementary material for: Mechanisms that clear mutations drive field cancerization in mammary tissue
Source: Nature. 2024 Sep 4;633(8028):198–206. doi: 10.1038/s41586-024-07882-3 (PMC11374684; doi:10.1038/s41586-024-07882-3)
Supplement: Supplementary file 3 — Longitudinal data statistics. Statistical analysis code used to compare the different groups in Figs. 2d and 5g. [file 41586_2024_7882_MOESM3_ESM.pdf]

Introduction
Figure 2D
Figure 5G
Comparison ovariectomy vs baseline

Code

Analyses manuscript Ciwinska et al.

Renee Menezes r.menezes@nki.nl (mailto:r.menezes@nki.nl)

2024-06-25

Introduction

Clone fraction data in different time points were collected for different groups. The data are displayed in figures 2D and 5G.

For each figure, the data is analysed as follows:

- per group, clone fractions along time are taken together
- a regression model is fitted, with both time as well as the interaction between time and group included. The intention is to fit the time trend, which we represent with a linear function
- conclusions about comparisons between groups are drawn based upon the test of the coefficient of the interaction between time and group: if this is statistically significant from 0, then we conclude that the groups have different time trends.

Show

Figure 2D

Show

Change labels of groups.

Show

The data is changed from the wide format to the long format.

Show

Wild-type vs. Brca1 Trp53

Comparing wild-type to Brca1;Trp53 groups, given either luminal or basal backgrounds.

First for the basal group.

Show

```
##
## Call:
## lm(formula = value ~ time + time:group, data = mydata)
##
## Residuals:
##      Min       1Q   Median       3Q      Max
## -0.43003 -0.22251  0.07162  0.24765  0.28138
##
## Coefficients:
##                             Estimate Std. Error t value Pr(>|t|)
## (Intercept)                0.7751997  0.0758446  10.221 5.93e-11 ***
## time                      -0.0035634  0.0006647  -5.361 1.04e-05 ***
## time:groupWild-type basal -0.0004778  0.0006774  -0.705    0.486
## ---
## Signif. codes:  0 '***' 0.001 '**' 0.01 '*' 0.05 '.' 0.1 ' ' 1
##
## Residual standard error: 0.2512 on 28 degrees of freedom
## Multiple R-squared:  0.6176, Adjusted R-squared:  0.5902
## F-statistic: 22.61 on 2 and 28 DF,  p-value: 1.432e-06
```

Conclusion: there is no significant effect between Basal_wild-type and Basal_Brca1; Trp53 (p>0.10).

Now for the same comparison between luminal subgroups.

Show

```
##
## Call:
## lm(formula = value ~ time + time:group, data = mydata)
##
## Residuals:
##      Min       1Q   Median       3Q      Max
## -0.46050 -0.18077  0.06324  0.25919  0.41731
##
## Coefficients:
##                               Estimate Std. Error t value Pr(>|t|)
## (Intercept)                  0.7884799  0.0778777  10.125 7.32e-11 ***
## time                        -0.0034047  0.0006825  -4.988 2.86e-05 ***
## time:groupWild-type luminal -0.0004146  0.0006955  -0.596    0.556
## ---
## Signif. codes:  0 '***' 0.001 '**' 0.01 '*' 0.05 '.' 0.1 ' ' 1
##
## Residual standard error: 0.258 on 28 degrees of freedom
## Multiple R-squared:  0.5797, Adjusted R-squared:  0.5497
## F-statistic: 19.31 on 2 and 28 DF,  p-value: 5.362e-06
```

Conclusion: there is no significant difference between Luminal_wild-type and Luminal_Brca1;Trp53 groups (p>0.10).

Figure 5G

Read in data

Show

Change labels of groups.

Show

The data is changed from the wide format to the long format.

Show

Wild-type vs. Brca1;Trp53

Comparing wild-type to Brca1;Trp53 groups, given either luminal or basal backgrounds. In all cases, this refers to ovariectomy clonal fractions.

First for the basal group.

Show

```
##
## Call:
## lm(formula = value ~ time + time:group, data = mydata)
##
## Residuals:
##      Min       1Q   Median       3Q      Max
## -0.45216 -0.17948  0.04374  0.08024  0.52965
##
## Coefficients:
##                                      Estimate Std. Error t value Pr(>|t|)
## (Intercept)                         0.9962560  0.0691596  14.405  1.3e-13
## time                               -0.0028569  0.0006330  -4.514 0.000131
## time:groupOvariectomy wild-type basal -0.0012226  0.0006307  -1.939 0.063914
##
## (Intercept)                        ***
## time                               ***
## time:groupOvariectomy wild-type basal .
## ---
## Signif. codes:  0 '***' 0.001 '**' 0.01 '*' 0.05 '.' 0.1 ' ' 1
##
## Residual standard error: 0.2197 on 25 degrees of freedom
## Multiple R-squared:  0.667,  Adjusted R-squared:  0.6403
## F-statistic: 25.04 on 2 and 25 DF,  p-value: 1.073e-06
```

Conclusion: there is a marginally significant effect between Basal_wild-type and Basal_Brca1;Trp53 (p<0.10).

Now for the same comparison between luminal subgroups.

Show

```
##
## Call:
## lm(formula = value ~ time + time:group, data = mydata)
##
## Residuals:
##     Min      1Q  Median      3Q     Max
## -0.3521 -0.1956  0.1220  0.1495  0.2505
##
## Coefficients:
##                                          Estimate Std. Error t value Pr(>|t|)
## (Intercept)                             0.9137842  0.0654943  13.952 2.66e-13
## time                                   -0.0025535  0.0005994  -4.260 0.000253
## time:groupOvariectomy wild-type luminal -0.0019604  0.0005972  -3.282 0.003035
##
## (Intercept)                             ***
## time                                    ***
## time:groupOvariectomy wild-type luminal **
## ---
## Signif. codes:  0 '***' 0.001 '**' 0.01 '*' 0.05 '.' 0.1 ' ' 1
##
## Residual standard error: 0.208 on 25 degrees of freedom
## Multiple R-squared:  0.7248, Adjusted R-squared:  0.7028
## F-statistic: 32.92 on 2 and 25 DF,  p-value: 9.913e-08
```

Conclusion: there is a statistically significant difference between Luminal_wild-type and Luminal_Brca1;Trp53 groups (p<0.01).

Comparison ovariectomy vs baseline

We now compare the baseline clonal fractions (figure 2D) with the corresponding ovariectomy fractions (figure 7G).

Show

Perform comparisons as before: fit model with time and an interaction between time and group, selecting the group according to the labels in `group` . Then compare the ovariectomy values with baseline.

The test results for the comparisons:

Show

```
##   Wild-type luminal     Wild-type basal Brca1;Trp53 luminal   Brca1;Trp53 basal
##               0.941               0.081               0.011               0.004
```
