# Supplementary material for: Mechanisms that clear mutations drive field cancerization in mammary tissue
Source: Nature. 2024 Sep 4;633(8028):198–206. doi: 10.1038/s41586-024-07882-3 (PMC11374684; doi:10.1038/s41586-024-07882-3)
Supplement: Supplementary file 4 — Genomic alterations in Brca1;Trp53 confetti clones. Individual DNA copy number profiles of each 225d Brca1;Trp53 clone and chromosome. The profiles are sorted by clone transformation status as indicated. [file 41586_2024_7882_MOESM4_ESM.pdf]

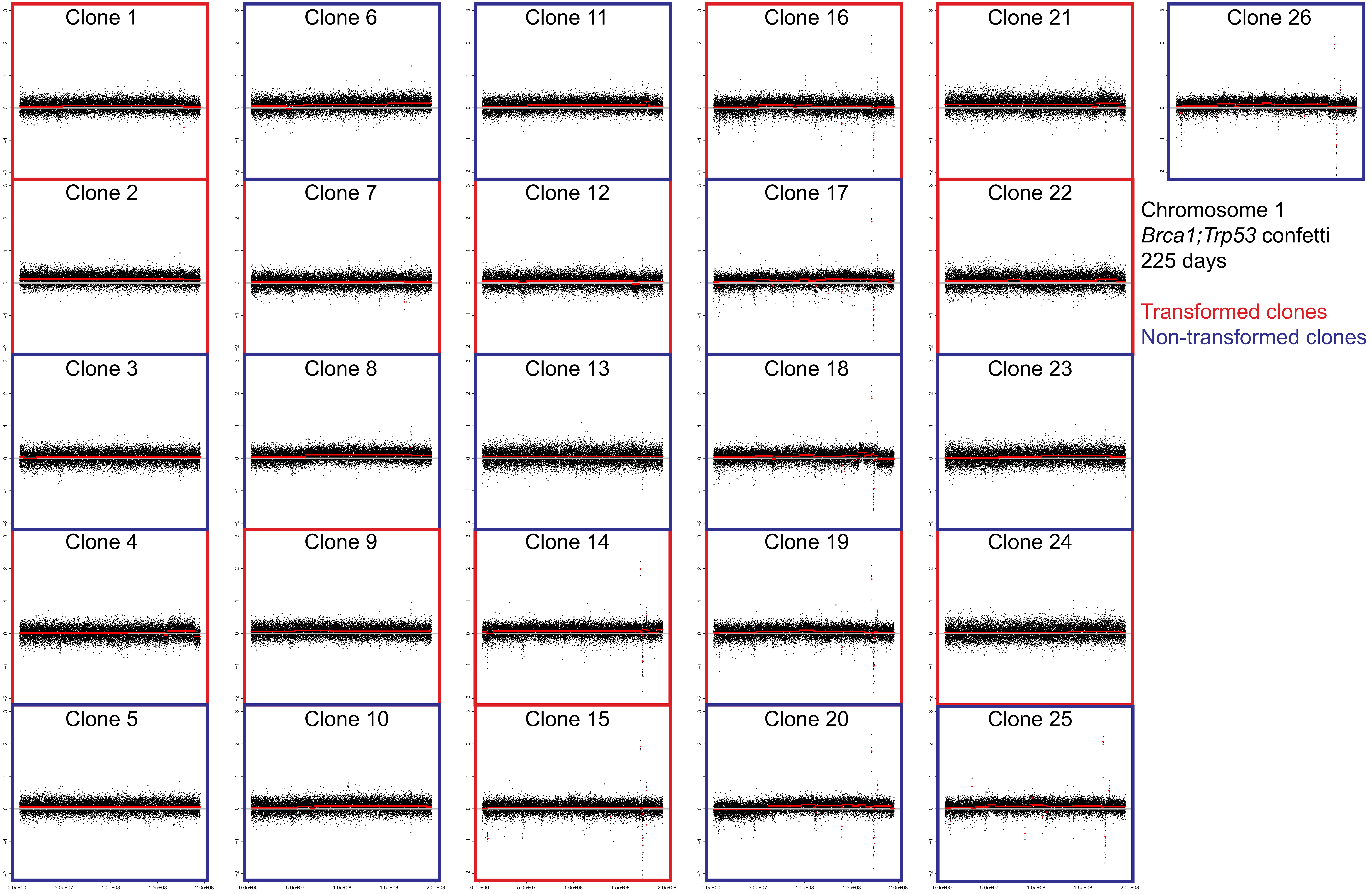
Clone 1
Clone 2
Clone 3
Clone 4
Clone 5
Clone 6
Clone 7
Clone 8
Clone 9
Clone 10
Clone 11
Clone 12
Clone 13
Clone 14
Clone 15
Clone 16
Clone 17
Clone 18
Clone 19
Clone 20
Clone 21
Clone 22
Clone 23
Clone 24
Clone 25
Clone 26
Chromosome 1
Brca1;Trp53 confetti
225 days
Transformed clones
Non-transformed clones
0.0e+00
5.0e+07
1.0e+08
1.5e+08
2.0e+08

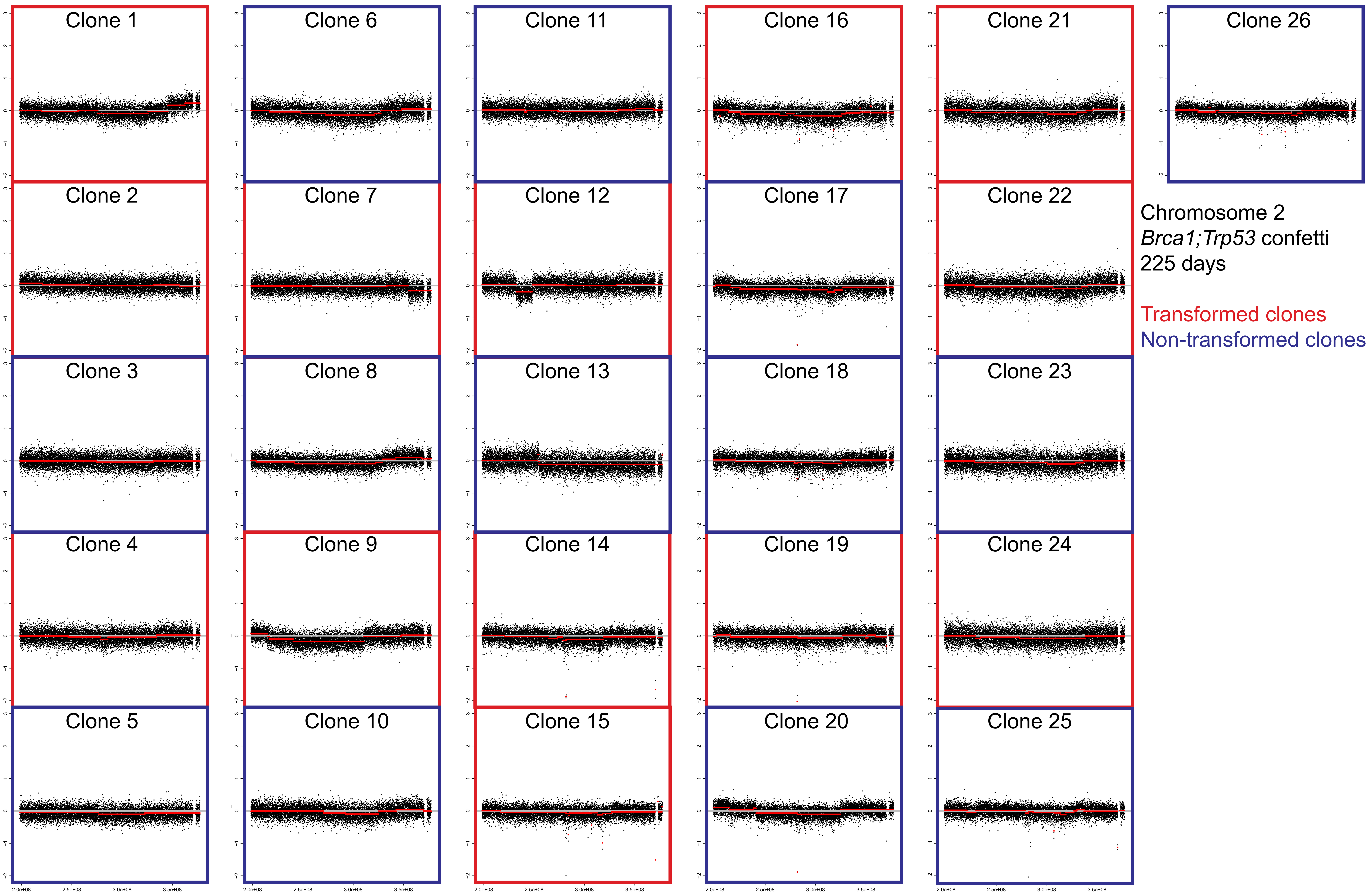
Clone 1
Clone 2
Clone 3
Clone 4
Clone 5
Clone 6
Clone 7
Clone 8
Clone 9
Clone 10
Clone 11
Clone 12
Clone 13
Clone 14
Clone 15
Clone 16
Clone 17
Clone 18
Clone 19
Clone 20
Clone 21
Clone 22
Clone 23
Clone 24
Clone 25
Clone 26
Chromosome 2
Brca1;Trp53 confetti
225 days
Transformed clones
Non-transformed clones

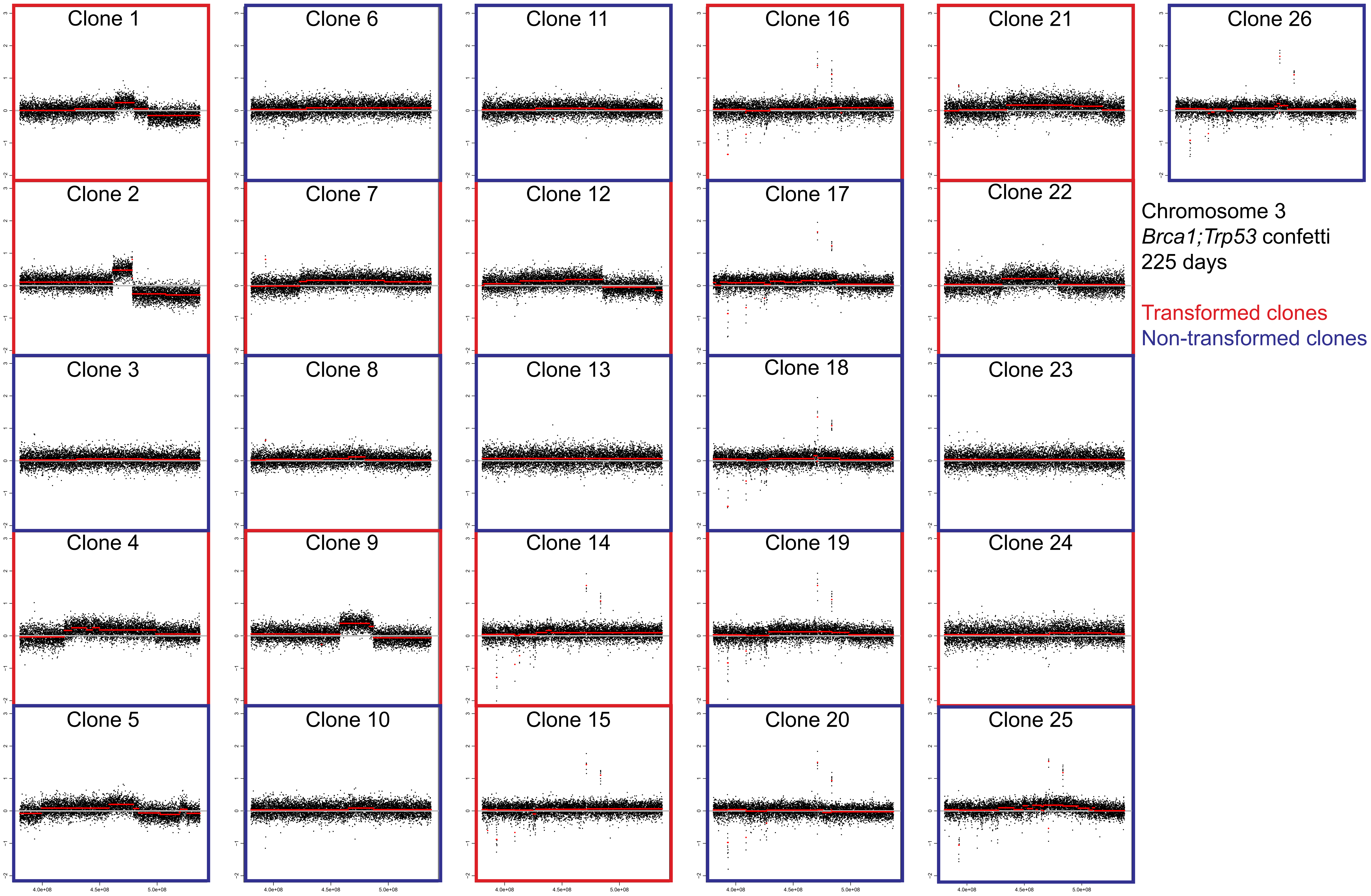

Clone 1
Clone 6
Clone 11
Clone 16
Clone 21
Clone 26
Clone 2
Clone 7
Clone 12
Clone 17
Clone 22
Clone 3
Clone 8
Clone 13
Clone 18
Clone 23
Clone 4
Clone 9
Clone 14
Clone 19
Clone 24
Clone 5
Clone 10
Clone 15
Clone 20
Clone 25
Chromosome 3
Brca1;Trp53 confetti
225 days
Transformed clones
Non-transformed clones

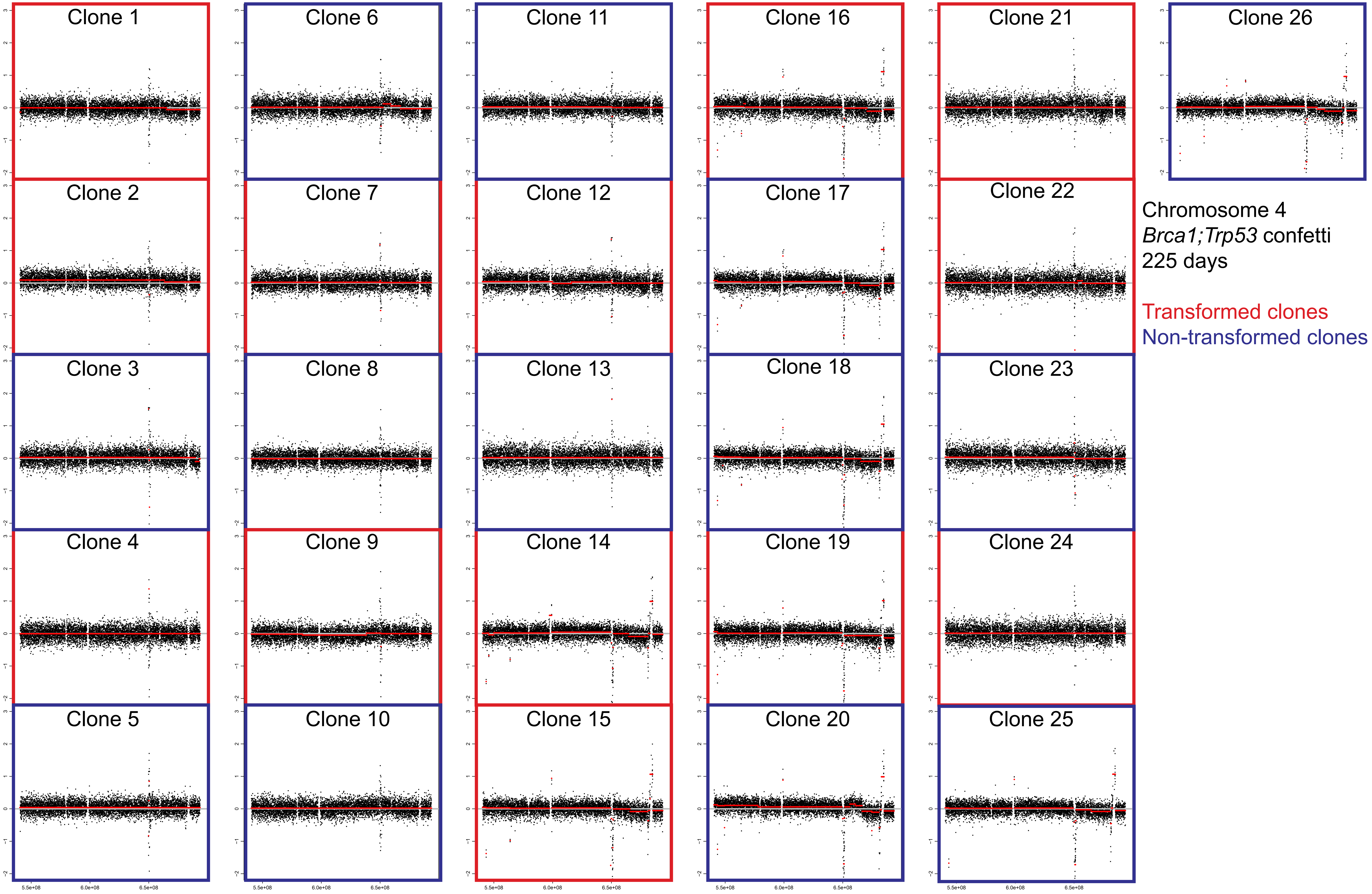

Clone 1
Clone 2
Clone 3
Clone 4
Clone 5
Clone 6
Clone 7
Clone 8
Clone 9
Clone 10
Clone 11
Clone 12
Clone 13
Clone 14
Clone 15
Clone 16
Clone 17
Clone 18
Clone 19
Clone 20
Clone 21
Clone 22
Clone 23
Clone 24
Clone 25
Clone 26
Chromosome 4
Brca1;Trp53 confetti
225 days
Transformed clones
Non-transformed clones
5.5e+08
6.0e+08
6.5e+08

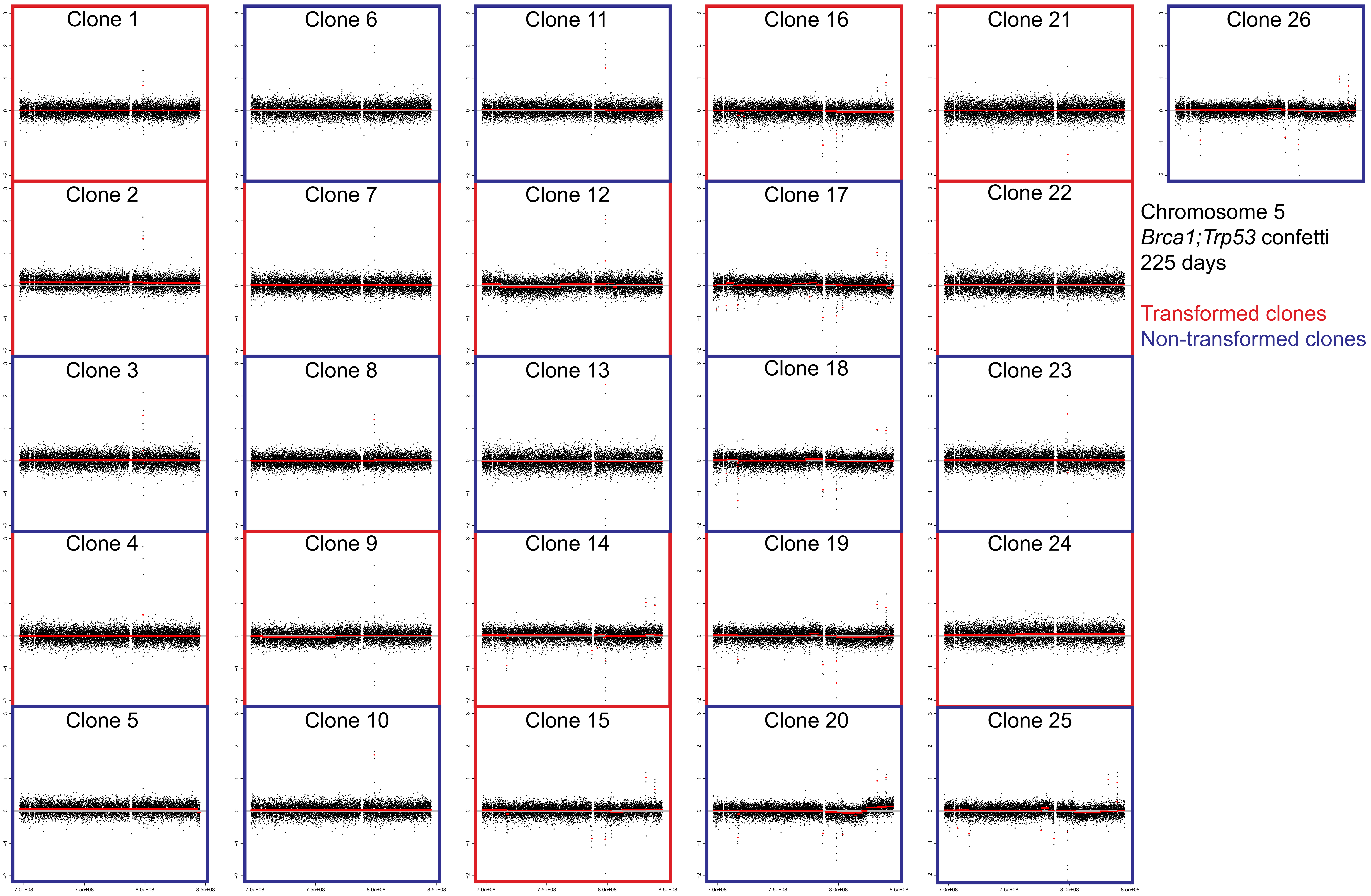
Clone 1
Clone 2
Clone 3
Clone 4
Clone 5
Clone 6
Clone 7
Clone 8
Clone 9
Clone 10
Clone 11
Clone 12
Clone 13
Clone 14
Clone 15
Clone 16
Clone 17
Clone 18
Clone 19
Clone 20
Clone 21
Clone 22
Clone 23
Clone 24
Clone 25
Clone 26
Chromosome 5
Brca1;Trp53 confetti
225 days
Transformed clones
Non-transformed clones

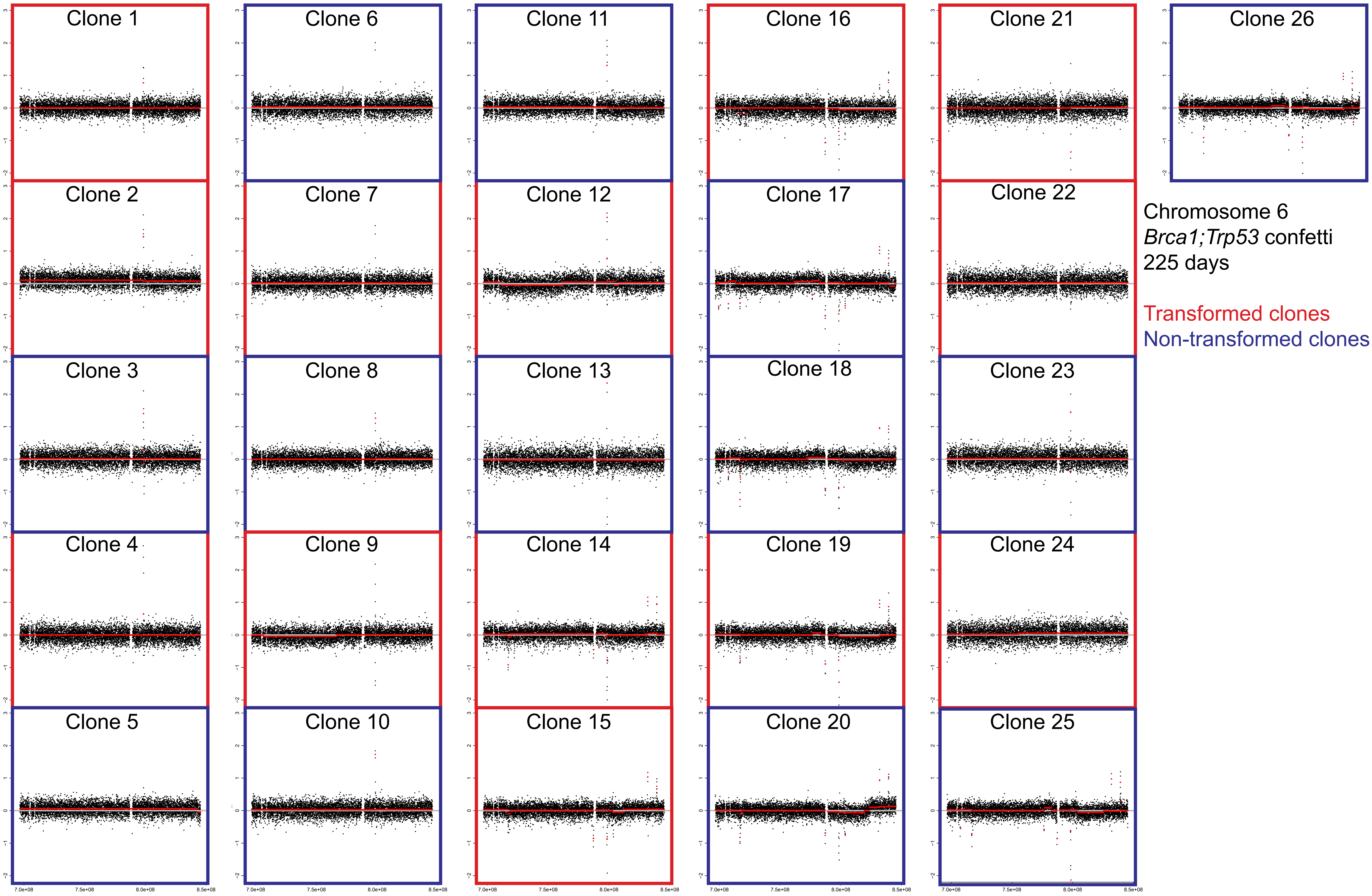

Clone 1
Clone 2
Clone 3
Clone 4
Clone 5
Clone 6
Clone 7
Clone 8
Clone 9
Clone 10
Clone 11
Clone 12
Clone 13
Clone 14
Clone 15
Clone 16
Clone 17
Clone 18
Clone 19
Clone 20
Clone 21
Clone 22
Clone 23
Clone 24
Clone 25
Clone 26
Chromosome 6
Brca1;Trp53 confetti
225 days
Transformed clones
Non-transformed clones

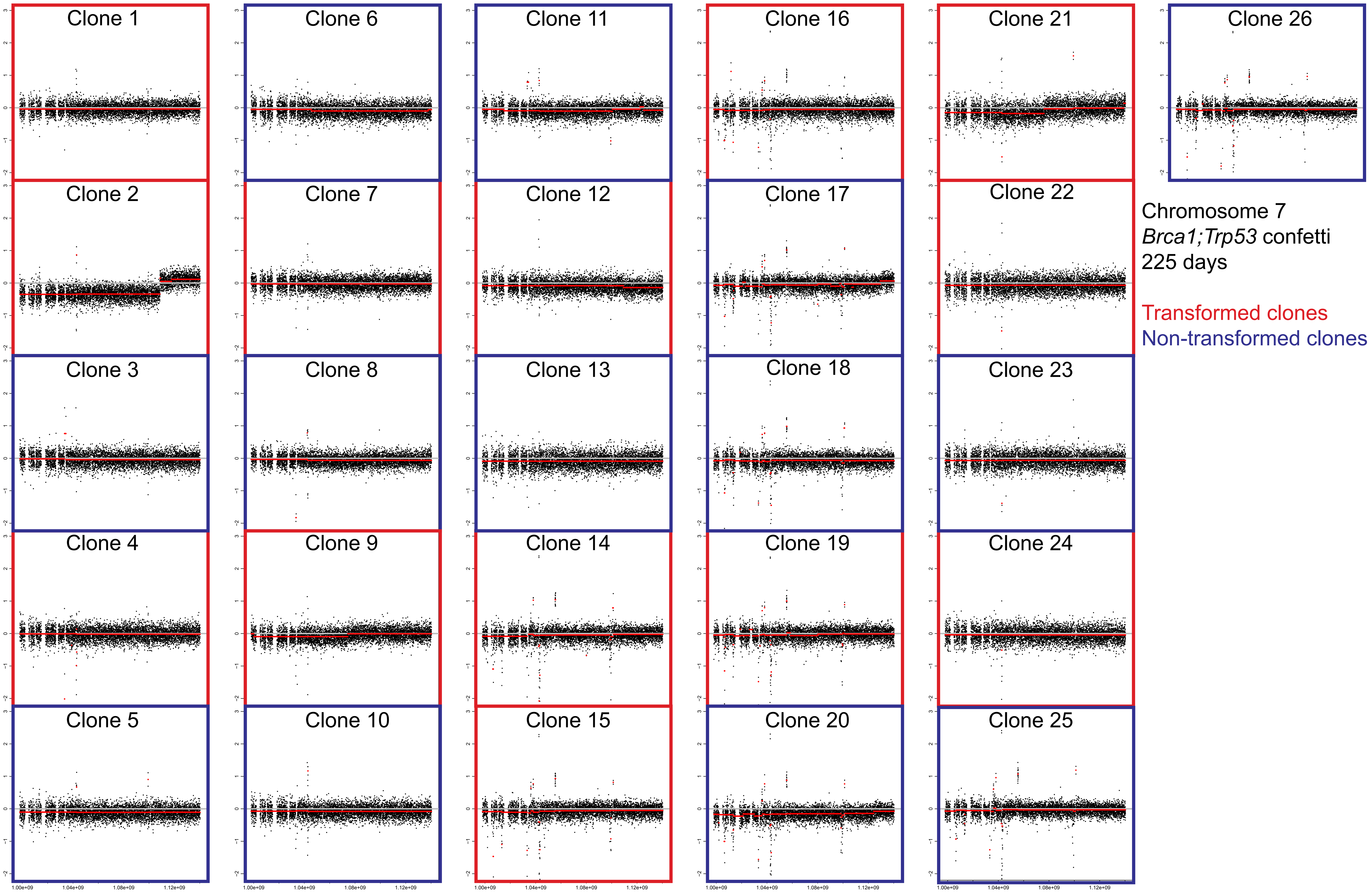
Clone 1
Clone 2
Clone 3
Clone 4
Clone 5
Clone 6
Clone 7
Clone 8
Clone 9
Clone 10
Clone 11
Clone 12
Clone 13
Clone 14
Clone 15
Clone 16
Clone 17
Clone 18
Clone 19
Clone 20
Clone 21
Clone 22
Clone 23
Clone 24
Clone 25
Clone 26
1.00e+09
1.04e+09
1.08e+09
1.12e+09
Chromosome 7
Brca1;Trp53 confetti
225 days
Transformed clones
Non-transformed clones

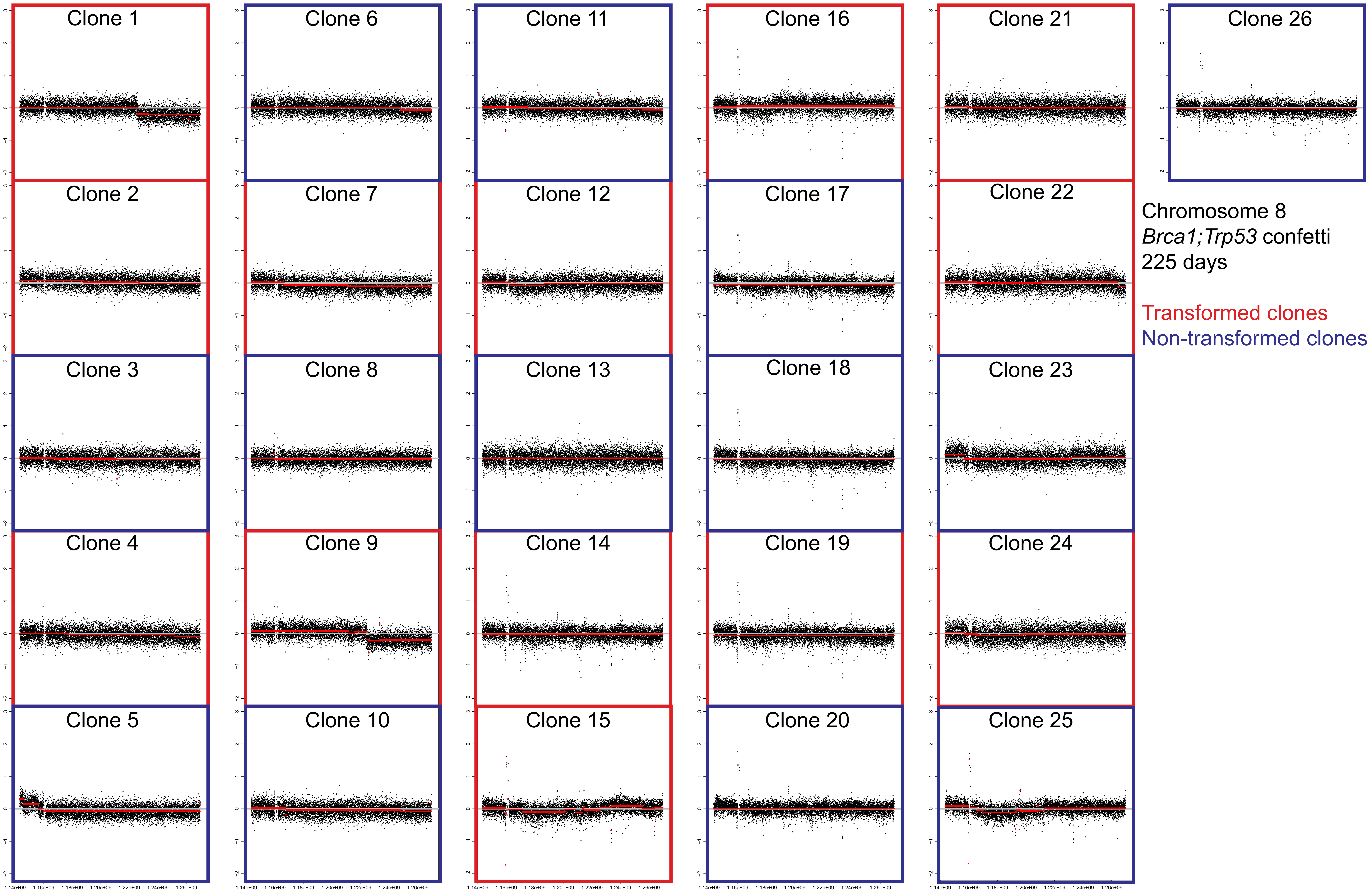
Clone 1
Clone 6
Clone 11
Clone 16
Clone 21
Clone 26
Clone 2
Clone 7
Clone 12
Clone 17
Clone 22
Clone 3
Clone 8
Clone 13
Clone 18
Clone 23
Clone 4
Clone 9
Clone 14
Clone 19
Clone 24
Clone 5
Clone 10
Clone 15
Clone 20
Clone 25
Chromosome 8
Brca1;Trp53 confetti
225 days
Transformed clones
Non-transformed clones

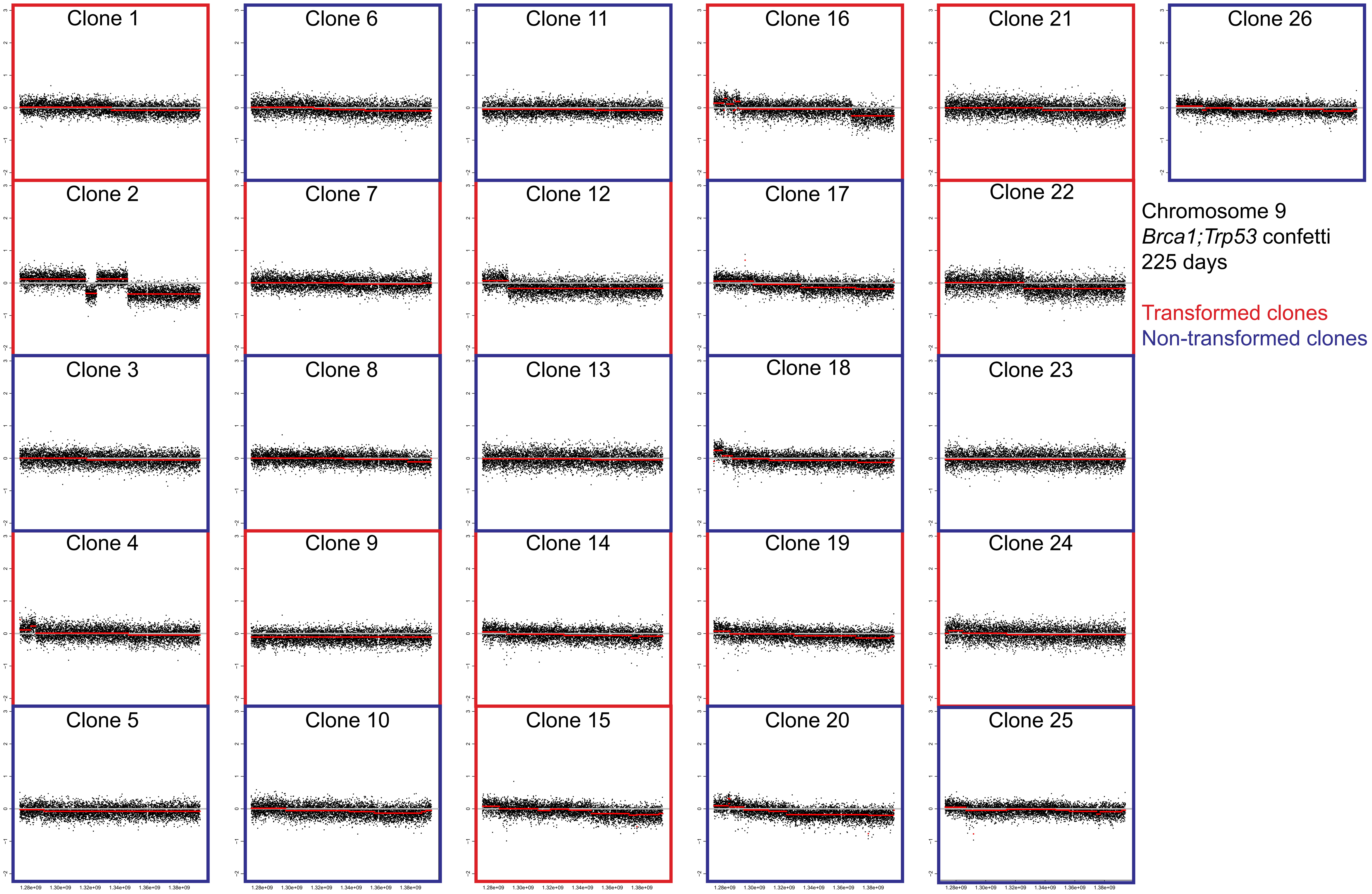
Clone 1
Clone 2
Clone 3
Clone 4
Clone 5
Clone 6
Clone 7
Clone 8
Clone 9
Clone 10
Clone 11
Clone 12
Clone 13
Clone 14
Clone 15
Clone 16
Clone 17
Clone 18
Clone 19
Clone 20
Clone 21
Clone 22
Clone 23
Clone 24
Clone 25
Clone 26
Chromosome 9
Brca1;Trp53 confetti
225 days
Transformed clones
Non-transformed clones

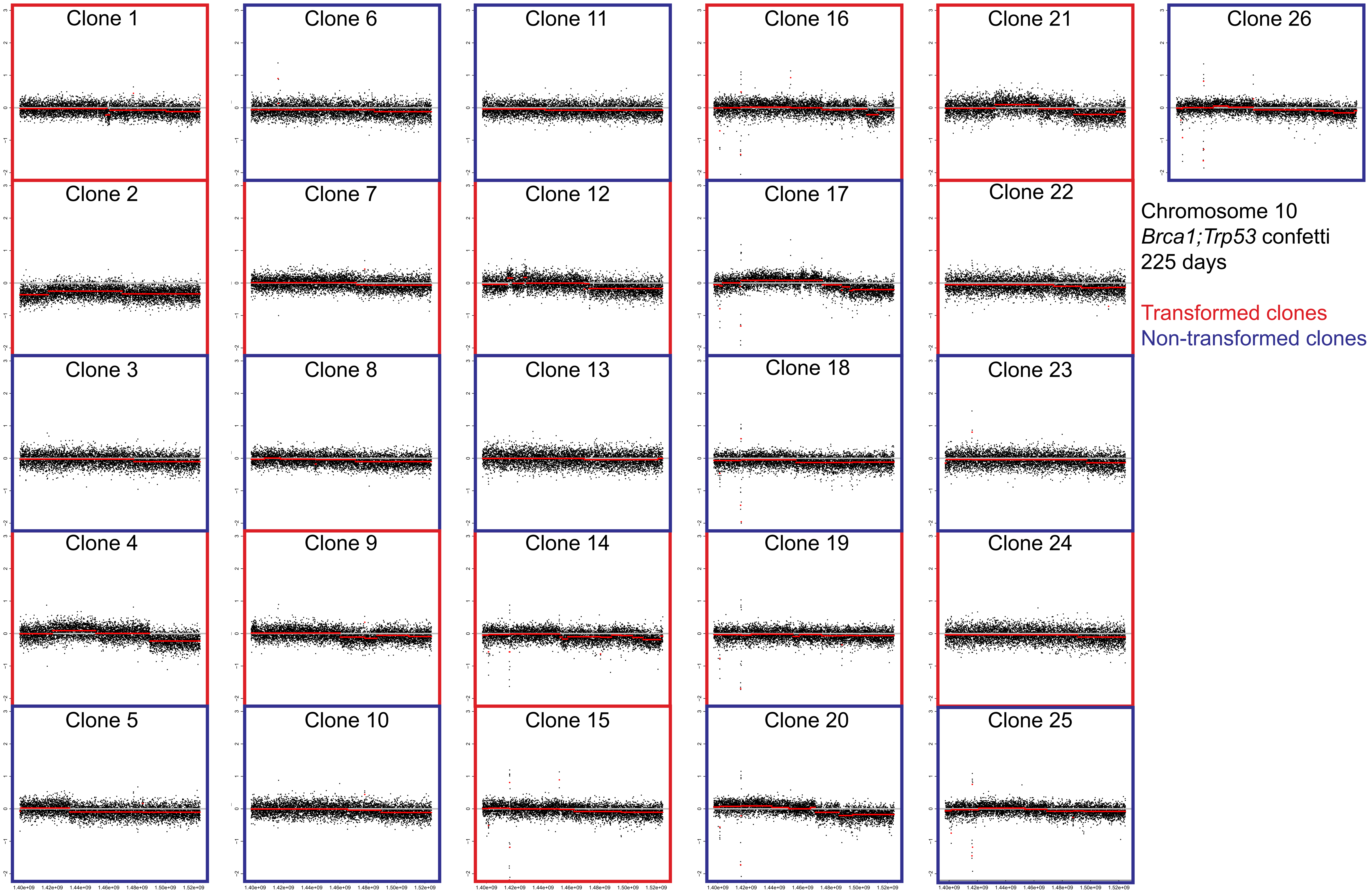
Clone 1
Clone 2
Clone 3
Clone 4
Clone 5
Clone 6
Clone 7
Clone 8
Clone 9
Clone 10
Clone 11
Clone 12
Clone 13
Clone 14
Clone 15
Clone 16
Clone 17
Clone 18
Clone 19
Clone 20
Clone 21
Clone 22
Clone 23
Clone 24
Clone 25
Clone 26
Chromosome 10
Brca1;Trp53 confetti
225 days
Transformed clones
Non-transformed clones

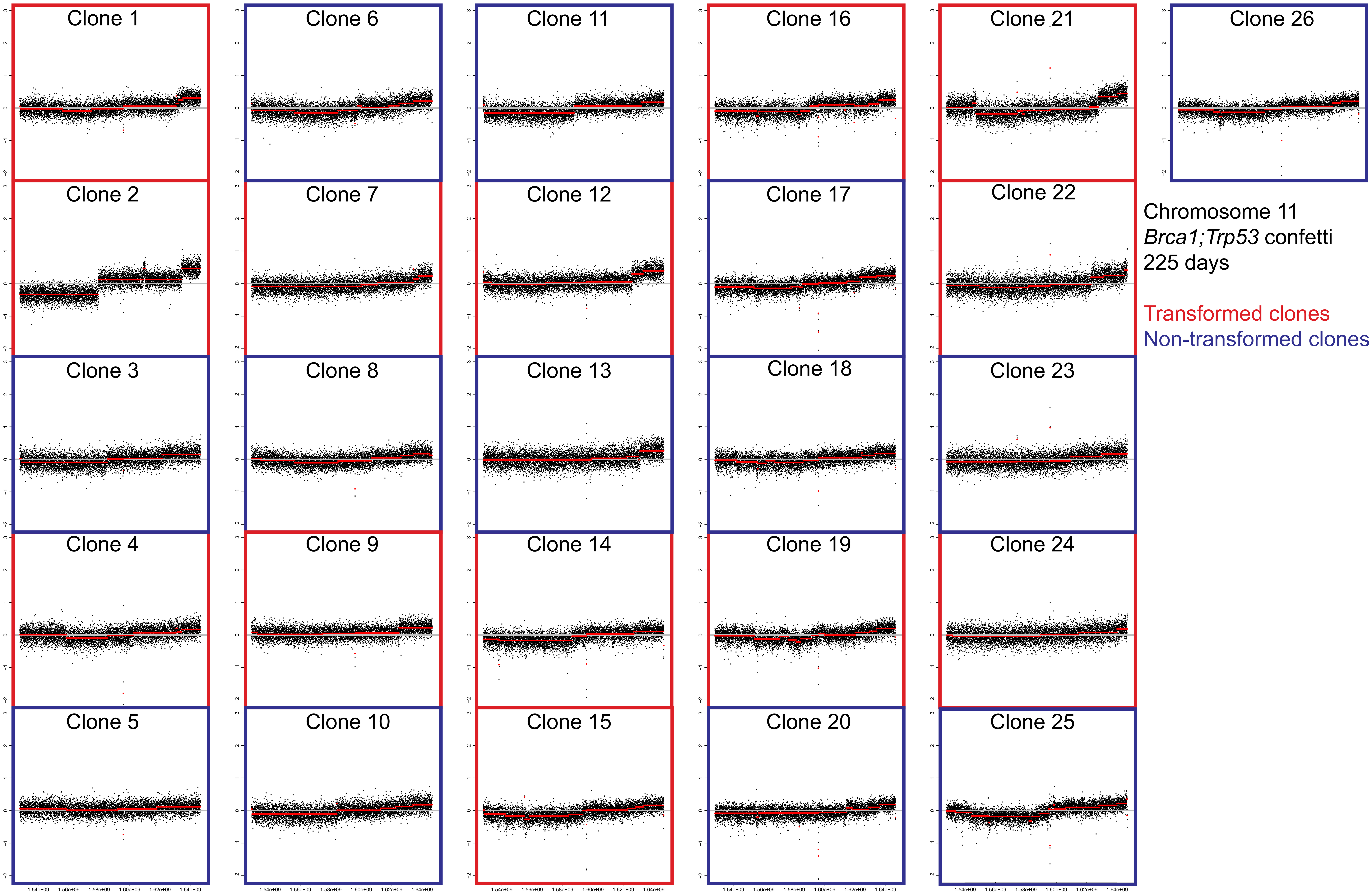
Clone 1
Clone 2
Clone 3
Clone 4
Clone 5
Clone 6
Clone 7
Clone 8
Clone 9
Clone 10
Clone 11
Clone 12
Clone 13
Clone 14
Clone 15
Clone 16
Clone 17
Clone 18
Clone 19
Clone 20
Clone 21
Clone 22
Clone 23
Clone 24
Clone 25
Clone 26
1.54e+09
1.56e+09
1.58e+09
1.60e+09
1.62e+09
1.64e+09
Chromosome 11
Brca1;Trp53 confetti
225 days
Transformed clones
Non-transformed clones

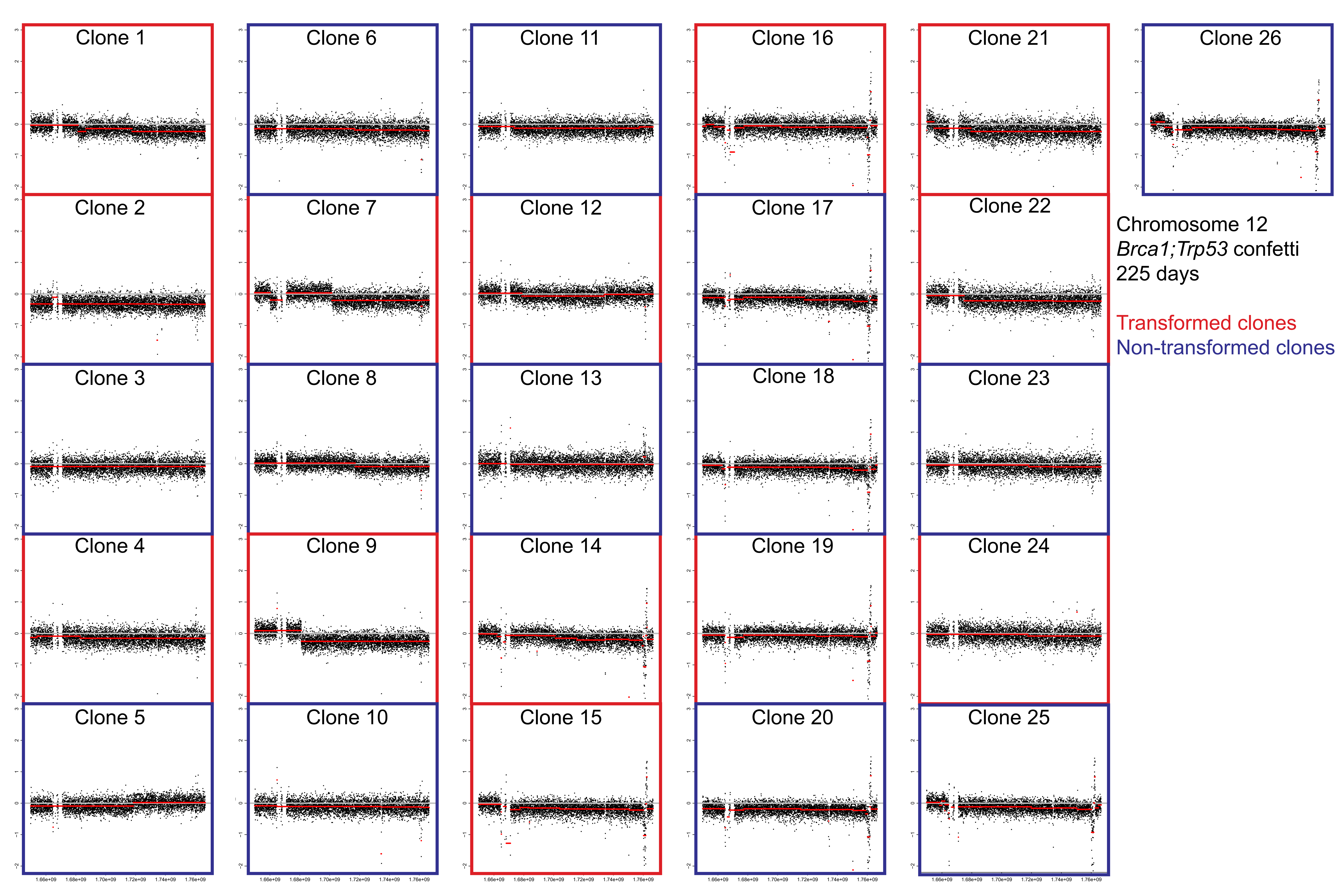
Clone 1
Clone 2
Clone 3
Clone 4
Clone 5
Clone 6
Clone 7
Clone 8
Clone 9
Clone 10
Clone 11
Clone 12
Clone 13
Clone 14
Clone 15
Clone 16
Clone 17
Clone 18
Clone 19
Clone 20
Clone 21
Clone 22
Clone 23
Clone 24
Clone 25
Clone 26
Chromosome 12
Brca1;Trp53 confetti
225 days
Transformed clones
Non-transformed clones
1.66e+09
1.68e+09
1.70e+09
1.72e+09
1.74e+09
1.76e+09

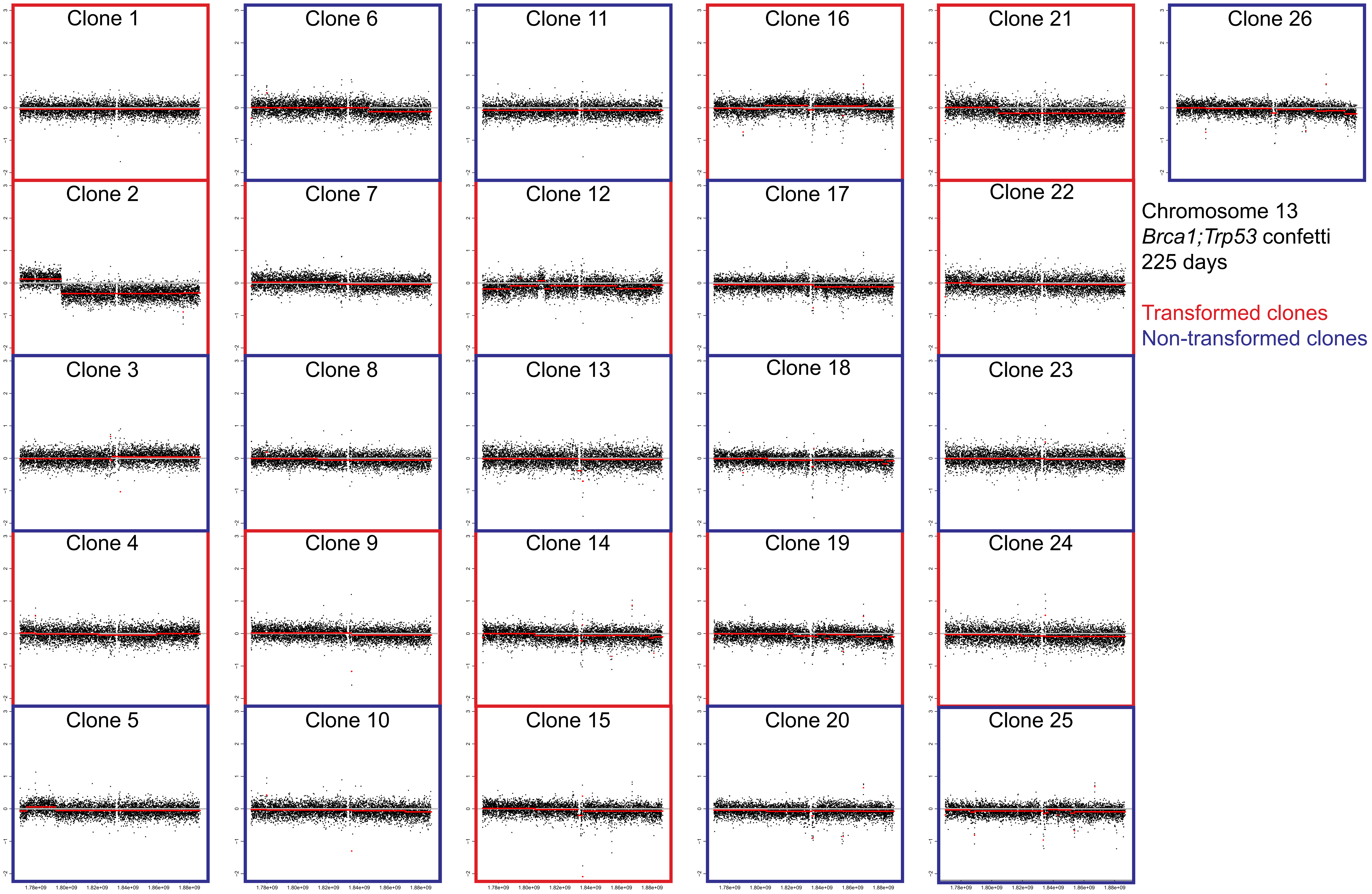

Clone 1
Clone 2
Clone 3
Clone 4
Clone 5
Clone 6
Clone 7
Clone 8
Clone 9
Clone 10
Clone 11
Clone 12
Clone 13
Clone 14
Clone 15
Clone 16
Clone 17
Clone 18
Clone 19
Clone 20
Clone 21
Clone 22
Clone 23
Clone 24
Clone 25
Clone 26
Chromosome 13
Brca1;Trp53 confetti
225 days
Transformed clones
Non-transformed clones
1.78e+09
1.80e+09
1.82e+09
1.84e+09
1.86e+09
1.88e+09

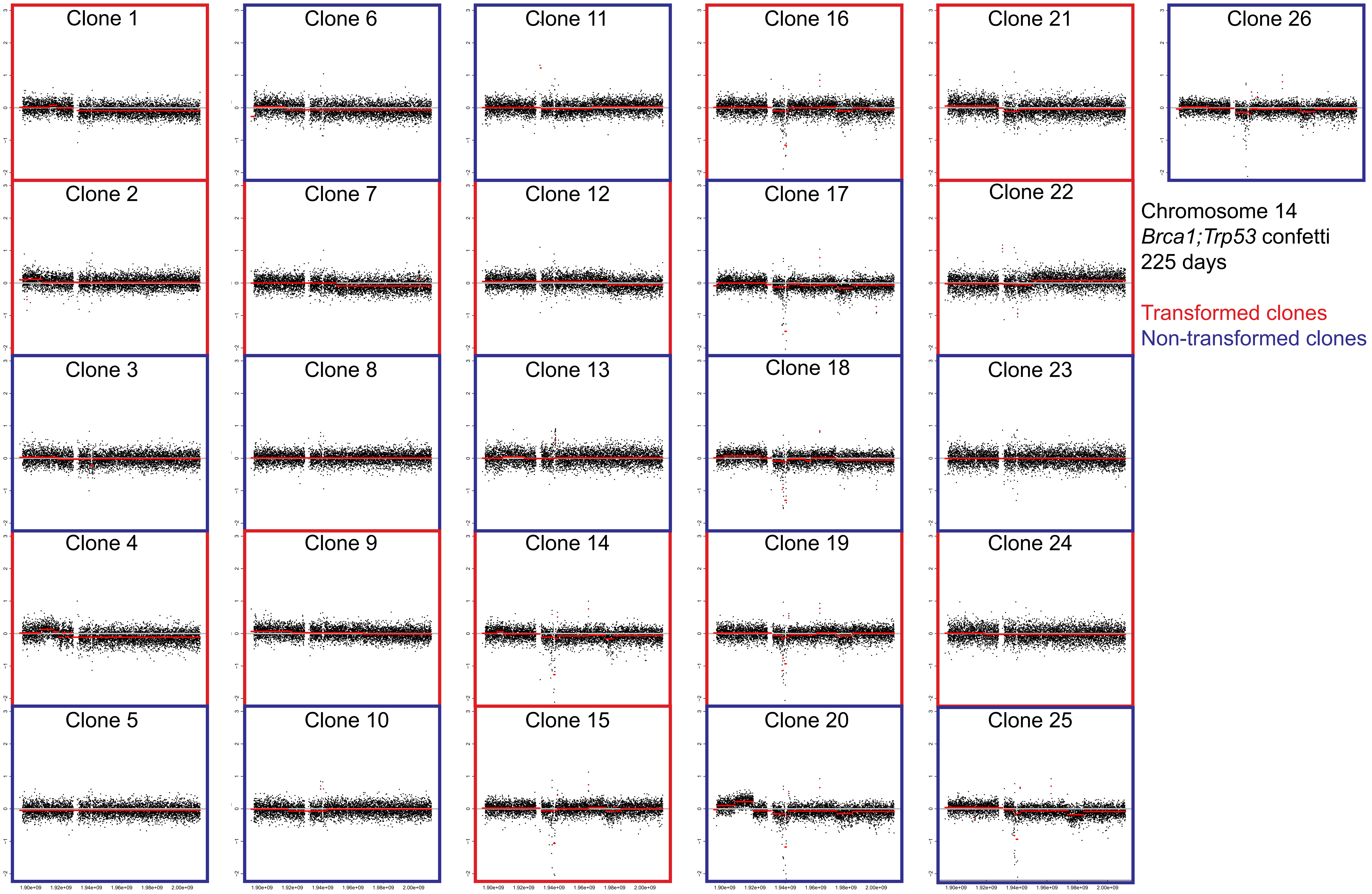

Clone 1
Clone 2
Clone 3
Clone 4
Clone 5
Clone 6
Clone 7
Clone 8
Clone 9
Clone 10
Clone 11
Clone 12
Clone 13
Clone 14
Clone 15
Clone 16
Clone 17
Clone 18
Clone 19
Clone 20
Clone 21
Clone 22
Clone 23
Clone 24
Clone 25
Clone 26
Chromosome 14
Brca1;Trp53 confetti
225 days
Transformed clones
Non-transformed clones

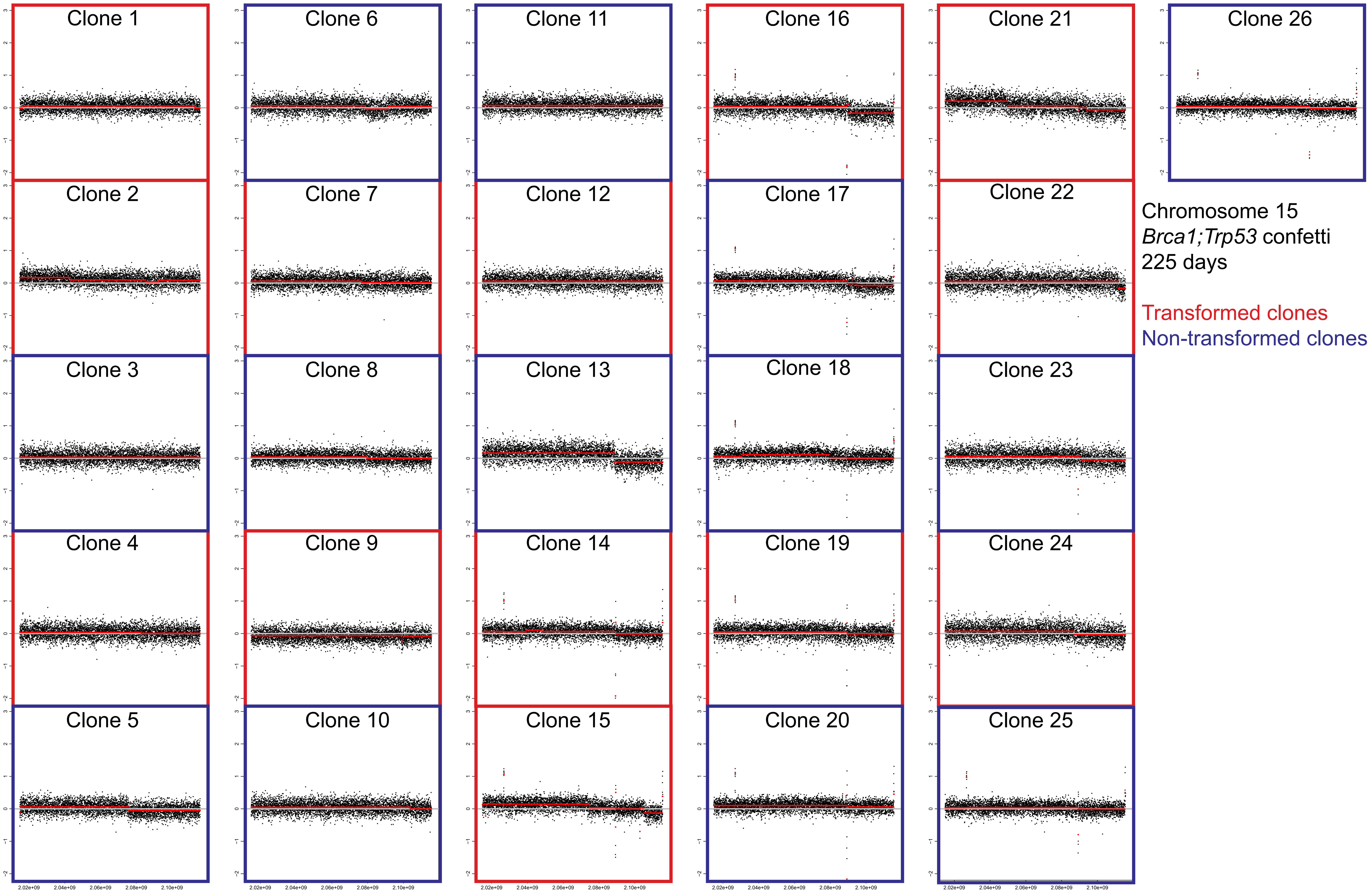

Clone 1
Clone 2
Clone 3
Clone 4
Clone 5
Clone 6
Clone 7
Clone 8
Clone 9
Clone 10
Clone 11
Clone 12
Clone 13
Clone 14
Clone 15
Clone 16
Clone 17
Clone 18
Clone 19
Clone 20
Clone 21
Clone 22
Clone 23
Clone 24
Clone 25
Clone 26
Chromosome 15
Brca1;Trp53 confetti
225 days
Transformed clones
Non-transformed clones
2.02e+09
2.04e+09
2.06e+09
2.08e+09
2.10e+09

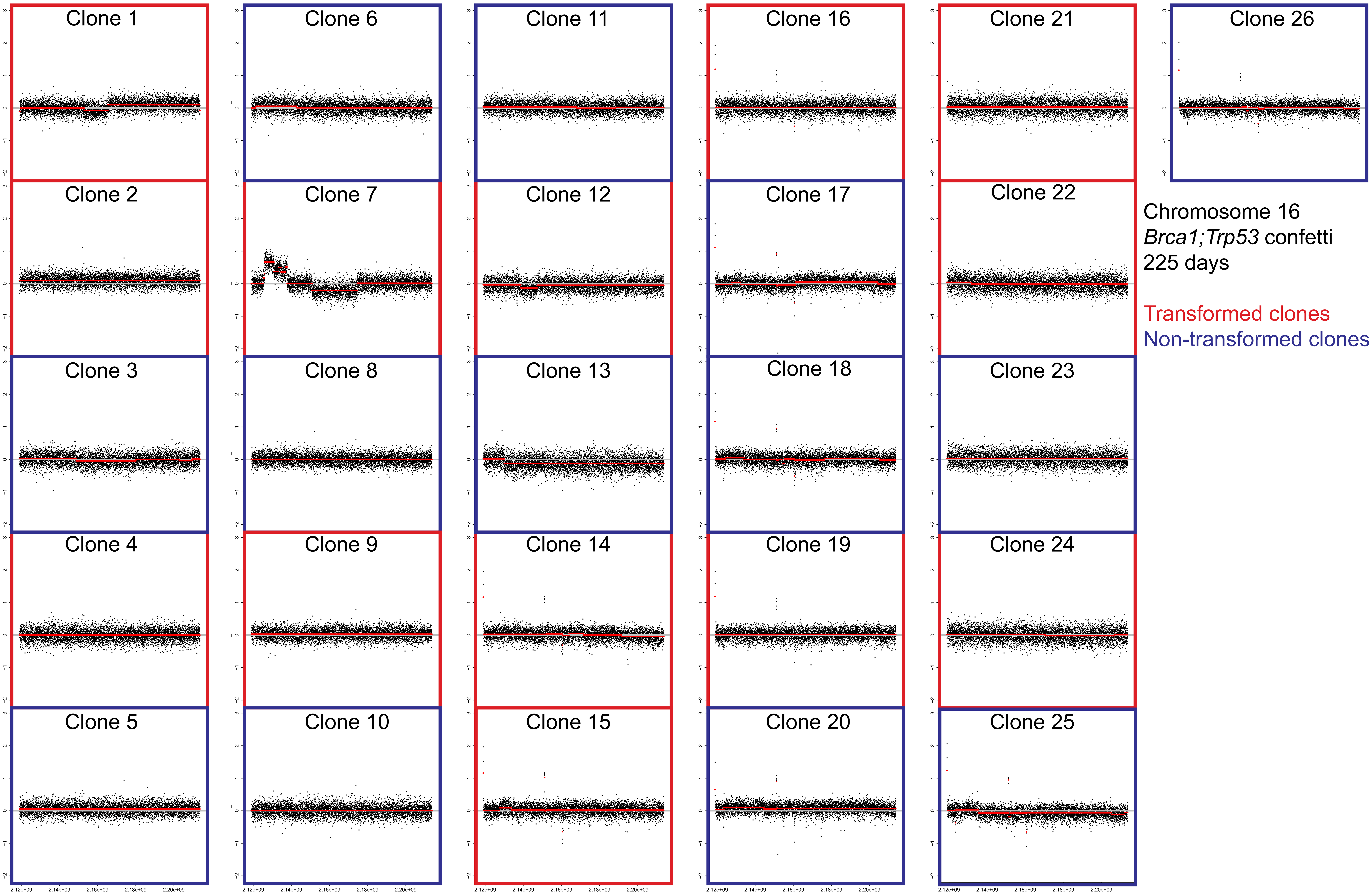
Clone 1
Clone 2
Clone 3
Clone 4
Clone 5
Clone 6
Clone 7
Clone 8
Clone 9
Clone 10
Clone 11
Clone 12
Clone 13
Clone 14
Clone 15
Clone 16
Clone 17
Clone 18
Clone 19
Clone 20
Clone 21
Clone 22
Clone 23
Clone 24
Clone 25
Clone 26
Chromosome 16
Brca1;Trp53 confetti
225 days
Transformed clones
Non-transformed clones

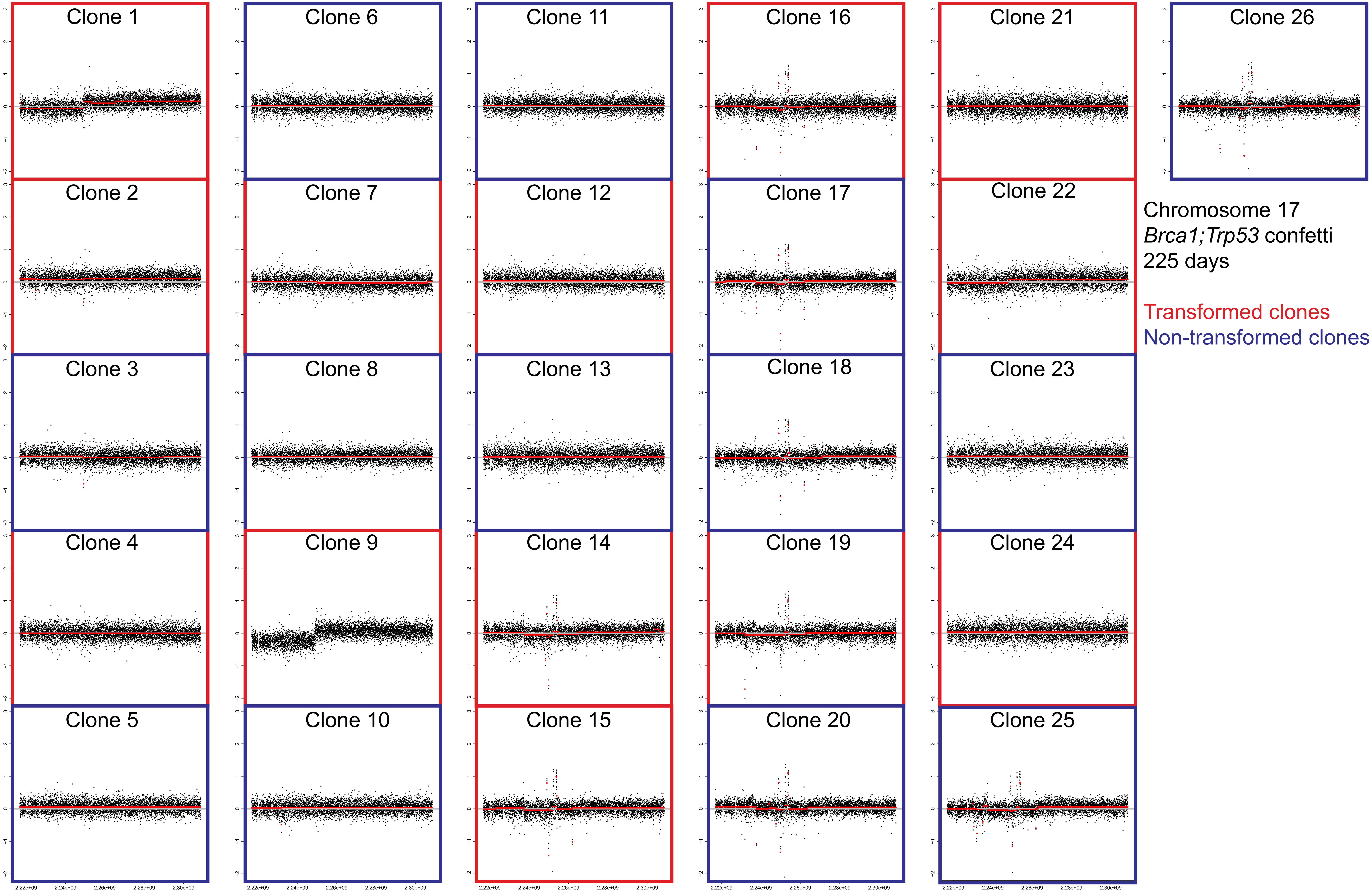
Clone 1
Clone 2
Clone 3
Clone 4
Clone 5
Clone 6
Clone 7
Clone 8
Clone 9
Clone 10
Clone 11
Clone 12
Clone 13
Clone 14
Clone 15
Clone 16
Clone 17
Clone 18
Clone 19
Clone 20
Clone 21
Clone 22
Clone 23
Clone 24
Clone 25
Clone 26
2.22e+09
2.24e+09
2.26e+09
2.28e+09
2.30e+09
Chromosome 17
Brca1;Trp53 confetti
225 days
Transformed clones
Non-transformed clones

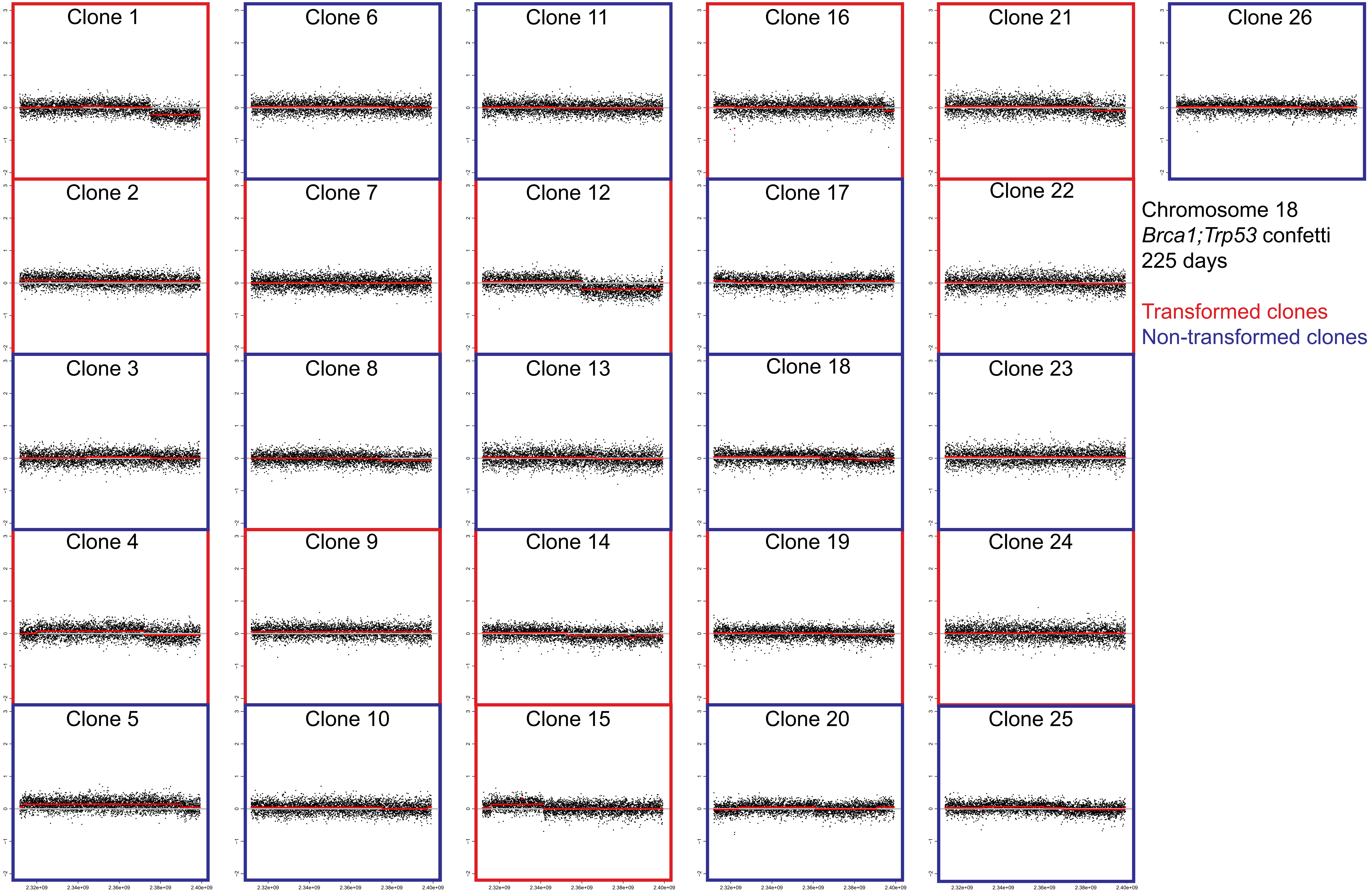
Clone 1
Clone 2
Clone 3
Clone 4
Clone 5
Clone 6
Clone 7
Clone 8
Clone 9
Clone 10
Clone 11
Clone 12
Clone 13
Clone 14
Clone 15
Clone 16
Clone 17
Clone 18
Clone 19
Clone 20
Clone 21
Clone 22
Clone 23
Clone 24
Clone 25
Clone 26
Chromosome 18
Brca1;Trp53 confetti
225 days
Transformed clones
Non-transformed clones

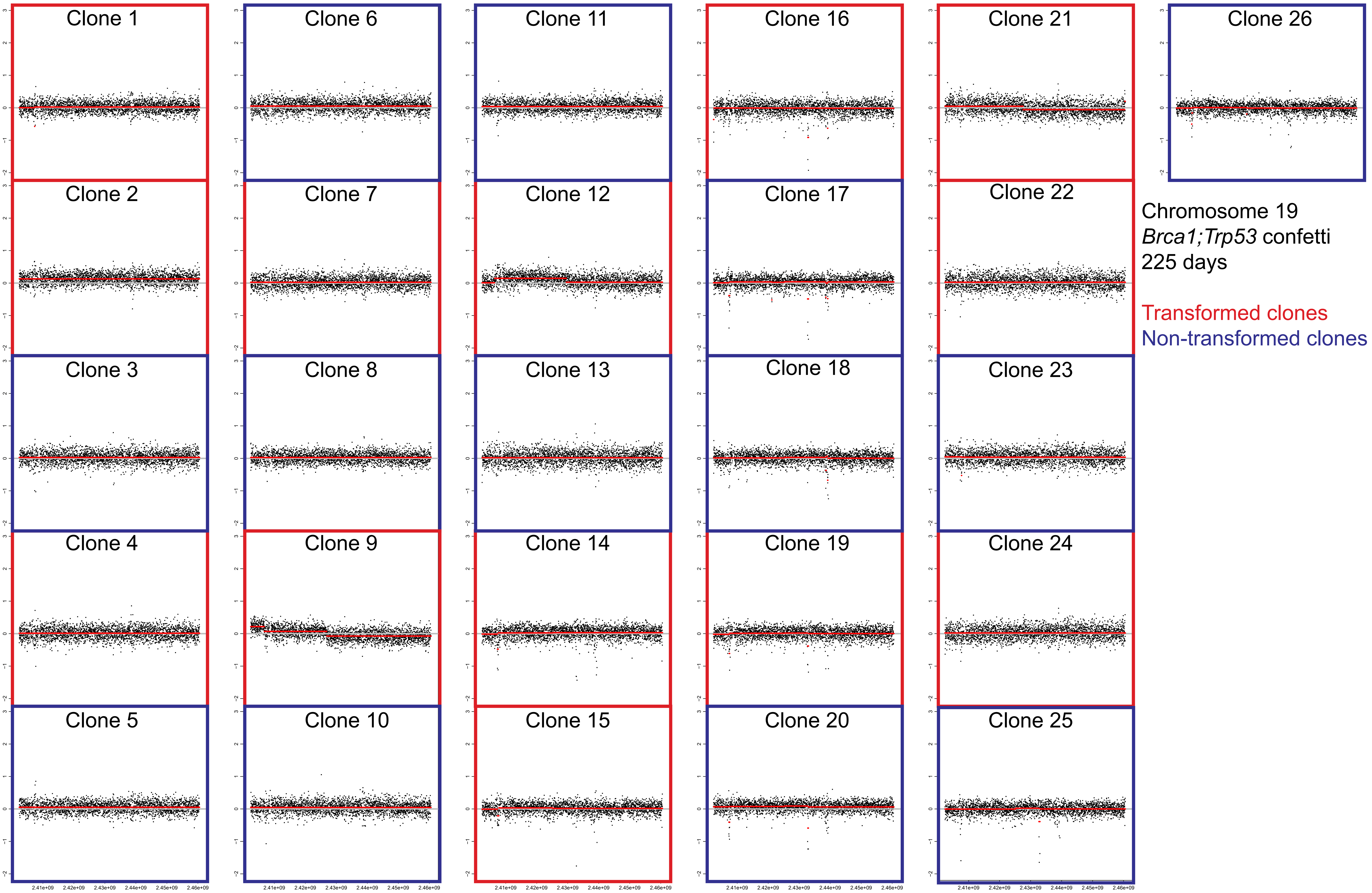
Clone 1
Clone 2
Clone 3
Clone 4
Clone 5
Clone 6
Clone 7
Clone 8
Clone 9
Clone 10
Clone 11
Clone 12
Clone 13
Clone 14
Clone 15
Clone 16
Clone 17
Clone 18
Clone 19
Clone 20
Clone 21
Clone 22
Clone 23
Clone 24
Clone 25
Clone 26
Chromosome 19
Brca1;Trp53 confetti
225 days
Transformed clones
Non-transformed clones

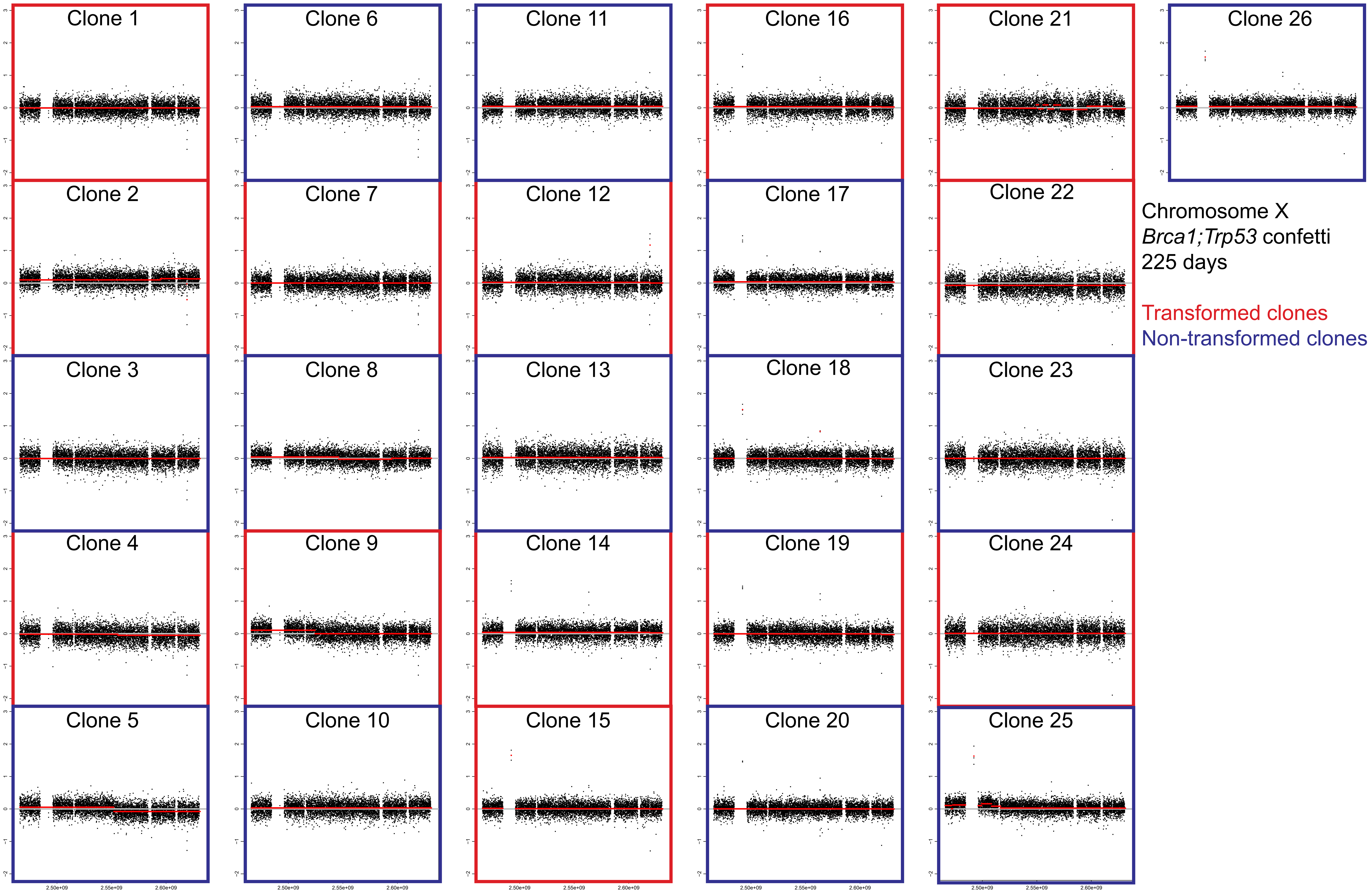
Clone 1
Clone 2
Clone 3
Clone 4
Clone 5
Clone 6
Clone 7
Clone 8
Clone 9
Clone 10
Clone 11
Clone 12
Clone 13
Clone 14
Clone 15
Clone 16
Clone 17
Clone 18
Clone 19
Clone 20
Clone 21
Clone 22
Clone 23
Clone 24
Clone 25
Clone 26
2.50e+09
2.55e+09
2.60e+09
Chromosome X
Brca1;Trp53 confetti
225 days
Transformed clones
Non-transformed clones
