# Supplementary material for: Mechanisms that clear mutations drive field cancerization in mammary tissue
Source: Nature. 2024 Sep 4;633(8028):198–206. doi: 10.1038/s41586-024-07882-3 (PMC11374684; doi:10.1038/s41586-024-07882-3)
Supplement: Supplementary file 5 — This file contains a Supplementary Note and Sections 1–5 including Figs. 1–10 and Tables. [file 41586_2024_7882_MOESM5_ESM.pdf]

Supplementary Note

In the following, we develop details of the theoretical and statistical analysis used to analyse the clone dynamics of the mammary gland epithelium in wildtype (WT) cells and those subject to non-neutral *Brca1/Trp53* mutations. We first identify and then make use of hallmark statistical features of the clone size data to abstract a minimal "zero-dimensional" model of the basal and luminal clone dynamics. We then test the integrity of the model against the experimental data using analytical properties of the average growth dynamics. To further develop the model of clone growth, we then consider how the inferred dynamics could emerge based on the spatial organisation and observed dynamics of the mammary gland ductal epithelium, using stochastic simulations to explore the clonal dynamics in this setting.

1. Statistical analysis of mammary gland clone size data

Beginning with the analysis of the WT confetti animals, a striking feature of the data is the extraordinary degree of clone size heterogeneity. At 225 days post-induction, some luminal clones could be found containing just a handful of cells, while others contained 100s and even 1000s of cells (Figure 2c). Therefore, we first questioned whether such clone size heterogeneity could derive, at least in part, from large scale mouse-to-mouse variability. When disaggregated across mice, there was indeed evidence for systematic variation between animals (Supplementary Figure 1), suggesting that the proliferative activity and turnover of the epithelium may be influenced by both systemic as well as local cues. Such behaviour is resonant with studies that report evidence for proliferative heterogeneity among animals, with the length and timing of the oestrous cycle, as well as the proliferative activity within glands, being affected by the pheromonal signals (Heijmans et al., 2024). Nevertheless, even accounting for such systematic variations, the clone size distribution showed evidence for large-scale heterogeneity even within individual animals. Therefore, to analyse the nature and origin of intra-mouse heterogeneity, we focussed on clone size data from individual animals.

In principle, heterogeneity of clone sizes within an individual animal could be associated with systematic local variation in the proliferative activity within individual glands. However, the apparent insensitivity of clone abundance and size to the branch level (Extended Data Fig. 7a-d) suggested that the origin of the clone size heterogeneity may not lie in such local influences. We therefore considered whether the dispersion of clone sizes could result from the outcome of chance fate decisions made by equipotent MaSCs. To address this question, we first considered whether there were statistical features of the clone size data that could constrain possible models. Previously, lineage tracing studies of cycling adult tissues have reported the emergence of statistical scaling behaviours of clone size, which have in turn implicated stochastic models of stem cell self-renewal (Chatzeli and Simons, 2020). In particular, when self-renewal relies on chance (i.e., probabilistic) stem cell loss and replacement of neighbouring cells, the size distribution of clones is predicted to converge over time onto a statistical scaling form, where the probability $C_n(t)$ of finding a clone at time t post-induction with a size n larger than a given multiple of the average $\bar{n}(t) \equiv \langle n \rangle$ becomes constant, independent of time. In this case, the cumulative distribution takes the scaling form $C_n(t) = \mathcal{C}(n/\bar{n}(t))$, where the scaling function $\mathcal{C}(x)$ is independent of time (Klein and Simons, 2011). The particular pattern of stochastic stem cell renewal is then encoded in the form of the scaling function, $\mathcal{C}(x)$. However, despite the observed ubiquity of such scaling behaviour in multiple adult epithelia, applied to the mammary gland clone data, we found that neither the luminal nor basal cumulative clone

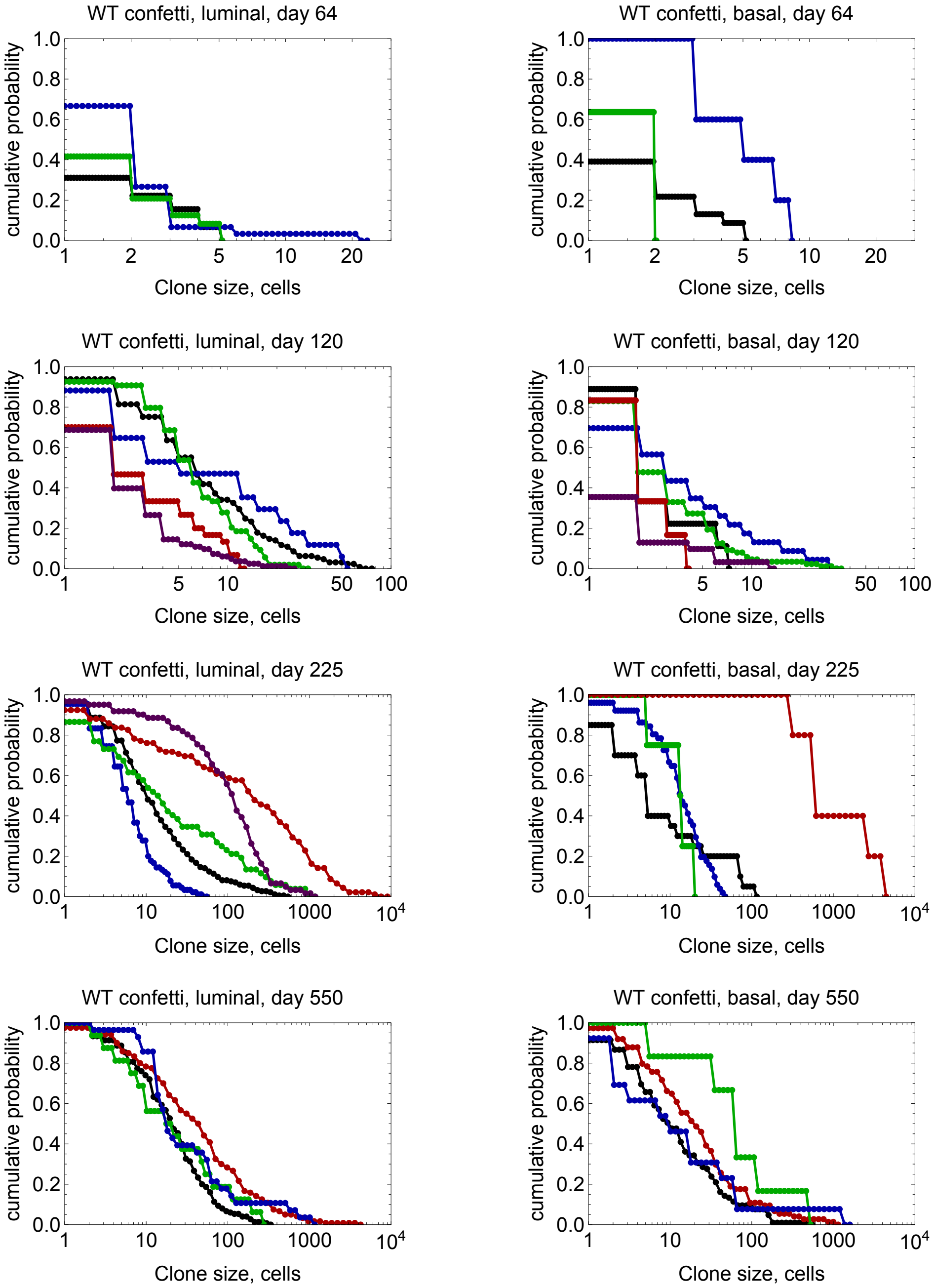

Figure 1: Cumulative distribution of luminal (left) and basal (right) WT confetti clone size showing the probability of finding a clone larger than the given size (log scale) across the given range of time points. Different animals are marked by distinct colours, with the same colour used to depict the luminal and basal clones from the same mouse at a given time point. Panels from Figure 3a and Extended Data Fig. 3c are drawn from the same data sets with colours chosen to match that shown here. Note that the level of basal cell induction was lower than luminal and in some cases no basal clones were found. Note also the very high degree of mouse-to-mouse variability, which becomes increasingly pronounced at the longer time points.

size distribution showed evidence for such a dependence (Supplementary Figure 2).

We therefore questioned whether there were different statistical characteristics of the clone size data that might imply an alternative pattern of MaSC renewal in the mammary gland epithelium. Notably, we found that, while the distribution of clone size did not show evidence for scaling, the statistics of the *logarithm* of clone size did. More specifically, if we define $C(w,t)$ as the probability of finding a clone with logarithm of size larger than $w \equiv \ln n$, where n denotes the number of cells in the clone, we found that both the luminal and basal clone size data converged rapidly onto the statistical scaling form (Supplementary Figure 3),

$$C(w,t) = \mathcal{C}\left(\frac{w-\mu_a(t)}{\sigma_a(t)}\right), \tag{1}$$

where $\mu_a(t) \equiv \langle \ln n \rangle$ represents the ensemble average of the logarithm of clone sizes for mouse a at chase time t and $\sigma_a(t) = \langle (\ln n - \mu_a(t))^2 \rangle^{1/2}$ denotes the corresponding standard deviation. Reliant on just two parameters, $\mu_a(t)$ and $\sigma_a(t)$, this striking result suggests that the broad heterogeneity of luminal and basal clone sizes is not associated with "engrained" heterogeneities in the fate of individual MaSCs (due, for example, to local spatial variations in niche factors), but follows from the stochastic fate outcome of renewing cells following a set of common statistical rules.

To understand the nature of these rules, we then questioned the form of the statistical scaling dependence, $\mathcal{C}(x)$. Notably, we found that the measured clone size distribution fit well with a log-normal dependence (Supplementary Figure 3), i.e., when rescaled according to Eq. (1), the clone size data collapse onto the scaling function $\mathcal{C}(x) = (1/2)\,\mathrm{erfc}(x/\sqrt{2})$, where $\mathrm{erfc}(x)$ denotes the complementary error function. Therefore, defining the $P(w,t)\,dw$ as the probability of finding a clone in the size interval between $n = e^w$ and e^{w+dw} cells at a time t post-induction, the luminal and basal clone size data fit well with the log-normal scaling distribution

$$P_a(w,t) \equiv \langle \delta(w-\ln n) \rangle = \frac{1}{\sqrt{2\pi}\sigma_a(t)} \exp\left[-\frac{(w-\mu_a(t))^2}{2\sigma_a^2(t)}\right]. \tag{2}$$

This robust and conserved feature of the experimental data showed that, across several orders of magnitude, the statistical distribution of luminal and basal clone size is characterised fully by just two parameters, the average $\mu_a(t)$ and variance $\sigma_a^2(t)$.

Based on this behaviour, we questioned how such a log-normal clone size dependence could arise. During the oestrous cycle, patches of ducts become proliferatively active, driving ductal outgrowths that then largely regress and remodel back towards the initial state (Extended Data Fig. 6a). We reasoned that this process was likely to effect a spatially coordinated response of ductal cells - including MaSCs - in the local neighbourhood, leading to a cooperative expansion or elimination of clones during the oestrous cycle. Note that this behaviour contrasts with the usual pattern of epithelial stem cell renewal where, in the course of turnover, cells compete neutrally with their neighbours for survival. Here, the expansion or loss of clones through ductal growth and regression is coordinated locally so that stem cells within an individual clone are likely to follow the same fate - expansion through cell duplication or loss. Therefore, based on this reasoning, we proposed that, for a given ductal region, the net change in the size of a clone after N rounds of cycle is given by

$$A_N = \prod_{i=1}^{N} a_i\,,$$

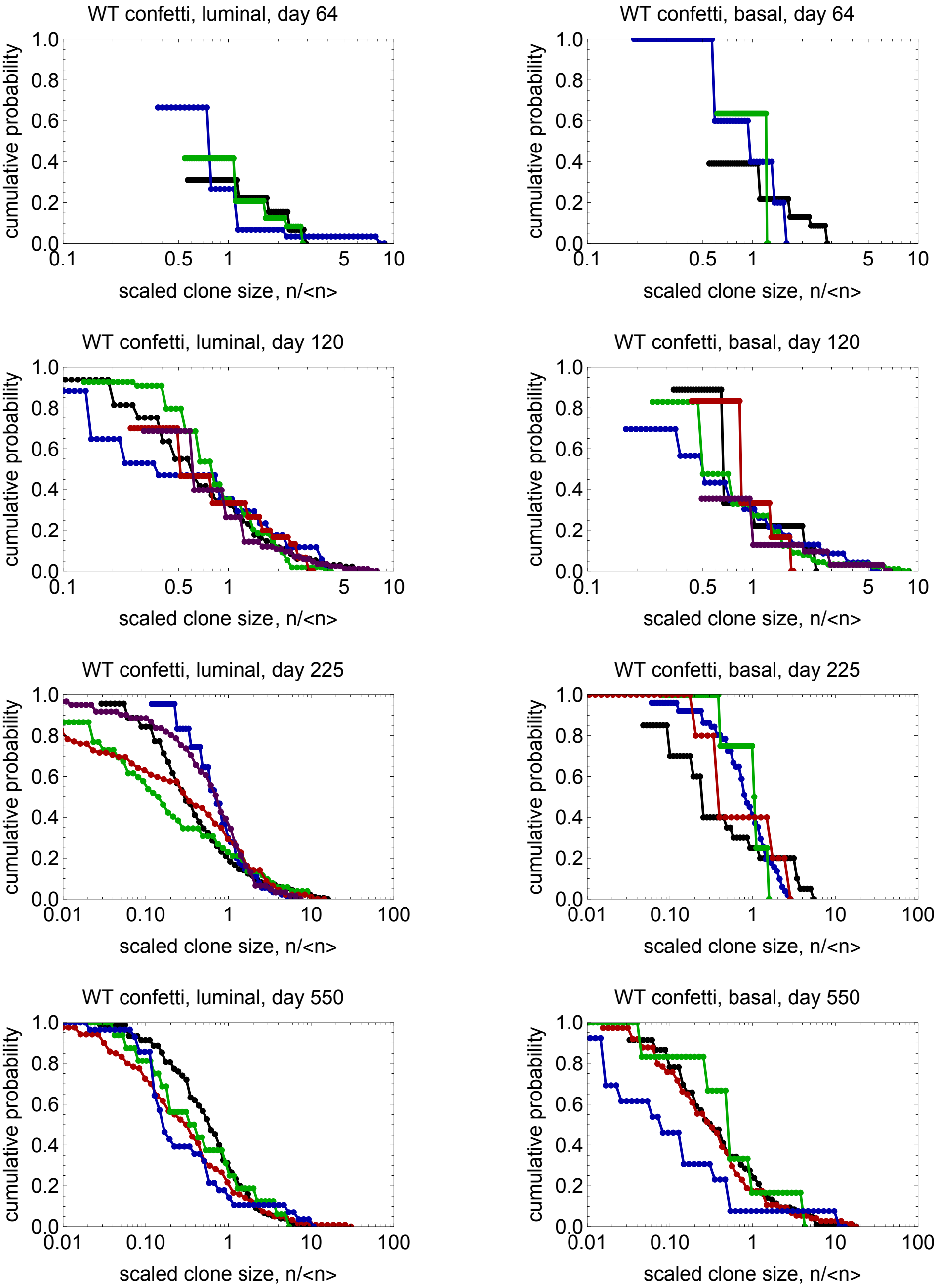

Figure 2: Cumulative size distribution of luminal (left) and basal (right) clones obtained from WT confetti animals across a range of time points plot as a function of the rescaled size $n/\langle n \rangle$. Different animals are marked by different colours, with the same colour used to depict the luminal and basal clones from the same mouse at a given time point. The raw data is shown in Supplementary Figure 1. Note that, when compared across time points and across mice within individual time points, the clone size data do not show evidence for data collapse under this form of linear scaling, as would be predicted by a model based on local stem cell loss and replacement.

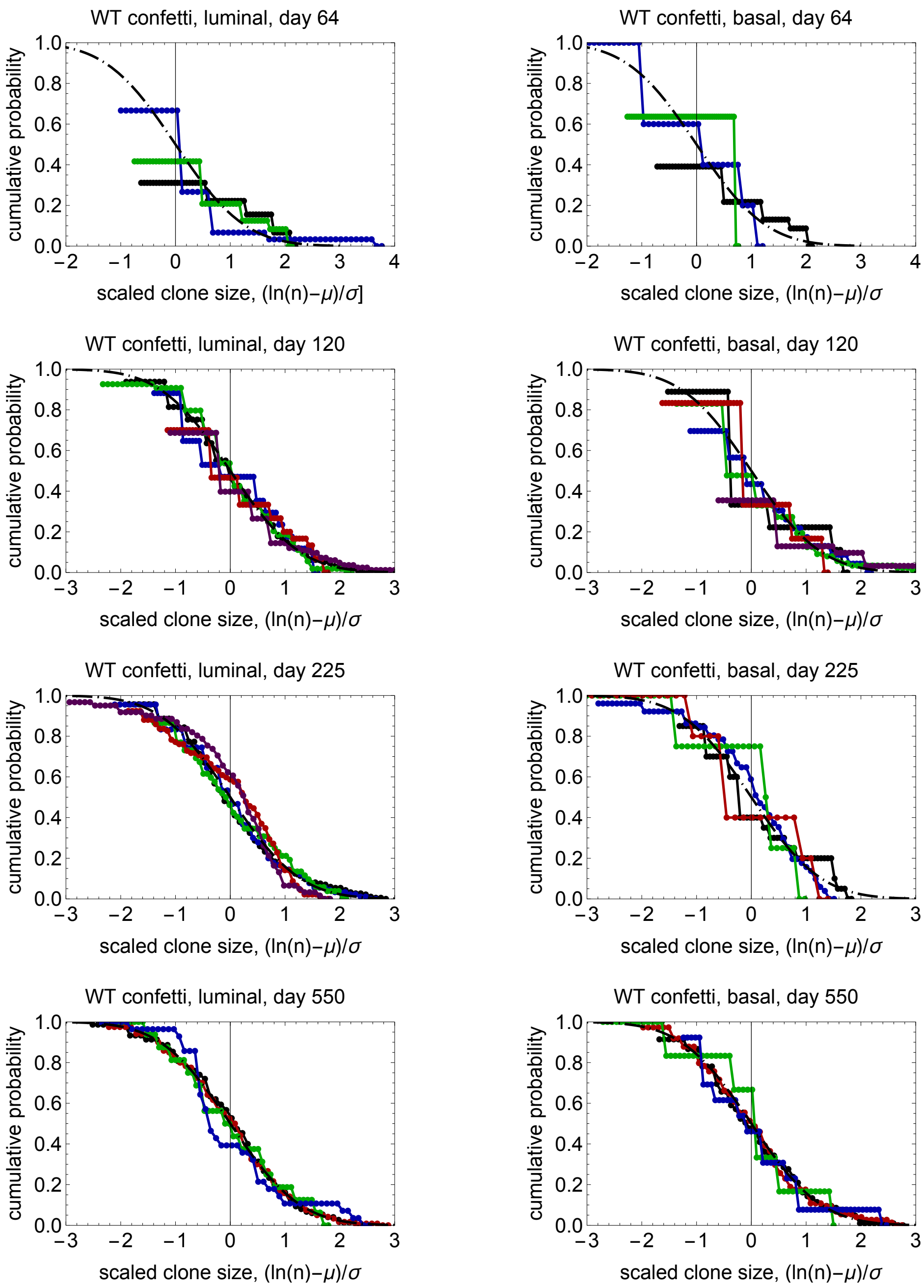

Figure 3: Cumulative size distribution of luminal (left) and basal (right) clones obtained from WT confetti animals across a range of time points as a function of the rescaled size $(\ln n - \mu_a(t))/\sigma_a(t)$, where $\mu_a(t) \equiv \langle \ln n \rangle$ represents the ensemble average of the logarithm of clone sizes for mouse a at chase time t and $\sigma_a^2(t) = \langle (\ln n - \mu_a(t))^2 \rangle$ denotes the corresponding variance. Different animals are marked by different colours, with the same colour used to depict the luminal and basal clones from the same mouse at a given time point. The raw data is shown in Supplementary Figure 1. Note that, when compared across time points and across mice within individual time points, under rescaling the clone size data collapse onto a log-normal dependence with the scaling function $\mathcal{C}(x) = (1/2)\,\mathrm{erfc}(x/\sqrt{2})$, where $\mathrm{erfc}(x)$ denotes the complementary error function (dashed). For the statistical tests of convergence to log-normality, we refer to the Supplementary Theory text where KDL values are given.

where a_i denotes the fractional change in clone size at time step (or, more accurately, oestrous cycle number) i. For now, it is assumed that the ducts comprise only renewing MaSCs. Moreover, since different ductal compartments are found to be lineage restricted (see main text), here we focus for simplicity on one of the constituent stem cell populations. The total number of cycles N then increases over time in proportion to the rate of the oestrous cycle, $1/t_{\rm e}$, which is estimated to be around once per 4-7 days in mouse (but may change significantly with age and environmental conditions).

How should the amplification factors a_i be chosen? Previous studies based on the incorporation of thymidine analogues suggest that entry into cycle is a comparatively rare event in which local domains of ductal cells become active while the vast majority of ductal cells remain largely quiescent (Giraddi et al., 2015). Here, we proposed that these events occur stochastically so that the amplification factors, a_i, are random variables. Since the process of amplification and regression acts multiplicatively, the logarithm $\ln A_N = \sum_i \ln a_i$ will, by the central limit theorem, become normally distributed over time (Huxley, 1932). This will be true regardless of the nature of the statistical distribution of a_i, providing that it is not pathological, providing a heuristic explanation of the emergence of the observed log-normal clone size dependence.

To test the validity of this hypothesis in the context of mammary clone dynamics, we turned to consider how such behaviour would translate into the growth characteristics of clone sizes and their survival probabilities. To develop this programme, it was useful to consider a concrete realisation of the stochastic process of tissue turnover during the oestrous cycle, which allowed us to also gain analytical insights into dependencies on model parameters. In this case, we considered as a phenomenological theory in which the amplification parameters are chosen as

$$a_i = \begin{cases} 1 & \text{Pr.}\,(1-q) \\ 0 & \text{Pr.}\,pq \\ 2^{\alpha_i} & \text{Pr.}\,(1-p)q \end{cases} \quad , \qquad (3)$$

where q represents the probability that a surviving clone becomes proliferatively active during a given oestrous cycle, while p denotes the probability that, once activated, a clone is altogether lost through chance extinction following regression and remodelling. The amplification parameters α are drawn at random from a statistical distribution $f(\alpha)$ where, for simplicity, we chose $f(\alpha) = \frac{1}{\theta} e^{-\alpha/\theta}$ with 2^θ representing the typical scale of clone expansion. In this case, since the distribution $f(\alpha)$ is asymmetric, with $\alpha \geq 0$, surviving stem cell clones always grow in size or stay unchanged during a single cycle. However, by adjusting the magnitude of the probability p, such a persistent increase in the average size of surviving clones can be compensated by stochastic clonal loss to enforce the condition of tissue homeostasis.

With these definitions, we then considered how they translate into the probability distribution of the logarthim of clone size, $P(w) \equiv \langle \delta(w - \ln A_N) \rangle$. However, it is important to note that, when the clone extinction probability, p, is non-zero, many clones will be of size zero, i.e., those that have become activated and altogether lost. Yet, the frequency of these "extinct" clones cannot be estimated directly from experimental observation. It therefore makes sense to consider the ensemble of visible (i.e., surviving) clones, defined as those that contain at least one stem cell. In the current model, the frequency of such clones, as a fraction of the total induced stem cell population, will decay over time as

$$P_{\rm surv}(t) = (1-pq)^{N(t)} \simeq e^{-pqN(t)} \, ,$$

where $N(t)$ represents the number of oestrous cycles after time t. Then, to proceed, we focused

on the statistical distribution of surviving clone sizes, $\ln A_N$, later adjusting averages where necessary to account for ongoing clonal loss.

For the ensemble of surviving clones, the distribution of clone size is then given by

$$\begin{aligned}\langle\delta(w-\ln A_N)\rangle &= \left\langle\int_{-\infty}^{\infty}\frac{d\phi}{2\pi}\,e^{i\phi(w-\ln A_N)}\right\rangle\\ &= \int_{-\infty}^{\infty}\frac{d\phi}{2\pi}\,e^{i\phi w}\left[\frac{1}{1-pq}\left(1-q+q(1-p)\int_0^{\infty}d\alpha f(\alpha)\,e^{-i\phi\,\alpha\ln 2}\right)\right]^N ,\end{aligned}$$

where $N=N(t)$ represents the total number of cycles and $\langle\cdots\rangle=\int d\alpha\cdots f(\alpha)$ denotes the average over the distribution of α. Using the identity,

$$\frac{1}{\theta}\int_0^{\infty}d\alpha\,e^{-\alpha/\theta-i\phi\,\alpha\ln 2}=\frac{1}{1+i\theta\phi\ln 2}=1-i\theta\phi\ln 2-\theta^2\phi^2\ln^2 2+O(\phi^3)\,,$$

we thus obtain

$$\langle\delta(w-\ln A_N)\rangle\simeq\int_{-\infty}^{\infty}\frac{d\phi}{2\pi}\,e^{i\phi w}\left[1-\tilde{q}\left(i\theta\phi\ln 2+\theta^2\phi^2\ln^2 2\right)\right]^N ,$$

where, to simplify the equations, we have introduced the composite parameter

$$\tilde{q}=\frac{q(1-p)}{(1-pq)}\,. \tag{4}$$

Note that when there is no clone loss, $p=0$, $\tilde{q}$ is simply the activation probability q. Then, taking $\tilde{q}\ll 1$ (consistent with the limit in which, for the majority of oestrous cycles, stem cells remain fully quiescent with $a_i=1$), the integral is dominated by small ϕ and

$$\begin{aligned}\langle\delta(w-\ln A_N)\rangle &\simeq \int_{-\infty}^{\infty}\frac{d\phi}{2\pi}\,e^{i\phi w}\exp\left[-N\tilde{q}\left(i\theta\phi\ln 2+\theta^2\phi^2\ln^2 2\right)\right]\\ &\simeq \int_{-\infty}^{\infty}\frac{d\phi}{2\pi}\,e^{i\phi(w-N\tilde{q}\theta\ln 2)}\exp\left[-N\tilde{q}(\theta\phi\ln 2)^2\right]\\ &= \frac{1}{2\theta\ln 2\sqrt{\pi N\tilde{q}}}\exp\left[-\frac{(w-N\tilde{q}\theta\ln 2)^2}{4N\tilde{q}\theta^2\ln^2 2}\right].\end{aligned}$$

From this result it follows that $\ln A_N$ is normally distributed with average $\mu_N\equiv\langle\ln A_N\rangle=N\tilde{q}\theta\ln 2$ and variance

$$\sigma_N^2\equiv\langle(\ln A_N-\mu_N)^2\rangle=2N\tilde{q}(\theta\ln 2)^2\,. \tag{5}$$

As noted above, this result is simply a manifestation of the central limit theorem. Over the long term, a different choice of the statistical distribution $f(\alpha)$ would also lead to a log-normal clone size dependence, albeit with the average and variance revised according to the chosen model. However, if the statistical rules of clone growth remain invariant across cycles, the linear dependence of the average and variance on time (cycle number) N would be conserved.

Before considering the predictions of the theory, we must first consider how these results relate to the observed clone size. Based on an initial precipitous drop in surviving clone number (Figure 2d), we reasoned that not all ductal cells have long-term renewal potential, but belong

instead to a transit-amplifying or non-dividing pool. To restore tissue during bouts of growth and regression, these shorter-lived cells must be replenished by stem cells. Therefore, in the long term, each stem cell would, on average, be associated with some $n_{\rm TA}$ transit-amplifying or differentiated cell progenies that, together with the renewing cell, comprise the MaSC-descendant unit. The total clone size would, therefore, be given by $n \simeq A_N(1+n_{\rm TA})$ with

$$P(w,t) \equiv \langle \delta(w - \ln n)\rangle = \frac{1}{\sqrt{2\pi}\sigma_N} \exp\left[-\frac{(w-\mu_N)^2}{2\sigma_N^2}\right], \tag{6}$$

where both the variance σ_N^2, Eq. (5), and average

$$\mu_N \equiv \langle \ln n\rangle = N\tilde{q}\theta \ln 2 + \ln(1+n_{\rm TA}) \tag{7}$$

grow linearly with cycle number, N. From this result, it follows that the average surviving clone size

$$\langle n\rangle = \int_0^\infty dw\, e^w \,\langle \delta(w-\ln n)\rangle \simeq \exp\left[\frac{\sigma_N^2}{2} + \mu_N\right]$$

increases exponentially fast with cycle number, consistent with the seemingly dramatic increase in observed clone sizes.

When considering the change in the total fraction of labelled cells over time, defined as the product of the average clone size with the clonal survival probability, any increase in average surviving clone size has to be weighed against net clonal loss, i.e., in homeostasis, the expansion of surviving clones occurs at the expense of the loss of neighbouring clones. Therefore, to obtain the true total fraction of labelled cells, we must renormalise the average size of surviving clones $\langle n\rangle$ with their survival probability $P_{\rm surv} = e^{-pqN}$. Altogether, the average number of labelled cells is therefore given by

$$\langle n\rangle e^{-pqN} = (n_{\rm TA}+1)e^{\kappa N} \tag{8}$$

where $\kappa = \tilde{q}(\theta\ln 2)^2 + \tilde{q}\theta\ln 2 - pq$. This means that, to achieve homeostasis, viz. $\kappa = 0$, the clone extinction probability must be chosen such that

$$p = p_{\rm homeo} \equiv \frac{1}{2q}(1+\beta)\left[1 - \sqrt{1 - \frac{4\beta q}{(1+\beta)^2}}\right], \tag{9}$$

where for brevity we have introduced the parameter $\beta = \theta\ln 2(1+\theta\ln 2)$. From this result, it follows that the average size of surviving clones grows exponentially as

$$\langle n\rangle_{\rm surv} = \frac{(n_{\rm TA}+1)}{P_{\rm surv}} = (n_{\rm TA}+1)e^{\tilde{q}\beta N(t)}. \tag{10}$$

Finally, from these results, one can determine the fraction of stem cell clones that, by chance, remain quiescent over time. Since, in the current model, clonal loss can only occur following activation, among the population of surviving clones, the fraction that have not yet entered cycle after N rounds of oestrous cycle is thus given by

$$P_{\rm single} = \frac{(1-q)^N}{P_{\rm surv}} \simeq e^{-q(1-p)N}, \tag{11}$$

decaying exponentially with cycle number.

Non-neutral dynamics

Before turning to the experimental data, it is necessary to consider how these results would be modified in the context of non-neutral competition. In particular, how would clones expand under the influence of a mutation that imposed a competitive advantage over neighbouring wild-type clones? For example, how would clones expand if they experienced the same rate of "activation", viz. probability q, and the same propensity for collective cell loss during the regression phase, viz. probability p, as wild-type, but with an elevated expansion rate $\theta_{\rm mut}$? In this case, the theory predicts the emergence of the same log-normal clone size dependence, but with an average (7) and variance (5) modified according to the increase in θ. Accordingly, the clonal expansion and loss become imbalanced leading to a net exponential increase in the number of labelled cells over time,

$$(n_{\rm TA}+1)e^{\kappa_{\rm mut}N}$$

where $\kappa_{\rm mut}=[(\theta_{\rm mut}+\theta)\ln^2 2+1](\theta_{\rm mut}-\theta)\tilde{q}$. However, importantly, although the increase in surviving clone size would be enhanced in magnitude over wild-type, the increase would still scale linearly with cycle number.

Alternatively, mutant cells could gain competitive advantage not by experiencing an intrinsic growth advantage during the expansion phase, but through resistance to apoptosis during the regression phase. In this case, one would obtain the same log-normal size dependence and linear growth characteristic, but with the extinction probability not be constrained to the homeostatic value $p_{\rm homeo}$, leading to the expansion and colonization of tissue by mutant cells according to Eq. (8).

This completes the theoretical analysis of the model dynamics. Within the framework of the minimal zero-dimensional model, we have found that the emergent log-normal scaling dependence of clone size can be explained through a process of collective cell loss and expansion through the remodelling of tissue that takes place during the oestrous cycle. Indeed, referring to the main text, time-lapse imaging of the mammary gland epithelium confirms this mechanism of epithelial expansion and regression (Figure 4). However, these results show that, instead of perfectly restoring the tissue back to its original state, under rounds of oestrous cycle, many tertiary branches are able to slowly grow and expand. At the level of the clone dynamics, such effects would mimic the influence of non-neutral mutations (viz. $\kappa>0$), while preserving the overall log-normal clone size dependence. To further strengthen confidence in the phenomenological model, we turn now to consider whether the theory can predict the average clone growth characteristics.

2. Experimental fits: WT confetti mutant

To compare the predictions of the zero-dimensional model to the experimental data, we begin with the clonal data from the *R26-CreERT2;R26*-confetti labelling system, turning later to consider the *Brac1;Trp53* mutant.

Distribution of clone size: First, as evident from Supplementary Figure 3, after rescaling both luminal and basal clone sizes fit well with a log-normal clone size dependence. To assess

the goodness of this zero-parameter fit, we followed the approach of Rompolas et al.,[1] making use of a statistical test based on the Kullbeck-Leibler Divergence (KLD). Here, for each time point, we calculated the parameter

$$\mathrm{KLD} = \int_{-\infty}^{\infty} dx P(x) \ln\left(\frac{P(x)}{Q(x)}\right)$$

where, with $x = (\ln x - \mu(t))/\sigma(t)$, $P(x)$ denotes the experimentally inferred probability distribution of clone size (with a Gaussian smoothing) while $Q(x) = \exp(-x^2/2)/\sqrt{2\pi}$ denotes the predicted Gaussian distribution consistent with the log-normal dependence. For a perfect fit of the data to a log-normal clone size dependence, KLD = 0. From a comparison to the data shown in Supplementary Figure 3, the KDL values of luminal (left) and basal (right) clones take the values:

time (d)	mouse id.	clones	KDL val.
64	black	45	0.72
	blue	30	0.25
	green	24	0.57
120	black	129	0.012
	blue	17	0.17
	green	54	0.087
	red	30	0.22
	purple	83	0.10
225	black	185	0.029
	blue	90	0.014
	green	52	0.054
	red	92	0.12
	purple	61	0.13
550	black	150	0.0092
	blue	28	0.17
	green	16	0.061
	red	120	0.0043

time (d)	mouse id.	clones	KDL val.
64	black	23	0.60
	blue	5	0.23
	green	11	1.24
120	black	9	0.47
	blue	23	0.12
	green	88	0.077
	red	6	0.44
	purple	31	0.43
225	black	20	0.15
	blue	51	0.090
	green	4	0.30
	red	5	0.090
550	black	105	0.020
	blue	13	0.19
	green	6	0.21
	red	74	0.018

Here, the colours refer to the data shown in Supplementary Figure 3. At the earlier time points, the integer discretisation of cell number reduces the quality of the fit, as evidence by the higher KDL values. However, inspection of the cumulative clone size distributions shows that, even for these cases, the profile of the data fits respectably with a log-normal size dependence. Later, we will discuss and interpret specific outliers.

Statistical correlation between clone size average and variance: Although these results show that the experimental data is consistent with a log-normal clone size scaling dependence, by itself, this observation does not identify a specific model. Any model that gives rise to log-normal scaling would, by definition, be consistent with the data. Therefore, to gain confidence in the stochastic clone loss and replacement model, we turned to consider further predictions for the average growth dynamics. However, a key challenge in tracking the temproal growth characteristics is that the mammary epithelium in individual animals undergo highly variable degrees of turnover, as evidenced by the large-scale mouse-to-mouse variability (Supplementary

[1] P. Rompolas et al., *Spatiotemporal coordination of stem cell commitment during epidermal homeostasis*, Science **352**, 1471-1474 (2016).

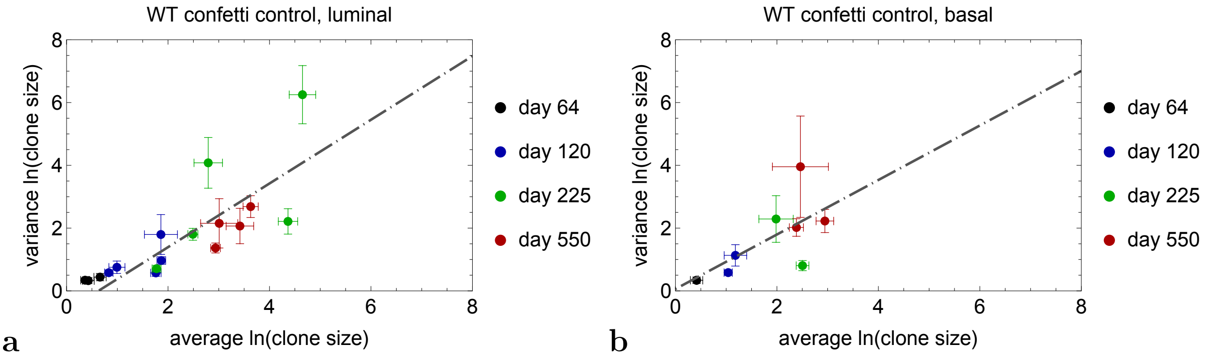

Figure 4: **a,b,** Plot of the variance against the average of the log of luminal (a) and basal (b) clone size of WT confetti mice. Colours denote chase times. Each dot denotes an individual mouse with bars denoting the standard error. The line shows a fit of the data to a linear dependence, as predicted by the minimal zero-dimensional model (12). For details of the fit values and goodness of fit, we refer to Supplementary Theory.

Figure 1). Therefore, to begin we first looked for statistical characteristics that could be made independent of the age of the clone. In this context, a feature of the predicted growth dynamics is that both the average and variance of the logarithm of clone size scales linearly with the accumulated oestrous cycle number post-induction, as shown in Eqs. (5,7). Specifically, within the framework of the minimal model, it therefore follows that

$$\sigma_N^2 = 2\theta \ln 2 \left(\mu_N - \ln\left(1 + n_{\mathrm{TA}}\right)\right) . \qquad (12)$$

This means that, if the variance σ_N^2 is plot against the average μ_N, data points from individual animals should collapse onto a straight line with a gradient proportional to the parameter $2\theta \ln 2$ and an intercept at $\ln(1 + n_{\mathrm{TA}})$.

Despite large-scale mouse-to-mouse variation in average clone sizes, a statistical comparison of the data indeed shows convergence towards an approximate linear dependence for both luminal and basal clones (Supplementary Figure 4a,b). From a fit to the data, we found that $\theta = 0.73 \pm 0.18$ (SEM, $R^2 = 0.84$) and $\theta = 0.63 \pm 0.44$ (SEM, $R^2 = 0.82$) for luminal and basal clones, respectively. (Note that, here, fits were made to the last three chase times to avoid the impact of the smallest clones, where size depedencies are distorted by transient behaviours and the influence of the MaSC-descendent unit heirarchy. Moreover, for basal clones, we considered only those mice where there were sufficient clones to achieve statistical confidence.) From the intercept, it is in principle possible to determine the effective size of the descendent unit, $1 + n_{\mathrm{TA}}$. In practice, the fits afford a significant uncertainty in this parameter. Even for the luminal compartment, which has the vast majority of clones, we obtained $n_{\mathrm{TA}} = 0.9 \pm 1.1$ (SEM), with a 95% confidence interval that extends to 3 cells or more. Together, from the fit value of θ, these findings indicate a relatively most degree of expansion through the oestrous cycle where, following activitation, the renewing population within a clone expands by a factor of around 2 or less.

A feature of the linear scaling of (μ_N, σ_N^2) is that it enables the number of oestrous cycles that individual mice have experienced since induction to be estimated. More precisely, by projecting the data points (μ_N, σ_N^2) for individual mice onto the nearest point on the best-fit linear dependence (12), one can infer an effective "age", which can then be translated into an oestrous cycle number by multiplying by an overall scaling factor. To determine the

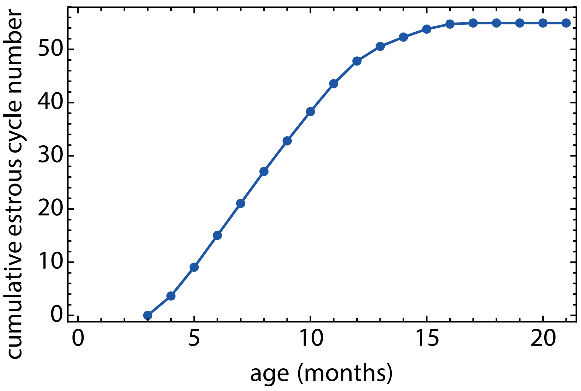

Figure 5: Cumulative oestrous cycle number as a function of age, inferred from data reported in Ref. 2. Starting from around 3 to 4 months, the oestrous cycle has a near-constant rate which attenuates to zero after around 12 months. Note that the majority of induction times used in this study are made between 10-15 weeks of age, around the onset of the first oestrous cycle.

scaling factor, we can turn to literature estimates of how oestrous cycle number changes with age on average. In particular, it is found that, in mice, the oestrous cycle rate ramps up progressively from 3 months of age reaching its maximum value of around 6 cycles per month at 5-6 months.[2] Then, after 12 months, the cycle rate progressively diminishes reaching zero at around 18 months. Since the clones were induced in young adult mice (around 3 months of age), comparison of the cycle rate suggests that, at the longest time point of 550 days, mice have experienced some 60 oestrous cycles on average (see Supplementary Figure 5). Therefore, taken together, from the projection of data points (μ_N, σ_N^2) onto the linear curve combined with the overall scaling $f_{\rm scal}$, the effective oestrous cycle number N is determined as

$$N = \frac{f_{\rm scal}}{1 + (2\theta \ln 2)^2} \left(\mu_N - \ln(1 + n_{\rm TA}) + (2\theta \ln 2)^2 \sigma_N^2 \right) . \tag{13}$$

In the following, we have made use of the luminal clone sizes as the reference data to assign oestrous cycle number for individual mice since the abundance of luminal clones greatly exceeds that of basal, minimising the potential impact of statistical noise. From a comparison of the 550 day data with the estimated total cycle number, we set the scaling factor to $f_{\rm scal} = 25$. However, we note that, while a different value would rescale the overall scale of the rate dependences below, it would not change their relative values.

Variance $\sigma_{\mathbf{N}}^{\mathbf{2}}$: Beginning with the variance of the logarithm of clone size, the zero-dimensional model predicts a linear dependence on oestrous cycle number (5), with

$$\sigma_N^2 = \kappa_\sigma N \tag{14}$$

where $\kappa_\sigma \equiv 2\tilde{q}(\theta \ln 2)^2$. Comparison of the experimental data revealed a remarkably linear size dependence for both luminal and basal clones, with $\kappa_\sigma = 0.042 \pm 0.002$ (SEM, $R^2 = 0.96$) and 0.044 ± 0.005 (SEM, $R^2 = 0.90$), respectively (Figure 3c, left). The fact that these two parameters take roughly equal values lends additional confidence in the theoretical phenomenology, suggesting that the differential expansion of basal and luminal stem cells proceeds at a quan-

[2]Nelson et al., *A Longitudinal Study of Estrous Cyclicity in Aging C57BL/6J Mice: I. Cycle Frequency, Length and Vaginal Cytology.* Biol Reprod **27**, 327–339 (1982).

titatively similar rate, i.e., for luminal and basal clones, the local expansion rate θ, as well as the effective activation rate $\tilde{q}$, take similar values. (Note that although there are two lineage restricted luminal compartments, ER+ and ER−, and only one basal, the total number of cells in the luminal compartment outnumber the basal in the ratio of roughly 2 to 1, explaining why the clonal growth characteristics emerge as quantitatively similar.) These results also establish the consistency of the assumption used in the theoretical analysis that the parameter combination $\tilde{q}\theta^2 \sim 0.04$ is small.

Average $\mu_{\mathbf{N}}$: For the average of the logarithm of clone size, the model predicts a linear dependence on the oestrous cycle number (7), with

$$\mu_N = \kappa_\mu N + \ln(1 + n_{\mathrm{TA}})\,, \tag{15}$$

where $\kappa_\mu \equiv \tilde{q}\theta \ln 2$. Once again, analysis of the average clone size shows an approximately linear dependence, with $\kappa_\mu = 0.033 \pm 0.004$ (SEM, $R^2 = 0.97$) and 0.031 ± 0.010 (SEM, $R^2 = 0.94$) for luminal and basal clones, respectively (Figure 3d, left). From the intercept, we obtain $\ln(1 + n_{\mathrm{TA}}) = 1.6 \pm 0.6$ (SEM) and 1.0 ± 0.8 (SEM) for luminal and basal clones, respectively, with a 95% confidence interval extending to $n_{\mathrm{TA}} = 3$ cells, as before.

Implications and consistency checks: From these results, it follows that for both luminal and basal clones $\theta = \frac{1}{2\ln 2}\frac{\kappa_\sigma}{\kappa_{\mathrm{av}}} \simeq 1$ and the parameter $\beta \equiv \theta \ln 2(1 + \theta \ln 2) \simeq 1$, i.e., during each cycle, once activated, if the clone is not lost, the constituent stem cells undergo approximately one round of duplication. It further follows that

$$\tilde{q} = \frac{\kappa_\sigma}{2(\theta \ln 2)^2} \simeq 0.05\,.$$

Imposing the homeostatic condition, and solving for p and q, we find that $p \simeq 0.5$ and $q \simeq 0.1$, i.e., stem cells become active around once per 10 cycles, on average, with each event leading with 50% probability to stochastic expansion or complete clone loss. (Note that the apparent mouse-to-mouse variability in the average growth dynamics can be associated with the number of oestrous cycles experienced by individual animals, as assumed in the analysis above. However, equally, such variability could be associated with systematic differences in the effective probabilities p and q, i.e., the probability that an individual site will become active during the oestrous cycle, or that a clone at that site will become lost during regression. In this sense, the figures inferred here should be interpreted as ensemble average estimates, while variation between animals and glands would require a temporal analysis within an individual gland, a programme beyond the scope of the current study.)

From these estimates, it then follows from the model that the **fraction of undivided stem cell clones**, P_{single}, is predicted to decay as e^{-N/n_0}, where $n_0 = 1/q(1-p) \simeq 20$. Broadly consistent with this prediction, analysis of the luminal clonal data showed an exponential-like decay of the undivided (single-cell) clone fraction, with a fit to the data suggesting a decay constant of n_0 in the range (3,20) (95% confidence interval) with a best fit average of $n_0 \simeq 12 \pm 4$ cells (SEM, $R^2 = 0.81$) (Extended Data Fig. 8f). Interestingly, from a fit of the experimental data to the predicted exponential dependence, P_{single}, we found that the extrapolation to zero time does not equate to unity, as would be expected if only slow-cycling MaSC were labelled on induction. Instead, for the luminal clones, for example, the intercept is around 80%. This implies that, following induction, some 20% of clones enter very rapidly into cycle, much faster than the predicted average rate of stem cell activation. It is possible likely that these "fast-responders" may represent an induction bias towards stem cells that are primed for activation

following tamoxifen administration.

Based on these results, we could estimate the predicted growth in **average clone size**, $\langle \mathbf{n} \rangle$. Once equilibrated (i.e., when stem cell clones bear their full complement of MaSC-descendant cell progenies), the model predicts that the average size of surviving clones grows exponentially with cycle number N, Eq. (10), with the exponent $\tilde{q}\beta \simeq 0.05$. Once again, when compared to the data, we found a close agreement between the experimental data and the predictions of the phenomenological theory (Extended Data Fig. 8e). Indeed, a fit of the data to $\ln\langle n \rangle_{\text{surv}} = \ln(1 + n_{\text{TA}}) + \tilde{q}\beta N$ gives an independent estimate of $\tilde{q}\beta = 0.047 \pm 0.004$ (SEM, $R^2 = 0.99$) for luminal clones, very close to the inferred value from the statistical measures above.

Finally, we could use the results above to estimate the **surviving clone fraction**, $\mathbf{P_{surv}}$. After all of the TA cell-derived clones (i.e., those derived from MaSC-descendant cells) have been lost, the latter is predicted to decay as $e^{-pqN}/(1 + n_{\text{TA}}) \equiv e^{-N/n_1}/(1 + n_{\text{TA}})$. Taking the parameter estimates from above, this suggests a decay constant of $n_1 = 1/pq \simeq 20$. Although large-scale mouse-to-mouse variability in the induction frequency makes a comparison uncertain, such a drop was not inconsistent with experimental observation (Figure 2d).

3. Experimental fits: *Brca1;Trp53* confetti mutant

We now turned to consider the same experimental measures for the *Brca1;Trp53* confetti clones. Note that, here, we have excluded from consideration mutant clones that have already undergone transformation, which are infrequent in the luminal population and negligible in the basal.

Distribution of clone size: Once again, despite significant mouse-to-mouse variability (Supplementary Figure 6), both luminal and basal mutant clone sizes fit well with a log-normal clone size dependence over the 225 day time course (Supplementary Figure 7). The KDL values for luminal clones are as follows:

time (d)	mouse id.	clones	KDL val.
14	black	115	0.067
	blue	227	0.062
	green	90	0.24
	red	146	0.22
64	black	76	0.10
	blue	123	0.055
	green	91	0.071
	red	88	0.11
120	black	84	0.074
	blue	19	0.073
	green	117	0.076
	red	56	0.085
	purple	95	0.37
225	black	20	0.21
	blue	15	0.38
	green	32	0.25
	red	35	0.054
	purple	12	0.18

time (d)	mouse id.	clones	KDL val.
14	black	46	0.14
	blue	43	0.13
	green	22	0.89
	red	18	0.71
64	black	12	0.41
	blue	23	0.15
	green	16	0.27
	red	6	0.056
120	black	8	0.22
	blue	2	0.017
	green	10	0.13
	red	11	0.25
	purple	8	0.19
225	black	11	0.19
	blue	4	0.10
	green	4	0.46
	red	8	0.22

Statistical correlation between clone size average and variance: Once again, to account for the mouse-to-mouse variability, we first determined whether the inferred average and

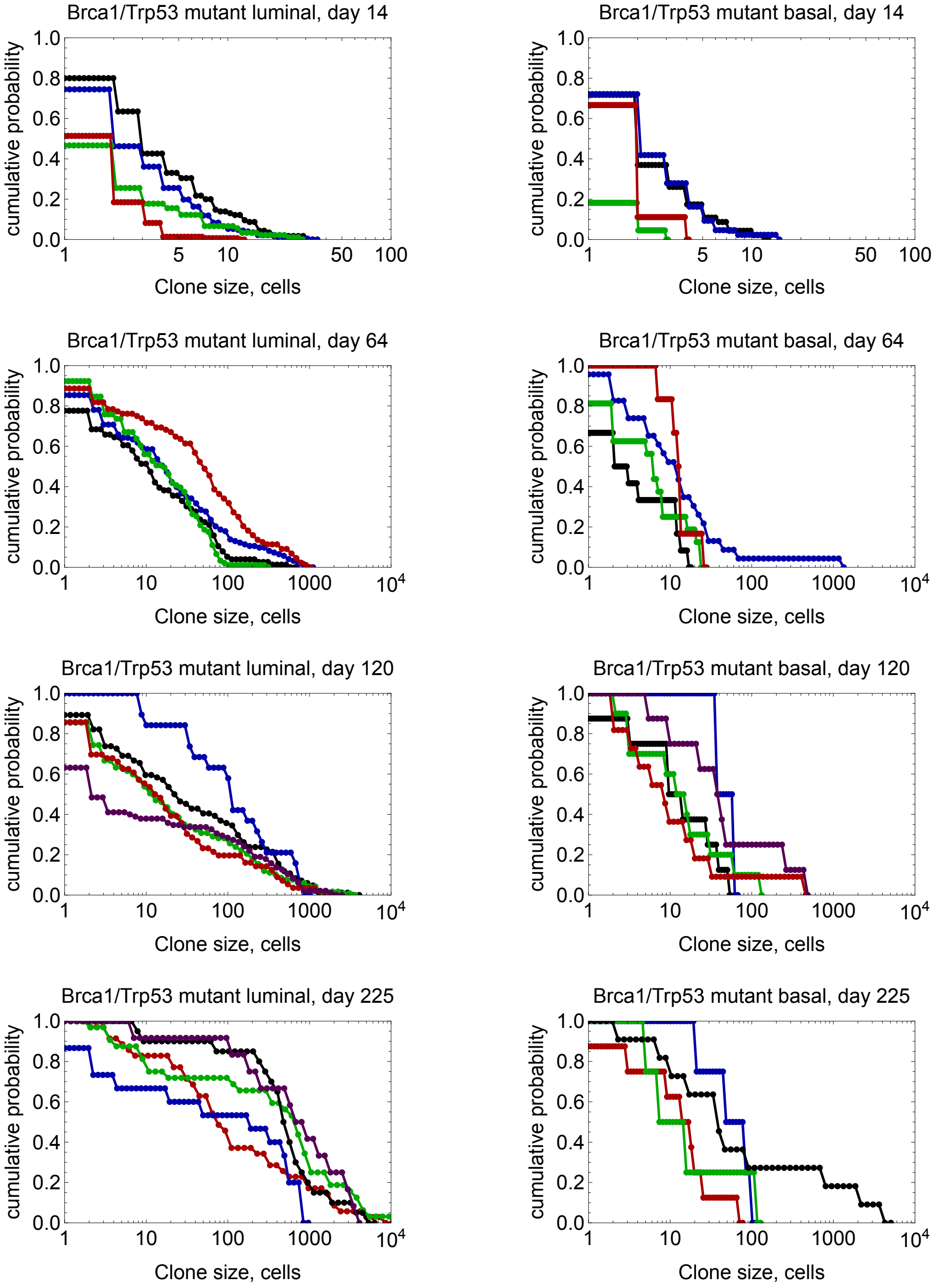

Figure 6: Cumulative size distribution of luminal (left) and basal (right) clones obtained from *Brca1/Trp53* mutant animals across the given range of time points. Different animals are marked by different colours, with the same colour used to depict the luminal and basal clones from the same mouse at a given time point. Note that the level of basal cell induction was lower than luminal and in some cases no basal clones were found. As with the WT confetti animals, note also the very high degree of mouse-to-mouse variability, which becomes increasingly pronounced at the longer time points.

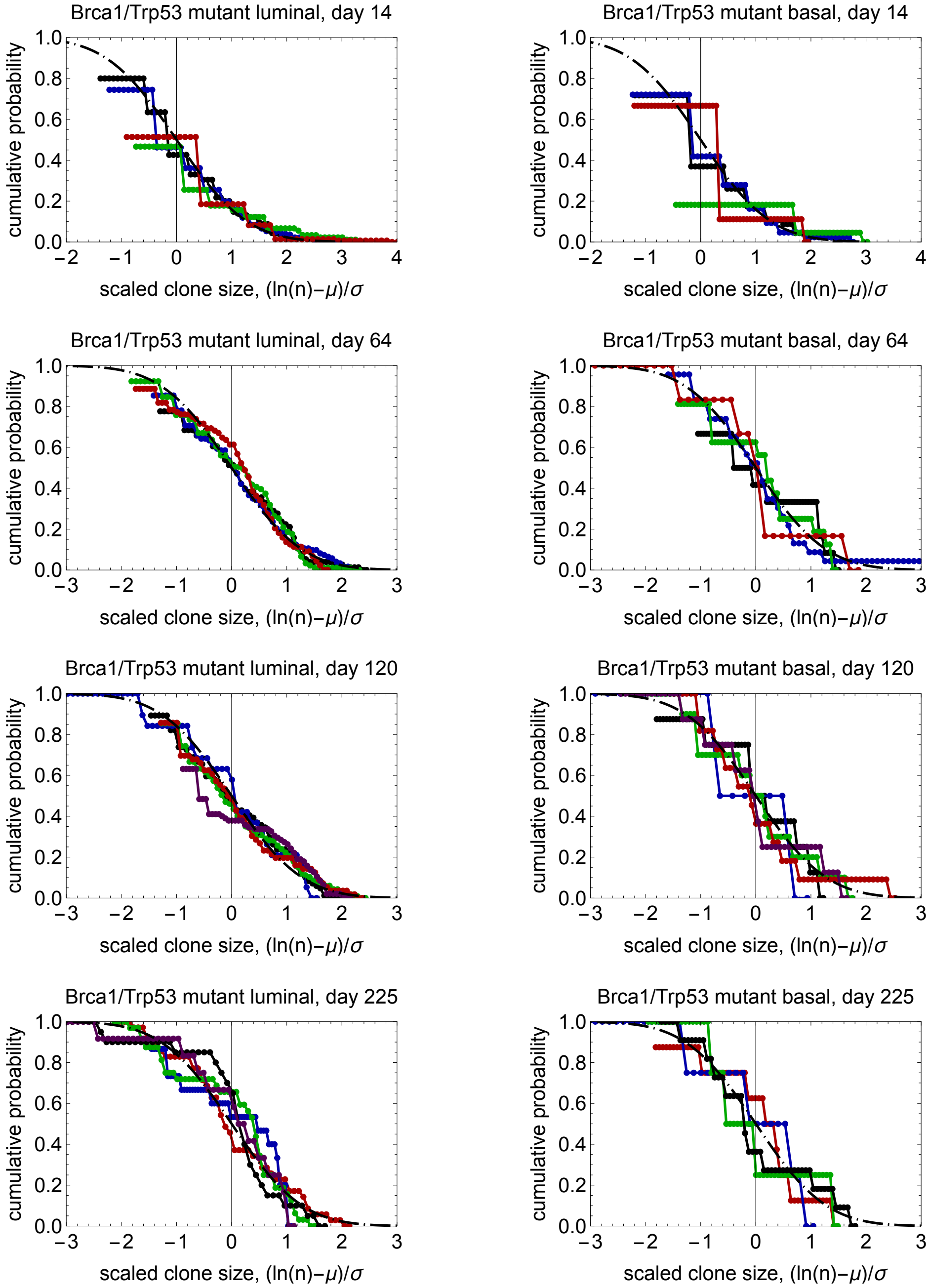

Figure 7: Cumulative size distribution of luminal (left) and basal (right) clones obtained from *Brca1/Trp53* mutant animals across the given range of time points plot as a function of the rescaled size $(\ln n - \mu_a(t))/\sigma_a(t)$, where $\mu_a(t) \equiv \langle \ln n \rangle$ represents the ensemble average of the logarithm of clone sizes for mouse a at chase time t and $\sigma_a(t) = \langle (\ln n - \mu_a(t))^2 \rangle^{1/2}$ denotes the corresponding standard deviation. Different animals are marked by different colours, with the same colour used to depict the luminal and basal clones from the same mouse at a given time point. Note that, when compared across time points and across mice within individual time points, the clone size data collapse onto a log-normal dependence with the scaling function $\mathcal{C}(x) = (1/2)\,\mathrm{erfc}(x/\sqrt{2})$, where $\mathrm{erfc}(x)$ denotes the complementary error function (dashed). For the statistical test of convergence, we refer to the Supplementary Theory text where KDL values are shown.

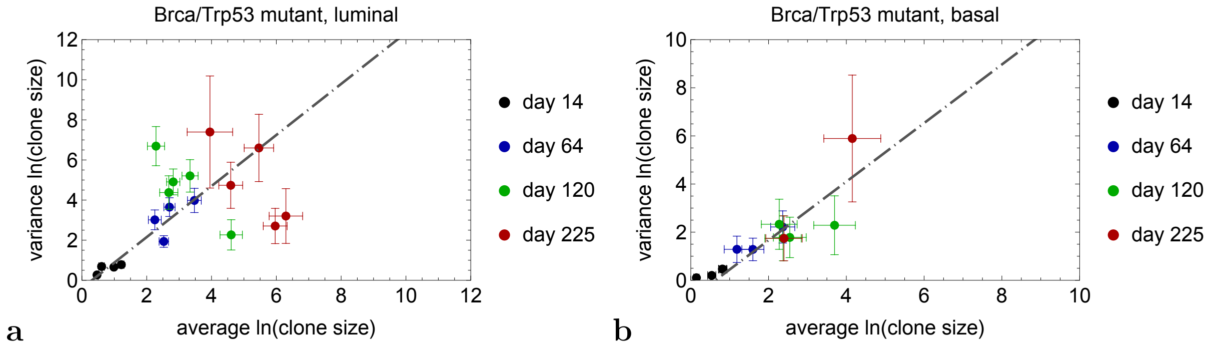

Figure 8: **a,b,** Plot of the variance against the average of the logarithm of luminal (left) and basal (right) clone size of *Brca1;Trp53* mutant mice. Colours denote chase times with day 14 denoted black, day 64 as blue, day 120 as green and day 225 as red. Each dot denotes an individual mouse with bars denoting standard errors. The line shows a fit of the data to a linear dependence, as predicted by the minimal zero-dimensional model (12). For details of the fit values and goodness of fit, we refer to Supplementary Theory text.

variance of the logarithm of clone sizes (μ_N, σ_N^2) for individual mice scaled in proportion. Here, to make the fits, we placed emphasis on data from the 14 and 64 day time points, where the log-normal dependence is most robust. From a fit to the data, we found that $\theta = 0.9 \pm 0.1$ (SEM, $R^2 = 0.96$) and $\theta = 0.9 \pm 0.3$ (SEM, $R^2 = 0.91$) for luminal and basal clones, respectively (Supplementary Figure 8a,b). (Note that, for basal clones, we considered only those mice where there were sufficient clones to achieve statistical confidence.) As with the WT confetti data, we could use this relation to infer an effective age of clones based on the projection of the individual points (μ_N, σ_N^2) onto the linear scaling relation of μ_N and σ_N^2, Eq. (13), focusing of the luminal clones, which were the most abundant. Here, using the tabulated values of average oestrous cycle number from the literature, we use the scaling factor $f_{\text{scal}} = 9$ so that, at the 225 day time point, the accumulated oestrous cycle number is around 40.

Variance σ_N^2: Using this assignment, from a linear fit to the variance of the logarithm of clone size, we could infer a growth rate $\kappa_\sigma = 0.12 \pm 0.01$ (SEM, $R^2 = 0.97$) and 0.067 ± 0.011 (SEM $R^2 = 0.75$) for luminal and basal clone sizes, respectively (Figure 3c, right), consistent with the dynamics predicted by the zero-dimensional model albeit with a gradient elevated significantly over that of the WT confetti control. Moreover, the expansion of the luminal compartment proceeds at almost twice the rate of the basal, pointing to a significant proliferative advantage of the former over the latter during rounds of oestrous cycle-drive growth and regression.

Average μ_N: Similarly, the average of the logarithm of clones size shows a linear-like expansion with the inferred oestrous cycle number with a gradient $\kappa_\mu = 0.083 \pm 0.018$ (SEM, $R^2 = 0.90$) and 0.069 ± 0.012 (SEM, $R^2 = 0.94$) for luminal and basal clones, respectively (Figure 3d, right). In this case, from the intercept of the luminal clone data, we obtain $n_{\text{TA}} = 1.0 \pm 1.2$ (SEM) with 95% confidence interval extending to 4 cells, similar to that obtained for the WT confetti control.

Taken together, these results are consistent with *Brca1;Trp53* mutant clone growth conforming to the same pattern of behaviour as the WT confetti system, albeit with an elevated growth characteristic. In the *Brca1;Trp53* mutant, labelled cells experience a small proliferative advantage with the average expansion parameter θ being raised from around 0.7 in wild-type to around 0.9 for both luminal and basal cells, i.e., during each phase of oestrous cycle, mutant

cells have a marginal growth advantage over their wildtype neighbours. Moreover, for both luminal and basal cells, the parameter $\tilde{q}$ is also elevated by a factor of around 2 over wildtype. If the rate at which MaSCs becomes activated remains the same, at $q = 0.1$, this would imply that the extinction probability $p \simeq 0$, i.e., in contrast to wild-type, once activated the probability of clone loss becomes vanishingly small. Such behaviour is consistent with the action of tumour suppressor gene loss, which may inhibit the natural apoptosis programmes that arise during the regression phase.

Clone growth at ducts vs. ductal ends: To close this section, for completeness we consider the potential impact of the location of clones on their dynamics as well as the impact of pregnancy. Specifically, we compared the dynamics of clones located along the length of the ducts against those that were positioned at ductal ends. Focussing on the 225 day time point, we found that the large-scale mouse-to-mouse variation in clone sizes did not show a positional dependence in WT confetti clones, with both data sets converging to a log-normal size dependence (Supplementary Figure 9a). A similar behaviour was found in the *Brca1;Trp53* mutant confetti model, though the abundance of non-transformed clones at the duct-ends was small (Supplementary Figure 9b). However, notably, the duct-ends showed a higher propensity for transformation, while small clones seemed less abundant than along the ducts, suggesting a potential impact of environmental cues (see main text).

Impact of pregnancy on clone dynamics: Similarly, we considered the potential impact of pregnancy on the clonal dynamics of *Brca1;Trp53* mutant confetti clones focussing on the 120 day time point. Notably, while *Brca1;Trp53* mutations increased the average size of luminal clones over that of the WT confetti animals regardless of pregnancy, the growth of mutant clones appeared to be suppressed during pregnancy compared to nulliparous animals (see main text). Nevertheless, pregnancy did not alter the overall pattern of clone dynamics with the clonal data converging towards a log-normal scaling dependence (10).

While the minimal zero-dimensional model gives a good account of the clonal dynamics in the WT and *Brca1;Trp53* mutant confetti model, careful inspection of the data reveals departures of the clone size distribution from a log-normal dependence at the largest clone sizes. This departure is apparent both in the control data as well as the mutant - see, e.g., the WT confetti data and the *Brca1;Trp53* mutant data from day 225 (Supplementary Figure 3, red and magenta luminal clones and Supplementary Figure 7, black, red, green and magenta luminal clones). Notably, in such cases, the tail of the clone distribution extends to sizes in excess of 100s and even 1000s of cells (Supplementary Figure 1 and Supplementary Figure 6). To address the origin of this departure, it is necessary to question how the zero-dimensional emerges from a more realistic spatial model, and how such a zero-dimensional approximation may break down. It is to this point that we now turn.

4. Spatial cell-based model of epithelial turnover

Previously, we developed a minimal zero-dimensional model that could explain the origin of the log-normal clone size distribution as the result of collective cell amplification and loss mediated by the changes that take place through the oestrous cycle. However, within this framework, clones were assumed to evolve cooperatively and collectively, a behaviour that becomes difficult to justify at long times when clone sizes become larger than the size of the activated domains. Therefore, to gain a deeper understanding into the cellular dynamics and explain potential departures from log-normality at the largest clone sizes, we sought to develop a *spatial* cell-

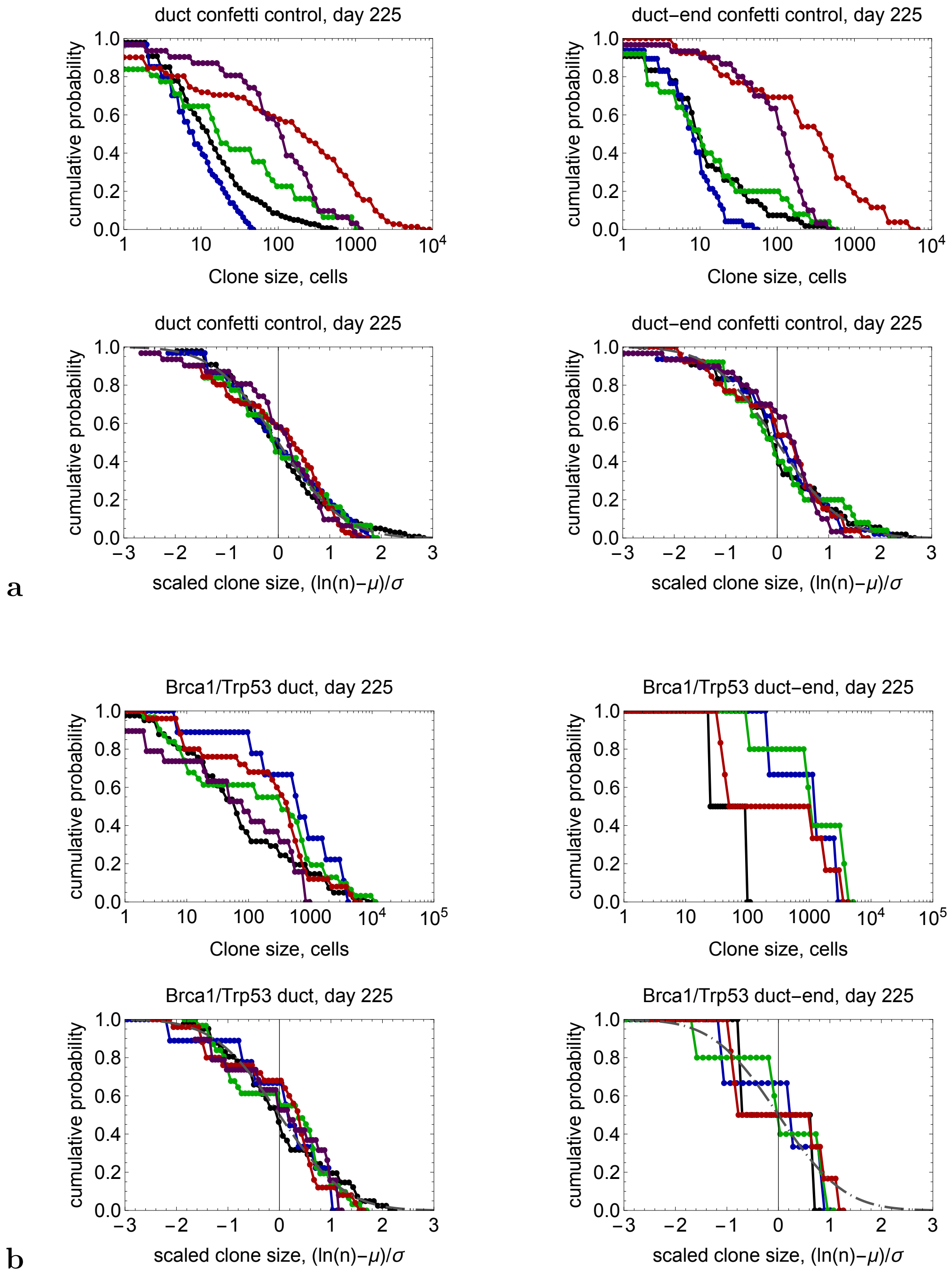

Figure 9: **a,b,** Cumulative distribution of luminal clone size for WT (a) and *Brca1;Trp53* mutant (b) confetti animals at the 225 day time point. The upper panels show the clone size distribution for different animals labelled by a different colour. The left-hand panels show clones positioned along the ducts and the right-hand panels from clones located at ductal ends with the same colour used for clones belonging the same mouse. The lower panels show the cumulative distribution after rescaling, with the dashed line showing the predicted log-normal dependence. Note the departure from a log-normal dependence at the largest clone sizes. Also note that the *Brca1;Trp53* mutant shows evidence for the suppression of small clones.

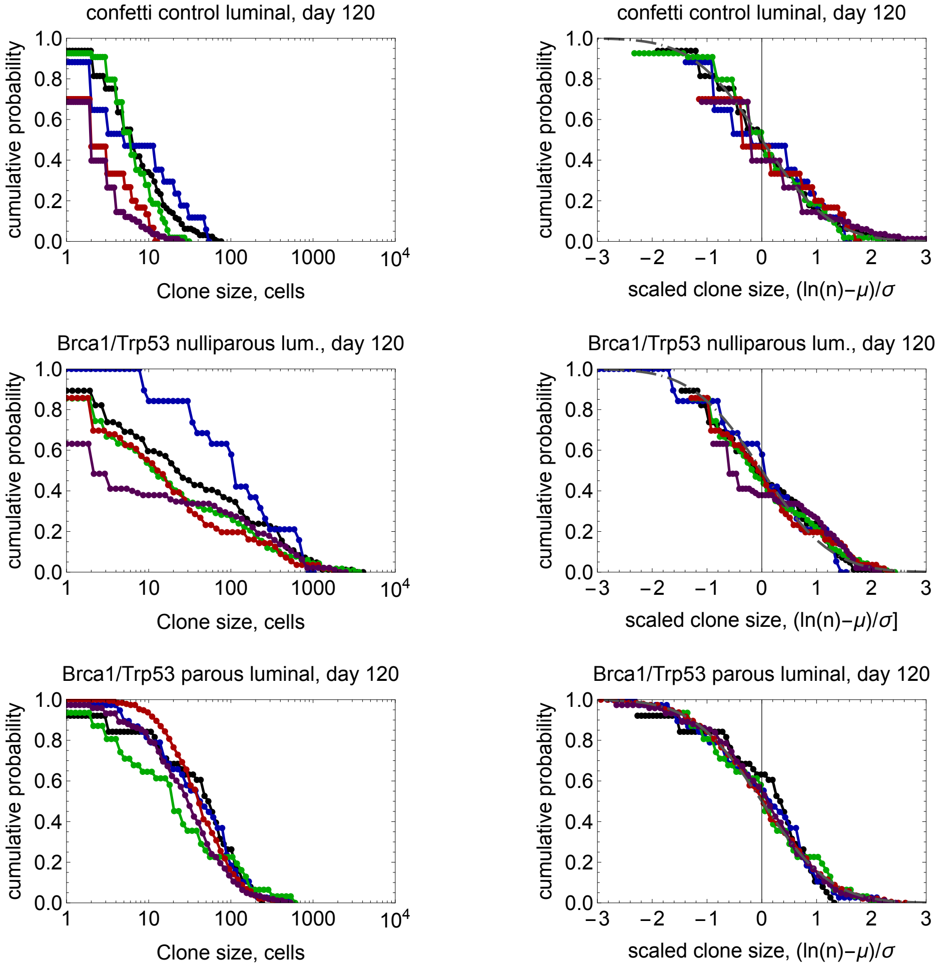

Figure 10: Cumulative distribution of luminal clone sizes for WT (top) and *Brca1;Trp53* mutant nilliparous (middle) and parous (bottom) confetti animals at the 120 day time point. The left-hand panels show the clone size distribution for different animals labelled by a different colour. The right-hand panels show the cumulative distribution after rescaling, with the dashed line showing the the log-normal dependence.

based model that reflects more faithfully the geometry of the ducts and could recapitulate key aspects of the observed clonal dynamics. In particular, we were interested in whether the spatial geometry can either promote or inhibit the process of field clonalization in either WT or non-neutral mutant conditions.

To develop the spatial model, we considered a minimal one-dimensional system, reflecting the geometry of the ductal epithelium (see schematic in Extended Data Fig. 6a and Extended Data Fig. 9b). (An extension to a more realistic quasi one-dimensional arrangement is straightforward, but would complicate the analysis without adding significant further insight.) In line with the phenomenology above, we proposed that the system plays host to an equipotent population of MaSCs, focusing for simplicity on just one of the basal or luminal subcompartments. To mimic the effects of growth and regression during each round of oestrous cycle, we proposed that continuous non-overlapping domains of size ℓ sites become activated stochastically with a probability $q = 0.1$ chosen to match that obtained from the fit of the data to the phenomenological model. In this process, cells from the central $\ell/2$ sites were altogether lost (viz. the $p = 0.5$ probability found from the fits above) and replaced through the duplication and expansion of MaSCs on the $2 \times \ell/4$ sites that border the depleted region. To avoid commensurability effects, and consistent with the heterogeneity in clone sizes seen in the short-term (14 day) clonal data, we allowed for an additional $\ell/4$ rounds of stochastic loss and replacement events to occur between neighboring MaSCs, chosen at random within the locally expanded population.

Taking a domain size of $\ell = 1000$ sites and a system size of 10^6 sites, stochastic simulation showed that the lattice model could reproduce quantitatively the observed exponential-like increase in the average size of surviving clones as well as the log-normal dependence of the clone size distribution (Extended Data Fig. 9g). Although the cell-based model predicted the emergence of a log-normal clone size dependence, when clones expand beyond the size of a single activated domain, ℓ, the size distribution was seen to decay much more rapidly, revealing a narrower Gaussian-like clone size dependence (see Extended Data Fig. 9h). Such behavior is straightforwardly understood: When clones exceed the domain size, their further expansion and contraction occurs in a stepwise manner on the boundaries of the clone. (Note that, if the activated region occurs entirely within a clone, loss and replacement takes place wholly within the labelled cell population resulting in no net change in clone size.) In this limit, the clone dynamics become equivalent to a one-dimensional neutral drift process involving local stochastic loss and replacement, similar to that found in the context of mouse spermatogenesis in seminiferous tubules (Klein et al., 2010). Although the particular dependence of the clone size distribution is not available in analytical form, we can gain some insight from the behaviour of the neutral drift model. In one-dimension, the clone size distribution takes the long-term scaling form (Klein et al., 2010; Klein and Simons, 2011),

$$P(n,t) = \frac{\pi n}{2\langle n(t)\rangle} \exp\left[-\frac{\pi}{4}\left(\frac{n}{\langle n(t)\rangle}\right)^2\right],$$

where $\langle n(t)\rangle \sim \sqrt{t}$, with t denoting the time since clone induction. Since, in the current context, clones enter into this long-term regime at different times following induction, the clone distribution for the lattice model is not straightforward to infer. However, based on the Gaussian nature of the 1d distribution, we can expect that the clone growth characteristic changes rapidly from log-normal with the size distribution becoming heavily truncated at the largest sizes, as seen in the numerical distributions (Extended Data Fig. 9h).

Based on these insights, we then turned to the consider the experimental clone data to

see whether there was evidence for truncation of the log-normal distribution at the largest clone sizes. Notably, while the majority of the data sets fit well with a log-normal clone size dependence, several animals both from the WT and *Brca1;Trp53* confetti models showed evidence for a departure at the largest sizes. Notably, careful inspection of the data shows that for clones in excess of several hundred cells, the size distribution becomes truncated more abruptly than a log-normal distribution would predict (cf. luminal clones from the black and magenta WT confetti data at the day 225 time point in Extended Data Fig. 9i, and luminal clones from the red and green *Brca1;Trp53* mutant confetti data in Extended Data Fig. 9j). However, note that such behaviour becomes visible only in the tails of the distribution when plot on a logarithmic scale.

Here, for simplicity, we have supposed that, following Brca1 and Trp53 mutation, all mutant cells are equipotent. In reality, the transition in clone behavior may be progressive, as cells acquire further sporadic mutations, leading in turn to the transition to invasive carcinoma. However, our aim here was to understand whether the scale of the predicted and observed transition in the dynamics of spreading could be captured from a minimal model. The analysis above shows that the observed heterogeneity in the mutant clone sizes can be explain quantitatively purely as a result of geometrical constraints on the growth characteristics.

5. Clone dynamics in the epithelium of ovariectomized mice

Finally, we turn to consider the dynamics of mammary clones in female mice that have been ovariectomized under WT and *Brca1;Trp53* mutant conditions. Here, consistent with the absence of the large-scale modelling of the epithelium that occurs through rounds of oestrous cycle, clones are strongly reduced in size over those of the unperturbed animals.

Beginning with the analysis of clones from WT confetti animals, the average clone size shows only a modest increase over the 225 day time course, with an early phase of growth showing a plateau at the longest time point (data not shown). At the same time, the cumulative clone size distribution shows evidence of a biexponential-like distribution with a sharp exponential-like drop at small clones sizes (3-4 cells or less) transitioning to a shallower exponential-like dependence at larger clone sizes in both the basal and luminal compartment (Extended Data Fig. 12a,b). Such behaviour would be consistent with that expected for a renewing MaSC population supporting a small set of MaSC-descendant cells and undergoing a slow process of local stochastic loss and replacement of neighboring stem cells along the ductal epithelium: For small clone sizes, the size dependence would be dominated by individual partially-filled MaSC-descendant cell units while, at larger clone sizes, the distribution would converge towards the hallmark exponential-like size dependences expected for a system undergoing local stochastic stem cell loss and replacement along the epithelium (Klein and Simons, 2011).

In clones mutant for *Brca1;Trp53*, we observed a qualitatively similar type of behaviour, with a progressive linear-like expansion of the average clone size over the 225 day time course (data not shown). Moreover, the cumulative clone size distribution shows a biexponential-like dependence similar to that of the WT confetti system but with a distribution that extends to larger clone sizes (Extended Data Fig. 12c,d). Overall, such behaviour is consistent with *Brca1;Trp53* mutant confetti clones experiencing a small proliferative advantage over their wild-type neighbors, but not sufficient to drive large-scale field expansion or, indeed, transformation to an invasive state.
